# A Randomized Study of Peginterferon Lambda-1a Compared to Peginterferon Alfa-2a in Combination with Ribavirin and Telaprevir in Patients with Genotype-1 Chronic Hepatitis C

**DOI:** 10.1371/journal.pone.0164563

**Published:** 2016-10-17

**Authors:** Robert Flisiak, Mitchell Shiffman, Juan Arenas, Hugo Cheinquer, Igor Nikitin, Yuping Dong, Khurram Rana, Subasree Srinivasan

**Affiliations:** 1 Department of Infectious Diseases and Hepatology, Medical University of Bialystock, Bialystock, Poland; 2 Bon Secours Liver Institute of Virginia, Richmond, Virginia, United States of America; 3 Department of Gastroenterology and Hepatology, University Hospital Donostia, San Sebastián, Spain; 4 Department of Internal Medicine, Universidade Federal do Rio Grande do Sul, Porto Alegre, Brazil; 5 Department of Higher Level General Medicine, Russian State Medical University, Moscow, Russia; 6 Research and Development, Bristol-Myers Squibb, Inc., Wallingford, Connecticut, United States of America; Taipei Veterans General Hospital, TAIWAN

## Abstract

**Background:**

A randomized, double-blind, multinational, phase 3 study was conducted comparing the efficacy and safety of peginterferon lambda-1a (Lambda)/ribavirin (RBV)/telaprevir (TVR) vs. peginterferon alfa-2a (Alfa)/RBV/TVR in patients with chronic hepatitis C virus (HCV) genotype-1 (GT-1) infection.

**Methods:**

Patients (treatment-naïve or relapsers on prior Alfa/RBV treatment) were randomly assigned in a 2:1 ratio to receive Lambda/RBV/TVR or Alfa/RBV/TVR. Total duration of treatment was either 24 or 48 weeks (response-guided treatment), with TVR administered for the first 12 weeks. The primary endpoint was the proportion of patients who achieved a sustained virologic response at post treatment week 12 (SVR12), which was tested for noninferiority of Lambda/RBV/TVR.

**Results:**

A total of 838 patients were enrolled, and 617 were treated; 411 and 206 patients received Lambda/RBV/TVR and Alfa/RBV/TVR, respectively. The majority of patients were treatment-naïve, with HCV GT-1b and a high baseline viral load (≥800,000 IU/mL). Less than 10% of patients had cirrhosis (Lambda, 7.5%; Alfa, 6.8%). Lambda/RBV/TVR did not meet the criterion for noninferiority (lower bound of the treatment difference interval was -12.3%); the SVR12 in all patients (modified intent-to-treat) was 76.2% in the Lambda arm and 82.0% in the Alfa arm. Overall, the frequency of adverse events in each arm was comparable (Lambda, 91.7%; Alfa, 97.1%). As expected based on the safety profile of the 2 interferons, there were more hepatobiliary events observed in the Lambda arm and more hematologic events in the Alfa arm.

**Conclusions:**

In this comparison of Lambda/RBV/TVR and Alfa/RBV/TVR in patients who were treatment-naïve or had relapsed on prior Alfa/RBV treatment, Lambda failed to demonstrate noninferiority based on SVR12 results. Treatment with Lambda/RBV/TVR was associated with a higher incidence of relapse. More patients discontinued Lambda/RBV/TVR treatment during the first 4 weeks of study treatment, mainly due to hepatobiliary-related events, and more Lambda patients were lost to follow-up.

## Introduction

Until recently, the basis of treatment for infection with chronic hepatitis C virus (HCV) involved the use of a type I interferon (IFN), peginterferon alfa-2a or -2b (Alfa), plus ribavirin (RBV); however, these regimens are associated with treatment-limiting hematologic and systemic toxicity [1], due largely to the expression of the type I IFN receptor complex on a wide variety of nonhepatic cell types [2,3]. In 2003, type III (lambda) IFNs were identified [4,5]. Lambda IFNs have antiviral activity similar to that of type I IFNs [2,4,6], however their receptor complexes are expressed on a more limited subset of host cells, suggesting treatment with IFN lambda may be associated with fewer systemic adverse events (AEs) [2,3]. Clinical trials assessing the efficacy and safety of peginterferon lambda-1a (Lambda)-based regimens for treatment of chronic HCV infection have shown improved overall tolerability, along with similar efficacy, compared to Alfa-based regimens [7].

In 2011, regulatory authorities in the United States and in many countries in Europe approved 2 direct-acting antivirals (DAAs), telaprevir (TVR) [8] and boceprevir (BOC) [9], for use in combination with Alfa plus RBV for the treatment of IFN/RBV-naïve and -experienced patients with genotype-1 (GT-1) chronic HCV infection. Response-guided treatment using TVR or BOC, each in combination with Alfa/RBV, was shown to reduce treatment duration and improve sustained virologic response (SVR) rates in treatment-naïve and -experienced patients [10–14]. Although these new combination regimens demonstrated improved efficacy, they were not without both tolerability and resistance challenges.

At the time this study was initiated, IFN-based alternatives to Alfa with the potential for improved tolerability and efficacy were being developed for use in combination with RBV with or without a DAA. Based on the improved safety profile of Lambda vs. Alfa and the improved efficacy of Alfa plus RBV when combined with a DAA [10–14], the combination of Lambda/RBV/DAA was of interest. To investigate this option, Lambda and Alfa, each administered in combination with RBV plus TVR (TVR selected because of its wider usage at the time), were evaluated in this study to compare their safety and efficacy profiles in patients with chronic HCV GT-1 infection.

## Materials and Methods

### Trial design

This was a randomized, double-blind, multinational, phase 3 study in patients with GT-1 chronic HCV infection (treatment-naïve or relapsed following prior Alfa/RBV treatment) (supporting CONSORT checklist is available as supporting information; see S1 Checklist). Patients were randomly assigned 2:1 to receive Lambda (180 μg by subcutaneous [SC] injection once weekly) or Alfa (180 μg by SC injection once weekly), each administered in combination with RBV (1000 mg per day orally for patients weighing <75 kg and 1200 mg per day orally for patients weighing ≥75 kg) plus TVR (750 mg orally 3 times daily) during the first 12 weeks, followed by either Lambda/RBV or Alfa/RBV for a total treatment duration of 24 or 48 weeks depending on the achievement or not, respectively, of an extended rapid virologic response (eRVR; defined as undetectable HCV RNA at weeks 4 and 12 of treatment). Patients with cirrhosis completed a total of 48 weeks of treatment, regardless of their eRVR status. At the completion of treatment, all patients were to receive off-treatment follow-up for 48 weeks. Randomization was stratified according to interleukin-28B (*IL28B*) host GT (CC vs. non-CC), treatment-naïve versus prior relapse status, and HCV GT (1a vs. 1b), and was performed by a sponsor-designated center via an Interactive Voice Response System using a block size of 6. Both site and subjects were blinded to treatment for the entire study. A designated member of the study staff at each investigative site was unblinded and responsible for dispensing study medication.

### Patients

The study population was comprised of adults (aged ≥18) who were naïve to prior anti-HCV therapy (including IFN and DAA), or prior relapsers to Alfa/RBV, with GT-1a or -1b chronic HCV infection and HCV RNA ≥100,000 IU/mL. Patients with cirrhosis as determined by either liver biopsy indicating METAVIR score F4 or equivalent, or by FibroScan^®^ score ≥14.6 kPa were eligible to participate. After the trial started, the protocol was amended to exclude patients with significant portal hypertension (eg, hepatic venous pressure gradient [HVPG] ≥10 mmHg, splenomegaly ≥12 cm (diameter), and a Fibroscan score >21 kPa) who could be at an increased risk of developing hepatic decompensation during treatment. Patients who were co-infected with hepatitis B virus or human immunodeficiency virus (HIV), had other medical conditions contributing to chronic liver disease, had current or prior evidence of portal hypertension, or had previous exposure to a DAA were excluded from participating.

The study was conducted in accordance with Good Clinical Practice (GCP), as defined by the International Conference on Harmonization (ICH) and in accordance with the ethical principles underlying the European Union Directive 2001/20/EC and the United States Code of Federal Regulations, Title 21, Part 50 (21CFR50). The study protocol and any amendments were approved by the institutional review board/independent ethics committee at each site (Protocol and IRBs available as supporting information; see S1 Protocol and S1 IRBs), and all patients provided written informed consent. This study has been registered at ClinicalTrials.gov, identifier NCT01598090 (https://clinicaltrials.gov/ct2/show/NCT01598090?term=nct01598090&rank=1)

### Endpoints and assessments

The primary endpoint was the proportion of patients who achieved SVR at post treatment week 12 (SVR12). Secondary efficacy endpoints included: the proportion of patients who achieved rapid virologic response (RVR; defined as undetectable HCV RNA at week 4 of treatment); eRVR; end of treatment response (EOTR); and the proportion of treatment-naïve patients who achieved SVR12.

Secondary safety endpoints included the proportion of patients with: rash-related dermatologic events reported during the first 12 weeks of treatment; treatment-emergent cytopenic abnormalities defined as hemoglobin <10 g/dL, absolute neutrophil count <750 x 10^6^/L, and platelet count <50000 x 10^6^/L; on-treatment flu-like symptoms; on-treatment musculoskeletal symptoms; serious adverse events (SAEs); discontinuation due to AEs; dose reductions throughout the study; and laboratory abnormalities. Laboratory abnormalities were measured and graded using the Division of AIDS (DAIDS) Table for Grading the Severity of Adult and Pediatric Adverse Events (grade 1–2, mild to moderate; grade 3–4, severe to potentially life-threatening).

Plasma HCV RNA was assessed using the COBAS^®^ TaqMan^®^ HCV Test v2.0 (Roche Molecular Systems Inc., Pleasanton, California, USA) with a lower limit of quantification (LLOQ) of 25 IU/mL. HCV RNA was assessed at baseline, week 2, every 2 weeks through week 12, then at weeks 16, 20, 24, 36, and 48, and at post-treatment follow-up weeks 4, 12, 24, 36, and 48.

Treatment was discontinued if any of the following futility criteria were met: (1) HCV RNA >1000 IU/mL at week 4 or 12; (2) confirmed HCV RNA detected at week 24; or (3) a virologic breakthrough, defined as a confirmed increase in HCV RNA >1 × log_10_ above nadir or HCV RNA ≥LLOQ after previously having HCV RNA <LLOQ while on treatment.

### Statistical analysis

The primary endpoint, SVR12, was tested for noninferiority between Lambda/RBV/TVR and Alfa/RBV/TVR treatment arms. Power calculations to determine appropriate sample sizes assumed a 79% response rate for both Lambda/RBV/TVR and Alfa/RBV/TVR and a −12% boundary for comparison with the lower limit of the two-sided 95% confidence interval (CI) for the treatment difference (Lambda/RBV/TVR—Alfa/RBV/TVR). Sample sizes of 406 patients treated with Lambda/RBV/TVR and 203 patients treated with Alfa/RBV/TVR, resulted in a 95% power to demonstrate noninferiority of Lambda/RBV/TVR to Alfa/RBV/TVR for the proportion of patients with SVR12.

Analyses were performed using the modified intent-to-treat (mITT) population, which includes all randomized patients who received at least one treatment dose. Patients with missing data for an endpoint were treated as nonresponders. Treatment differences in SVR12 rate and 95% CI were estimated using the stratum-adjusted Mantel-Haenszel approach. All testing was performed at a significance level of 0.05.

## Results

### Patient disposition and baseline characteristics

**Fig 1:**
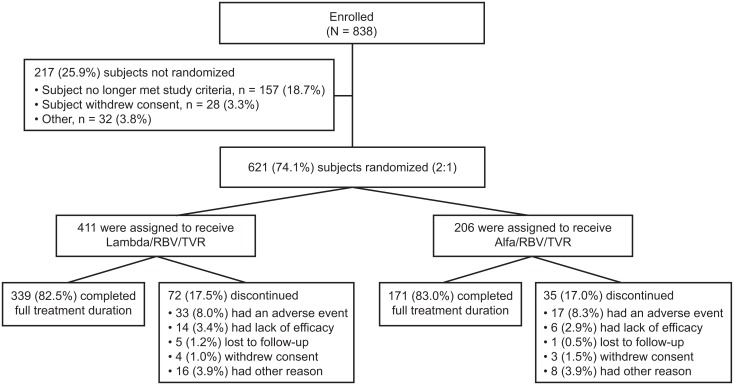
Patient disposition and reasons for discontinuation. RBV, ribavirin; TVR, telaprevir.

A total of 838 patients were enrolled starting in March of 2013, and the last patient visit occurred in February of 2015. Following enrollment, 217 patients were excluded from randomization for reasons detailed in Fig 1. Of the 621 patients randomized (2:1), 617 were treated: 411 received Lambda/RBV/TVR, and 206 received Alfa/RBV/TVR. A similar proportion of patients completed treatment in both study arms (Lambda, 82.5%; Alfa, 83.0%). Because the clinical development of Lambda was discontinued while this study was in progress, the trial was terminated after all patients had received at least 12 weeks of post-treatment follow-up to determine SVR12 but not the initially planned 48 weeks of post-treatment follow-up assessments.

Patient demographic and disease characteristics were comparable between the 2 study arms (Table 1). The majority of patients were white, HCV GT-1b, treatment-naïve, *IL28B* non-CC GT, with a high (≥800,000 IU/mL) baseline viral load. Less than 10% of patients had cirrhosis (Lambda, 7.5%; Alfa, 6.8%). Because more study sites were located in Europe than in North America, enrollment was mostly in Europe.

**Table 1:** Baseline demographic and disease characteristics. HCV, hepatitis C virus; *IL28B*, interleukin 28B; RBV, ribavirin; TVR, telaprevir.

Characteristic	Lambda/RBV/TVR (n = 411)	Alfa/RBV/TVR (n = 206)
Age (years), median (range)	48.0 (19–77)	46.0 (19–71)
**Age category, n (%)**		
<21	5 (1.2)	2 (1.0)
21–64 years	392 (95.4)	197 (95.6)
≥65 years	14 (3.4)	7 (3.4)
**Hepatitis C disease-related characteristics**		
HCV RNA (log_10_ IU/mL)		
Median	6.6	6.6
HCV RNA distribution, n (%)		
<800,000 IU/mL	57 (13.9)	35 (17.0)
≥800,000 IU/mL	354 (86.1)	171 (83.0)
HCV genotype, n (%)		
1a	171 (41.6)	84 (40.8)
1b	240 (58.4)	122 (59.2)
**Prior relapse to peginterferon Alfa/RBV, n (%)**		
Relapser	101 (24.6)	50 (24.3)
Naïve	309 (75.2)	156 (75.7)
Not reported	1 (0.2)	0
**Stage of fibrosis, n (%)**		
None or mild	241 (58.6)	122 (59.2)
Moderate or severe	118 (28.7)	63 (30.6)
Cirrhosis	31 (7.5)	14 (6.8)
Not reported	21 (5.1)	7 (3.4)
***IL28B* genotype, n (%)**		
CC	97 (23.6)	48 (23.3)
CT	242 (58.9)	125 (60.7)
TT	72 (17.5)	33 (16.0)
**Region, n (%)**		
Europe	239 (58.2)	137 (66.5)
North America	115 (28.0)	43 (20.9)
South America	39 (9.5)	19 (9.2)
Asia	18 (4.4)	7 (3.4)

### Key efficacy endpoints

A summary of key efficacy endpoints is shown in Table 2. Lambda/RBV/TVR did not meet the goal of noninferiority; the SVR12 rates (mITT) were 76.2% in patients treated with Lambda and 82.0% in patients treated with Alfa. The lower limit of the two-sided 95% CI for the treatment difference (Lambda—Alfa) was −12.3%, which was less than the prespecified lower bound of −12.0%.

**Table 2:** Summary of key efficacy endpoints. CI, confidence interval; EOTR, end-of-treatment response; eRVR, extended rapid virologic response; mITT, modified intent-to-treat; RBV, ribavirin; RVR, rapid virologic response; SVR12, sustained virologic response at week 12 post-treatment follow-up; TVR, telaprevir.

	Lambda/RBV/TVR (n = 411) Responder/Evaluable n/N (%; 95% CI)	Alfa/RBV/TVR (n = 206) Responder/Evaluable n/N (%; 95% CI)
mITT analyses		
SVR12 (all patients)	313/411 (76.2; 72.0–80.3)	169/206 (82.0; 76.8–87.3)
SVR12 (naïve patients)	229/311 (73.6; 68.7–78.5)	127/155 (81.9; 75.9–88.0)
RVR	275/411 (66.9; 62.4–71.5)	157/206 (76.2; 70.4–82.0)
eRVR	263/411 (64.0; 59.3–68.6)	146/206 (70.9; 64.7–77.1)
EOTR	368/411 (89.5; 86.6–92.5)	187/206 (90.8; 86.8–94.7)

The subset of patients treated with Lambda/RBV/TVR who were treatment-naïve also did not achieve SVR12 noninferiority (Lambda, 73.6%; Alfa, 81.9%; lower bound of the treatment difference interval was -15.8%). The RVR and eRVR rates were both lower in the Lambda arm (Table 2), although the complete early virologic response (cEVR; defined as undetectable HCV RNA at week 12 of treatment) was slightly higher with Lambda (Lambda, 88.1%; Alfa, 85.4%). Based on the eRVR rates, more patients in the Lambda arm than in the Alfa arm had to be treated for 48 weeks. The EOTR was comparable for both groups (Lambda, 89.5%; Alfa, 90.8%). However, there were more relapsers in the Lambda arm than in the Alfa arm (Lambda, 6.0%; Alfa, 4.3%). Virologic breakthrough occurred in 15 patients (3.6%) treated with Lambda versus 4 patients (1.9%) treated with Alfa; most of these 15 patients did not complete treatment.

The SVR12 rates for various patient subgroups, including high (≥800,000 IU/mL), GT-1a, treatment-naïve, baseline cirrhosis, and *IL28B* CC, are shown in Table 3. The SVR12 was comparable or higher following treatment with Alfa than with Lambda in all subgroups except for prior relapsers to Alfa/RBV; in this group, a comparable proportion of patients achieved SVR12 with Lambda (84.2%) compared to Alfa (82.0%).

**Table 3:** Subgroup analysis for SVR12. CI, confidence interval; HCV, hepatitis C virus; *IL28B*, interleukin 28B; RBV, ribavirin; SVR12, sustained virologic response at week 12 post-treatment follow-up; TVR, telaprevir.

	Lambda/RBV/TVR (n = 411) Responder/Evaluable n/N (%; 95% CI)	Alfa/RBV/TVR (n = 206) Responder/Evaluable n/N (%; 95% CI)
**HCV RNA distribution**		
<800,000 IU/mL	54/57 (94.7; 88.9–100.0)	33/35 (94.3; 86.6–100.0)
≥800,000 IU/mL	259/354 (73.2; 68.5–77.8)	136/171 (79.5; 73.5–85.6)
**HCV genotype**		
1a	119/171 (69.6; 62.7–76.5)	65/84 (77.4; 68.4–86.3)
1b	194/240 (80.8; 75.9–85.8)	104/122 (85.2; 79.0–91.5)
**Prior relapse to peginterferon Alfa/RBV**		
Relapser	85/101 (84.2; 77.0–91.3)	41/50 (82.0; 71.4–92.6)
Naïve	228/309 (73.8; 68.9–78.7)	128/156 (82.1; 76.0–88.1)
Not reported	0/1 (0.0; 0.0–0.0)	NA
**Baseline cirrhosis status**		
Present	16/31 (51.6; 34.0–69.2)	10/14 (71.4; 47.8–95.1)
Absent	282/359 (78.6; 74.3–82.8)	153/185 (82.7; 77.3–88.2)
Not reported	15/21 (71.4; 52.1–90.8)	6/7 (85.7; 59.8–100.0)
***IL28B* genotype**		
CC	77/97 (79.4; 71.3–87.4)	39/48 (81.3; 70.2–92.3)
Non-CC	236/314 (75.2; 70.4–79.9)	130/158 (82.3; 76.3–88.2)

### Safety

On-treatment safety data, including AEs (any grade) in ≥20% of patients in any treatment group and SAEs, are summarized in Table 4. The vast majority of patients experienced at least one AE (Lambda, 91.7%; Alfa, 97.1%).

**Table 4:** On-treatment safety. AE, adverse event; ALT, alanine aminotransferase; AST, aspartate aminotransferase; RBV, ribavirin; TVR, telaprevir.

	Lambda/RBV/TVR (n = 411)	Alfa/RBV/TVR (n = 206)
Total number of patients with an AE, n (%)	377 (91.7)	200 (97.1)
**AEs (any grade) in ≥20% patients in any treatment group, n (%)**		
Pruritus	187 (45.5)	100 (48.5)
Nausea	172 (41.8)	67 (32.5)
Fatigue	143 (34.8)	75 (36.4)
Rash	124 (30.2)	59 (28.6)
Insomnia	102 (24.8)	56 (27.2)
Decreased appetite	85 (20.7)	42 (20.4)
Asthenia	81 (19.7)	61 (29.6)
Headache	66 (16.1)	42 (20.4)
Anemia	54 (13.1)	100 (48.5)
Arthralgia	49 (11.9)	43 (20.9)
Myalgia	49 (11.9)	43 (20.9)
Pyrexia	31 (7.5)	53 (25.7)
**Grade 3/4 laboratory abnormalities, n (%)**		
ALT	30 (7.3)	1 (0.5)
AST	48 (11.7)	2 (1.0)
Total bilirubin	75 (18.2)	10 (4.9)
Hemoglobin	11 (2.7)	45 (21.8)
Neutrophils	7 (1.7)	39 (18.9)
Platelets	1 (0.2)	2 (1.0)

Side effects typically associated with Alfa were statistically significantly lower with Lambda than with Alfa, including: emergent laboratory cytopenic abnormalities (Lambda, 11.7%; Alfa, 55.8%; p < 0.0001); flu-like symptoms (Lambda, 14.4%; Alfa 36.4%; p < 0.0001); and musculoskeletal symptoms (Lambda, 21.4%; Alfa, 30.6%; p < 0.0139). However, there was no overall difference between treatment arms with respect to rash-related dermatologic events (Lambda, 36.3%; Alfa, 38.3%), likely due to the use of TVR in both treatment regimens.

A comparable proportion of patients discontinued treatment due to AEs (Lambda, 8.0%; Alfa, 8.3%). However, more patients discontinued treatment due to AEs during the first 4 weeks of therapy with Lambda than with Alfa, which contributed to the lower RVR observed in the Lambda arm.

Serious AE (SAE) rates were comparable across the 2 treatment arms (Lambda, 10.5%; Alfa, 9.7%), with the most common hepatobiliary-related AE, jaundice (considered an important medical event) occurring more frequently in the Lambda arm (Lambda, 2.7%; Alfa, 1.0%) and rash occurring more frequently in the Alfa arm (Lambda, 0.2%; Alfa, 2.4%).

Two deaths were reported, one due to pneumonia (Lambda arm at treatment week 24), which was considered not related to study treatment, and one due to hypoxia (Alfa arm at treatment week 40), which was considered related to study treatment. The latter patient had a long history of tobacco use and a medical history of interstitial lung disease.

Treatment-emergent laboratory abnormalities were generally consistent with the AE profiles for Lambda and Alfa (Table 4). Grade 3/4 elevations of alanine aminotransferase, aspartate aminotransferase, and total bilirubin were more frequent in the Lambda arm, while grade 3/4 cytopenic abnormalities were more frequent in the Alfa arm.

Overall, the proportion of dose reductions for the peginterferon component of the study regimen was comparable for both treatment arms (Lambda, 12.7%; Alfa, 14.1%) but generally occurred for different reasons. In the Lambda arm, dose reductions occurred most commonly as a result of elevated liver function tests (10.2%), while in the Alfa treatment arm, dose reductions occurred most commonly because of hematologic toxicity (12.1%). The majority of RBV dose reductions were for hematologic toxicity and were substantially higher in the Alfa arm (40.8%) compared with the Lambda arm (9.5%).

## Discussion

In this comparison of Lambda/RBV/TVR and Alfa/RBV/TVR in patients with GT-1 chronic HCV infection, Lambda failed to demonstrate noninferiority to Alfa for the primary (SVR12 overall) efficacy endpoint. For the primary endpoint, the overall SVR12 rate for Lambda/RBV/TVR (76.2%) was lower than the SVR12 rate for Alfa/RBV/TVR (82.0%). SVR rates obtained in large, randomized, controlled trials with similar patient populations and similar regimens of Alfa/RBV/TVR have ranged from 72.0% to 83.0% [10–12] and the result from this study for the Alfa arm was in the upper end of this range.

In this study, patients from both treatment groups infected with HCV GT-1b responded with a higher SVR12 rate than those with HCV GT-1a. These results are consistent with published results on response of GT-1 subtypes to IFN/RBV/DAA combinations, including IFN/RBV/TVR [10]. Also consistent with prior observations [15], patients from both treatment groups who had lower baseline HCV RNA levels had higher response rates than those with higher HCV RNA levels. In this study, SVR12 in prior relapsers to Alfa/RBV following retreatment with Lambda/RBV/TVR (84.2%) was higher than in HCV treatment-naive patients and comparable to the rate observed in the Alfa/RBV/TVR treatment arm (82.0%). This observation is consistent with the findings from other studies of Alfa/RBV/TVR therapy in chronic HCV infection, which have shown that response rates are generally higher in patients who relapsed after prior Alfa/RBV therapy than in treatment-naïve patients [10,12]. This observation is not surprising, as prior relapsers represent a relatively homogeneous group of patients who have responded to treatment previously and who would be expected to respond favorably to a retreatment regimen that includes a DAA. Finally, comparable responses were observed in the CC genotype and non-CC genotype patients treated with Alfa/RBV/TVR. *IL28B* genotype is a predictor of treatment response to a non-DAA interferon regimen; therefore, typically higher response rates might have been expected in the CC genotype patients. However, the increasing potency of treatment regimens, which include one or more DAAs, including TVR, has led to a diminished role of *IL28B* status in the prediction of treatment response.

A number of factors may explain the difference in response rates in patients receiving Lambda and Alfa in this study. Patients treated with Lambda experienced more relapses compared with patients treated with Alfa. Slightly more patients treated with Lambda were lost to follow-up during the study (patients lost to follow-up were considered treatment failures in the study analysis). In addition, although baseline disease characteristics were balanced in the study arms, slightly more patients had cirrhosis in the Lambda arm (7.5%) vs. the Alfa arm, (6.8%), and these patients appeared to have a lower SVR12 response.

Even though more relapses may have been observed in the Lambda arm as compared with the Alfa arm in this study, the relapse rate for Lambda is consistent with relapse rates observed in another TVR study [10] and was lower than the relapse rates observed in the phase 2b EMERGE study of Lambda/RBV vs. Alfa/RBV in HCV GT-1 patients (19.6% with Lambda/RBV and 18.4% with Alfa/RBV) [7]. Like other studies with TVR, the addition of TVR reduced the relapse rate when compared with non-DAA interferon regimens.

The RVR with Lambda treatment was lower than with Alfa treatment, suggesting that Lambda was not as efficacious in suppressing HCV within the first 4 weeks of treatment. This finding is not consistent with the Lambda phase 2b EMERGE study, which demonstrated higher RVR rates for Lambda/RBV than for Alfa/RBV and comparable SVR rates [7]. During these first 4 weeks of treatment, discontinuations due to AEs were more frequent in the Lambda arm than in the Alfa arm and more patients in the Lambda arm discontinued due to hepatobiliary-related events, therefore limiting the opportunity for these subjects to achieve virologic control. Hence, the inability to complete the required treatment duration may have negatively impacted both the early viral suppression rates and SVR12 rates for patients receiving Lambda.

In terms of safety, the Lambda and Alfa behaved as expected based on the cellular distribution of their respective receptors: more hepatic-related events (including discontinuations due to AEs) were seen with Lambda, while more hematologic and systemic AEs were seen with Alfa. The frequency of grade 3/4 alanine aminotransferase and aspartate aminotransferase elevations were higher with Lambda (11.7% and 7.3%, respectively) than Alfa (1.0% and 0.5%, respectively) but were also higher than that previously observed with Lambda/RBV (3%) [7]. The reasons for these elevations are unclear.

Although it might be expected that improved tolerability with Lambda would lead to higher response rates as compared with Alfa-based regimens, the goal of Lambda treatment was to provide a regimen that was comparable to Alfa treatment without the treatment-limiting side effects of Alfa, with higher response rates being an added benefit, if observed. In this study, side effects that are typically associated with Alfa treatment such as emergent cytopenic abnormalities, flu-like symptoms, and musculoskeletal symptoms were lower with Lambda treatment. However, higher hepatobiliary-related events occurred with Lambda treatment, which led to early discontinuation of therapy and may have negated any potential benefit of this treatment. The addition of TVR in both groups also contributed to the side effect profile and was consistent with effects as observed in other TVR studies. Regarding central nervous system (CNS) AEs related to IFN therapy, the mechanisms and the specific receptors involved are not well understood. Differences in CNS AEs between groups treated with Lambda and Alfa were not observed in the previous clinical phase 2b study with Lambda; therefore, in the present study, differences were not anticipated, nor were they observed.

Since the development of this study, treatment for chronic HCV infection has substantially evolved, with the approval of next generation, all-oral, IFN-free, direct-acting regimens that achieve high SVR rates with improved tolerability. Due to the diminished role of IFN-based regimens for the treatment of chronic HCV infection, the Lambda clinical development program has been discontinued. However, Lambda may be investigated to treat other viral infections such as chronic hepatitis D virus (HDV) infection. Moreover, the results of this study broaden the safety and efficacy information for Lambda as a therapeutic agent and are important to understanding the biology and tolerability of type III IFNs and the potential clinical importance of Lambda beyond HCV treatment. Although type I IFNs (alfa and beta) and type III IFNs (lambda) activate the same intracellular signaling pathway and possess many of the same biological activities, alfa receptors are broadly expressed on most cell types, while lambda receptors outside of the liver are largely restricted to cells of epithelial origin and plasmacytoid dendritic cells [16]. As a result, treatment with Lambda was expected to provide improved tolerability with comparable SVR rates. However, results of the present study show that when Lambda and TVR are combined in a treatment regimen, any potential benefit based on biology may be offset by a more complicated safety profile. Although Lambda is no longer being evaluated as a therapeutic alternative to Alfa for the treatment of HCV infection, Lambda may be investigated for treatment of other diseases of infectious or noninfectious etiology.

## Conclusions

In this comparison of Lambda/RBV/TVR and Alfa/RBV/TVR for SVR12 in patients who were treatment-naïve or relapsers on prior treatment with Alfa/RBV, Lambda failed to demonstrate noninferiority. The safety profiles, as anticipated, were hepatobiliary-related in the Lambda arm, while those receiving Alfa had more of hematologic and systemic toxicities that are commonly associated with Alfa treatment.

## Supporting Information

S1 Checklist (DOC)

S1 Protocol (PDF)

S1 IRBs (DOC)

## References

[1] Ghany MG, Nelson DR, Strader DB, Thomas DL, Seeff LB. An update on treatment of genotype 1 chronic hepatitis C virus infection: 2011 practice guideline by the American Association for the Study of Liver Diseases. Hepatology. 2011; 54: 1433–1444. 10.1002/hep.24641 21898493 PMC3229841

[2] Zhou Z, Hamming OJ, Ank N, Paludan SR, Nielsen AL, Hartmann R. Type III interferon (IFN) induces a type I IFN-like response in a restricted subset of cells through signaling pathways involving both the Jak-STAT pathway and the mitogen-activated protein kinases. J Virol. 2007; 81: 7749–7758. 10.1128/JVI.02438-06 17507495 PMC1933366

[3] Doyle SE, Schreckhise H, Khuu-Duong K, Henderson K, Rosler R, Storey H, et al Interleukin-29 uses a type 1 interferon-like program to promote antiviral responses in human hepatocytes. Hepatology. 2006; 44: 896–906. 10.1002/hep.21312 17006906

[4] Kotenko SV, Gallagher G, Baurin VV, Lewis-Antes A, Shen M, Shah NK, et al IFN-lambdas mediate antiviral protection through a distinct class II cytokine receptor complex. Nat Immunol. 2003; 4: 69–77. 10.1038/ni875 12483210

[5] Sheppard P, Kindsvogel W, Xu W, Henderson K, Schlutsmeyer S, Whitmore TE, et al IL-28, IL-29 and their class II cytokine receptor IL-28R. Nat Immunol. 2003; 4: 63–68. 10.1038/ni873 12469119

[6] Dumoutier L, Tounsi A, Michiels T, Sommereyns C, Kotenko SV, Renauld JC. Role of the interleukin (IL)-28 receptor tyrosine residues for antiviral and antiproliferative activity of IL-29/interferon-lambda 1: similarities with type I interferon signaling. J Biol Chem. 2004; 279: 32269–32274. 10.1074/jbc.M404789200 15166220

[7] Muir AJ, Arora S, Everson G, Flisiak R, George J, Ghalib R, et al A randomized phase 2b study of peginterferon lambda-1a for the treatment of chronic HCV infection. J Hepatol. 2014; 61: 1238–1246. 10.1016/j.jhep.2014.07.022 25064437

[8] INCIVEK™ (telaprevir) [prescribing information]. Cambridge, MA; Vertex Pharmaceuticals Inc.; October 2013.

[9] VICTRELIS^®^ (boceprevir) [prescribing information]. Whitehouse Station, NJ; Merck & Co., Inc.; August 2015.

[10] Jacobson IM, McHutchison JG, Dusheiko G, Di Bisceglie AM, Reddy KR, Bzowej NH, et al Telaprevir for previously untreated chronic hepatitis C virus infection. N Engl J Med. 2011; 364: 2405–2416. 10.1056/NEJMoa1012912 21696307

[11] Sherman KE, Flamm SL, Afdhal NH, Nelson DR, Sulkowski MS, Everson GT, et al Response-guided telaprevir combination treatment for hepatitis c virus infection. N Engl J Med. 2011; 365: 1014–1024. 10.1056/NEJMoa1014463 21916639 PMC3809077

[12] Zeuzem S, Andreone P, Pol S, Lawitz E, Diago M, Roberts S, et al Telaprevir for retreatment of HCV infection. N Engl J Med. 2011; 364: 2417–2428. 10.1056/NEJMoa1013086 21696308

[13] Poordad F, McCone J Jr, Bacon BR, Bruno S, Manns MP, Sulkowski MS, et al Boceprevir for untreated chronic HCV genotype 1 infection. N Engl J Med. 2011; 364: 1195–1206. 10.1056/NEJMoa1010494 21449783 PMC3766849

[14] Bacon BR, Gordon SC, Lawitz E, Marcellin P, Vierling JM, Zeuzem S, et al Boceprevir for previously treated chronic HCV genotype 1 infection. N Engl J Med. 2011; 364: 1207–1217. 10.1056/NEJMoa1009482 21449784 PMC3153125

[15] Zhu Y, Chen S. Antiviral treatment of hepatitis C virus infection and factors affecting efficacy. World J Gastroenterol. 2013; 19: 8963–8673. 10.3748/wjg.v19.i47.8963 24379621 PMC3870549

[16] Donnelly RP, Kotenko SV. Interferon-lambda: a new addition to an old family. J Interferon Cytokine Res. 2010; 30: 555–564. 10.1089/jir.2010.0078 20712453 PMC2925029

