# Supplementary material for: A Randomized Study of Peginterferon Lambda-1a Compared to Peginterferon Alfa-2a in Combination with Ribavirin and Telaprevir in Patients with Genotype-1 Chronic Hepatitis C
Source: PLoS One. 2016 Oct 17;11(10):e0164563. doi: 10.1371/journal.pone.0164563 (PMC5066958; doi:10.1371/journal.pone.0164563)
Supplement: S1 Protocol — (PDF) [file pone.0164563.s002.pdf]

Protocol Number: AI452020
IND Number: 100,420
Ex-US Non-IND
EUDRACT Number 2011-004695-11
Date: 05-Apr-2012
Revised Date: 19-Jun-2013

Clinical Protocol AI452020

A Phase 3 Blinded Randomized Study of Peginterferon Lambda-1a and Ribavirin Compared to Peginterferon Alfa-2a and Ribavirin, Each Administered with Telaprevir in Subjects with Genotype-1 Chronic Hepatitis C who are Treatment-naïve or Relapsed on Treatment with Peginterferon Alfa and Ribavirin

Revised Protocol Number: 02
Incorporates Amendment: 03

Medical Monitor
Simon Portsmouth, MD FRCP
Bristol-Myers Squibb
5 Research Parkway
Wallingford, CT 06492
Telephone (office): 203-677-7006
Fax: 203-677-6852

Study Director
Khurram Rana, Pharm D
Bristol-Myers Squibb
5 Research Parkway
Wallingford, CT 06492
Telephone (office): 203-677-3657
Fax: 203-677-6852

24-hr Emergency Telephone Number
USA: 1-866-470-2267
International: +1-248-844-7390

Bristol-Myers Squibb Research and Development
5 Research Parkway
Wallingford, CT 06492
United States
and
Avenue de Finlande 4 B-1420
Braine-l' Alleud, Belgium

This document is the confidential and proprietary information of Bristol-Myers Squibb Company and its global affiliates (BMS). By reviewing this document, you agree to keep it confidential and to use and disclose it solely for the purpose of assessing whether your

organization will participate in and/or the performance of the proposed BMS-sponsored study. Any permitted disclosures will be made only on a confidential "need to know" basis within your organization or to your independent ethics committee(s). Any other use, copying, disclosure or dissemination of this information is strictly prohibited unless expressly authorized in writing by BMS. Any supplemental information (eg, amendments) that may be added to this document is also confidential and proprietary to BMS and must be kept in confidence in the same manner as the contents of this document. Any person who receives this document without due authorization from BMS is requested to return it to BMS or promptly destroy it. All other rights reserved.

Replace all previous version(s) of the protocol with this revised protocol and please provide a copy of this revised protocol to all study personnel under your supervision, and archive the previous versions.

Approved v4.0 930056810 4.0

DOCUMENT HISTORY

Document	Date of Issue	Summary of Change
Revised Protocol 02	19-Jun-2013	Incorporates amendment 03.
Amendment 03	19-Jun-2013	The amendment clarifies the duration of treatment for all cirrhotics is 48 weeks, stratification applies for all subjects, inclusion/exclusion criteria, the rash management plan for telaprevir, that HCV RNA results will be made available to sites and staff at the end of treatment and during follow-up, and the anlysis of the secondary endpoint for Part A is for all subjects in Part A. Some typographical errors have also been corrected.<br>This amendment also includes adding medications to the list of contraindicated medications with telaprevir, adding laboratory tests for lipids and triglycerides, and adding an exclusion criterion for potassium.<br>Additional exclusion criteria specific to cirrhotic subjects has also been added. Compensated cirrhotic without evidence of portal hypertension will be eligible for the study. Indicators of significant portal hypertension such as hepatic venous pressure gradient (HVPG) ≥ 10 mm Hg, splenomegaly ≥ 12 cm (diameter), and a Fibroscan score > 21 kPa are being included as exclusion criteria. Relevant data evaluating portal hypertension and other relevant clinical and laboratory data for cirrhotic subjects must be reviewed by the BMS Central Medical Monitor prior to randomization in order to help minimize the inclusion of subjects with significant portal hypertension who could be more at risk of developing hepatic decompensation during treatment.
Revised Protocol 01	25-Feb-2013	Incorporates amendment 02.
Amendment 02	25-Feb-2013	Cover page, Synopsis and amendment 01, title, change Peginterferon Alfa-2a to Peginterferon Alfa to clarify the regimen for those who have relapsed. To read as follows; "A Phase 3 Blinded Randomized Study of Peginterferon Lambda-1a and Ribavirin Compared to Peginterferon Alfa-2a and Ribavirin, Each Administered with Telaprevir in Subjects with Genotype-1 Chronic Hepatitis C who are Treatment-naïve or Relapsed on Treatment with Peginterferon Alfa and Ribavirin"<br>Cover page, change of medical monitor and study director.<br>Synopsis: Clarify primary and secondary objectives, study design, and statistical objectives for Part A and Part B of the study.<br>Section 1, Introduction and Study Rational, update and clarify background.<br>Section 1.1 Study Rational: change Peginterferon Alfa-2a to Peginterferon Alfa to clarify the regimen for those who have relapsed.<br>Section 1.3.3 Exploratory Objectives, clarify exploratory objectives.<br>Section 1.5 Overall Risk/Benefit Assessment; add paragraph under Potential Benefits of Lambda Treatment to add further information.<br>Section 3.1.1 Part A: Open Label Sentinel; clarify Treatment Period and Follow-up Period. Replace Figure 3.1.1 to reflect updated Part A Follow-up Period. Delete first paragraph after Go/No-go Decision to Part B to reflect updated Part A to B transition plans.<br>Section 3.1.2 Part B: Randomized, Controlled, Double-Blinded Cohort; clarify Treatment Period and Follow-up Period. Replace Figure 3.1.2 to reflect updated Part B Follow-up Period.<br>Section 3.1.3 Futility Criteria for Parts A and B; add section to provide

Approved v4.0 930056810 4.0

Document	Date of Issue	Summary of Change
		clarity regarding efficacy futility criteria, rather than this information appearing as footnotes in Figures 3.1.1 and 3.1.2.<br>Section 3.3 Study Population; add paragraph to clarify target population and add information regarding re-screening.<br>3.3.1 Inclusion Criteria, 2) Target Populations; add Fibroscan as methodology for determining cirrhosis.<br>3.3.1 Inclusion Criteria, 3) Age and Reproductive Status, clarify acceptable means of contraception.<br>Section 3.3.2 Exclusion Criteria, 2) Medical History and Concurrent Diseases; add exclusion for clinically significant ophthalmologic disorders upon eye examination, severe chronic obstructive lung disease, lactating or breastfeeding subjects, and prophylactic use of antibiotics, anti-fungals or anti-virals within 14 days of enrollment, confirmed uncontrolled hypertension, and QTcF > 500 mSec; clarify history of psychiatric disease. Move criteria from 4) Medical History or Laboratory Findings that Exlude Subject from Alfa, RBV or TVR Therapy to 2) Medical History and Concurrent Diseases.<br>Section 3.4.1 Prohibited and/or Restricted Treatments; clarify use of erythropoetic growth factors.<br>Section 3.4.1.1 Prohibited/Restricted during Dosing of TVR, clarify use of systemic corticosteriods.<br>Section 3.4.2 Other Restrictions and Precautions, deleted concomitant medication assessment as this is recorded elsewhere.<br>Section 3.5 Discontinuation of Subjects from Treatment, move efficacy futility criteria to Section 3.1.3 for clarity, add post-treatment follow-up information to reflect updated study design, add Section 3.5.1 Laboratory/Clinical Criteria for Treatment Discontinuation for clarification purposes.<br>Section 4.3 Selection and Timing of Dose for Each Subject, add instructions for missed Lambda, alfa-2a, and RBV doses, add Duration of Treatment information.<br>Section 4.3.1 Dose Modifications, clarify guidelines for modifications due to liver abnormalities.<br>Table 4.3.1.1A Dose Reductions for Lambda or alfa-2a; add Dose Line indications for each column heading.<br>Section 4.3.1.2 Depression Monitoring and Dose Modifications, clarify the use of the C-SSRS assessment.<br>Section 4.3.1.4 Telaprevir: added additional safety guidelines per the updated telaprevir label.<br>Section 4.3.2 Dose Interruptions, clarify rational and process for dose interruptions.<br>Section 4.4 Blinding/Unblinding, add additional information to clarify blinding of HCV RNA results.<br>Section 5.1 Flow Chart/Time and Events Schedule, Table 5.1A Screening Procedural Outline (AI452020); add FibroScan, Vital Signs/Weight/Height, Eye exam (including retinal exam), Serum sample for genetic biomarker (IL28), and HCV RNA back-Back up sample.<br>Table 5.1C Procedural Outline Off Treatment (AI452020); clarify collection of SAEs and separate weeks 35 and 48 visits.

Approved v4.0 930056810 4.0

Document	Date of Issue	Summary of Change
		Table 5.3.1 Laboratory Assessments; add FSH to screening visit.<br>Section 5.3.1 Laboratory Assessments, 3rd paragraph; add information on HCV RNA analysis at central laboratory and information about addition HCV genotype analyses.<br>Section 5.3.5 Anemia Management for TVR-Treated Subjects, clarify use of erythropoetic growth factors.<br>Section 5.3.6.3 Rash Treatment, clarify use of systemic corticosteriods.<br>Section 5.3.6.4 Rash Management for TVR, clarify resumption of study drug dosing.<br>Section 5.3.7 Serious Skin Reactions; add section per updated telaprevir label.<br>Section 5.4.1 Primary Efficacy Assessment, clarify the antiviral assessments for Parts A and B.<br>Section 5.6.1 Patient Health Questionnaire (PHQ-9), add recommended further evaluation for subjects with treatment-emerging depression.<br>Section 5.8.1 Resistance Monitoring. Add information regarding unblinding of viral load to internal BMS scientist for selection of samples for resistance testing at selected time points.<br>Section 5.9 Results of Central Assessments: delete section as ECGs will no longer be sent to a central reader.<br>Section 6.1.1 Serious Adverse Event Collection and Reporting; clarify timing and duration for SAE reporting.<br>Section 6.6 Potential Drug Induced Liver Injury, added recommended follow-up for discontinuations due to liver abnormalities.<br>Section 8.3.1 Primary Endpoints; clarify definition of primary endpoints for Part A and Part B<br>Section 8.3.2 Secondary Endpoint(s), clarify endpoints for Part A and Part B.<br>Section 8.3.3 Other Secondary Endpoints, add section to clarify secondary endpoints.<br>Section 8.3.4 Exploratory Endpoint(s), clarify endpoints.<br>Section 8.4.2 Efficacy Analyses, clarify and add analyses for IL28B rs12979860 host genotype (CC, non-CC), using modified ITT.<br>Section 8.4.4 Pharmacokinetic Analyses; define and clarify timing and analyses.<br>Section 8.4.5 Biomarker Analyses; add unblinding of bioanalytical personnel of treatment arm for samples selection for analyses.<br>Section 8.5 Interim Analyses; include additional interim analyses for Parts A and B.<br>Section 11 List of Abbreviations; add FSH Follicular Stimulating Hormone.
Original Protocol	05-Apr-2012	Not applicable

Approved v4.0 930056810 4.0

SYNOPSIS

Clinical Protocol AI452020

Title of Study: Protocol AI452020: A Phase 3 Blinded Randomized Study of Peginterferon Lambda-1a and Ribavirin Compared to Peginterferon alfa-2a and Ribavirin, Each Administered with Telaprevir in Subjects with Genotype-1 Chronic Hepatitis C who are Treatment-naïve or Relapsed on Treatment with Peginterferon Alfa and Ribavirin

Investigational Product(s), Dose and Mode of Administration, Duration of Treatment with Investigational Product(s):

Peginterferon lambda-1a (BMS-914143, Lambda) solution for subcutaneous (SC) injection, 180 μg once weekly for a maximum of 48 weeks (Part A: Open Label; Part B: Double Blind)

Peginterferon alfa-2a (Pegasys®, alfa-2a) solution for SC injection, 180 μg once weekly for a maximum of 48 weeks (Part B: Double Blind)

Ribavirin (RBV, Ribasphere) Tablet, 1000 mg per day orally for subjects weighing < 75 kg, and 1200 mg per day orally for subjects weighing ≥ 75 kg, each for a maximum of 48 weeks (Part A and Part B: Open Label)

Telaprevir (TVR) Film Coated Tablet, 750 mg, orally 3 times a day for a maximum of 12 weeks (Part A and Part B: Open Label)

Key Efficacy Terminology:

The Roche COBAS® TaqMan HCV Test v.2.0 (lower limit of quantitation [LLOQ] = 25 IU/mL; limit of detection ~ 10 IU/mL for HCV GT-1 subtype) will be used to measure HCV RNA levels. When the titer result is target not detected, the results will be reported by our laboratory as, “HCV RNA not detected.” When the titer result is < 25 IU/ml, the results will be reported as “HCV RNA < 25IU/ml, target detected”

Response definitions for this study are:

- RVR (Rapid Virologic Response): HCV RNA < LLOQ target not detected at Week 4 of treatment
- eRVR (Extended Rapid Virologic Response): HCV RNA < LLOQ target not detected at Weeks 4 and 12 of treatment
- EVR (Early Virologic Response): ≥ 2 $\log_{10}$ decrease in HCV RNA from baseline, at Week 12 of treatment or < LLOQ (target detected or not detected) at Week 12 of treatment
- cEVR (Complete Early Virological Response): HCV RNA < LLOQ target not detected at Week 12 of treatment
- Virologic Breakthrough:
  a) confirmed > 1 $\log_{10}$ increase in HCV RNA over nadir OR
  b) confirmed HCV RNA ≥ LLOQ after previously having an HCV RNA level of < LLOQ (target detected or target not detected) while on treatment.

  Measurements must be confirmed within 2 weeks of the original result.
- SVR4 (Sustained Virologic Response at post-treatment follow-up Week 4): HCV RNA < LLOQ (target detected or not detected) at Week 4 of post-treatment follow-up
- SVR12 (Sustained Virologic Response at post-treatment follow-up Week 12): HCV RNA < LLOQ (target detected or not detected) at Week 12 of post-treatment follow-up
- SVR24 (Sustained Virologic Response at post-treatment follow-up Week 24): HCV RNA < LLOQ (target detected or not detected) at Week 24 of post-treatment follow-up
- Relapse: HCV RNA < LLOQ, target not detected at the end of treatment followed by HCV RNA ≥ LLOQ at any post-treatment follow-up visit.

Approved v4.0 930056810 4.0

Study Phase: 3

Research Hypothesis: Treatment with a regimen of Lambda combined with RBV and TVR (Lambda/RBV/TVR) will result in comparable efficacy and better tolerability than treatment with alfa-2a/RBV/TVR in treatment-naïve subjects and relapsers with genotype-1 (GT-1) chronic hepatitis C (HCV) infection.

Primary Objective:

Part A: Single Arm, Open Label Sentinel Cohort

To evaluate efficacy as measured by eRVR and safety (as measured by the frequency of deaths, SAEs, drug related AEs, dose reductions and discontinuations due to AEs) of Lambda/RBV/TVR in a sentinel cohort of subjects with GT-1chronic HCV infection who are treatment-naïve or who relapsed on a prior alfa/RBV treatment regimen.

Part B: Randomized Controlled, Double Blinded Cohort

To evaluate efficacy as measured by SVR12 of Lambda/RBV/TVR compared to alfa-2a/RBV/TVR in subjects with GT-1 chronic HCV infection who are treatment-naïve or who relapsed on prior alfa/RBV treatment regimen.

Secondary Objective:

Part A:

- Evaluation of efficacy as measured by proportion of subjects who achieve SVR12
- Evaluation of efficacy as measured by proportion of subjects who achieve SVR24

Part B:

- Evaluation of efficacy as measured by proportion of subjects who achieve SVR12 in treatment-naive subjects
- Evaluation of safety of Lambda/RBV/TVR compared to alfa-2a/RBV/TVR, as measured by the reduction in treatment-emergent cytopenic abnormalities (anemia is defined by HB < 10 g/dL, neutropenia as defined by ANC < 750 mm^3, thrombocytopenia as defined by platelets < 50,000 mm^3)
- Evaluation of efficacy as measured by proportion of subjects who achieve eRVR
- Evaluation of safety as measured by the following on-treatment interferon-associated symptoms following treatment with Lambda/RBV/TVR compared to alfa-2a/RBV/TVR:
    - Flu-like symptoms (as defined by pyrexia or chills or pain)
    - Musculoskeletal symptoms (as defined by arthralgia or myalgia or back pain)
- Evaluation of efficacy as measured by proportion of subjects who achieve SVR24

Study Design:

AI452020 is a Phase 3 clinical study in GT-1 chronic HCV infected subjects who are treatment-naive or who are prior relapsers to alfa/RBV combination treatment. This study has two parts. Part A is a single arm, open label sentinel cohort. Part B is a randomized controlled, double-blinded cohort. All subjects will be treated for 24 or 48 weeks.

Part A: Open Label Sentinel Cohort

Part A is an open-label, single arm study involving approximately 25 subjects with GT-1 chronic HCV infection. Subjects will be treated with Lambda/RBV/TVR (see Figure 1). Treatment-naive subjects with HCV GT-1b subtype will be capped at approximately 50%. Relapsers will be capped at approximately 20%. Subjects with compensated cirrhosis will be capped at approximately 10%.

Approved v4.0 930056810 4.0

Treatment Period:

On Day 1, subjects will start treatment with Lambda/RBV/TVR.

- TVR will only be administered for the first 12 weeks of treatment
- Cirrhotic and non-cirrhotic subjects achieving eRVR (HCV RNA < LLOQ target not detected at Weeks 4 and 12 of treatment) will continue treatment for a further 12 weeks with only Lambda/RBV to complete a total of 24 weeks of treatment.
- Cirrhotic and non-cirrhotic subjects not achieving eRVR will continue treatment for a further 36 weeks with only Lambda/RBV to complete a total of 48 weeks of treatment.

Follow-up Period:

Subjects will be followed-up, off-treatment for 48 weeks to assess SVR 24 or to evaluate treatment emergent resistance. Subjects who wish to initiate alternative anti-HCV treatment during post-treatment follow-up must discontinue the study, however, they must first complete a minimum of 4 weeks of follow-up before discontinuing the study.

Approved v4.0 930056810 4.0

Figure 1: Part A: Open Label Sentinel Cohort (N=25)

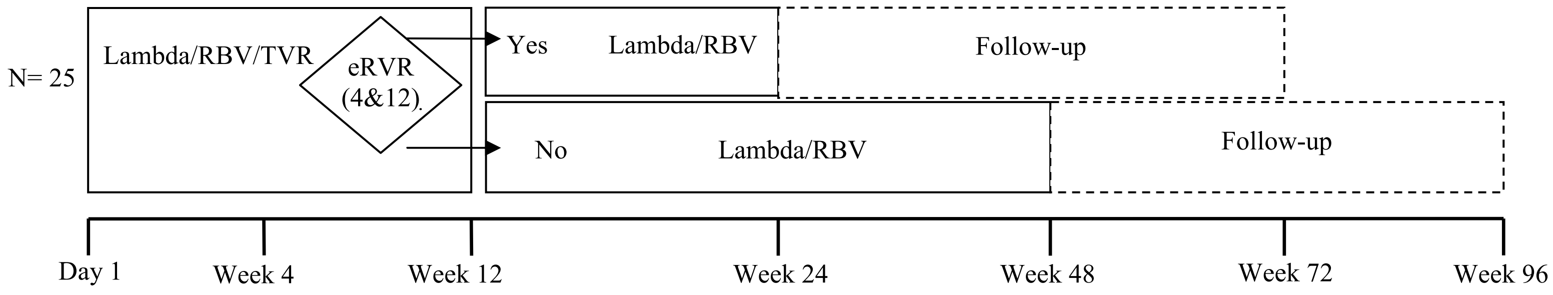

See futility criteria described in Section 3.1.3.

Approved v4.0 930056810 4.0

Transition to Part B will depend on whether results of Part A show an acceptable safety profile and meet go-no-go criteria (described below).

Go/No-Go Decision to Part B:

After all the subjects have completed 6 weeks of treatment, the discontinuation rate will be reviewed. If the rate of drug-related AEs leading to discontinuation ≤ 12%, then Part B may proceed parallel, while subjects continue on study in Part A.

An interim analysis of Part A will be conducted after all subjects have completed 12 weeks of treatment. The study will continue in Part B only if results in Part A meet the primary safety criteria (an observed rate of ≤ 24% of drug-related AEs leading to discontinuation).

Part B: Randomized, Controlled, Double-Blinded Cohort

Part B is a randomized, double-blind, active controlled phase of the study. Approximately 609 subjects with GT-1 chronic HCV infection will be randomized (2:1) to one of two treatment arms:

a. Lambda/RBV/TVR (n = 406)
b. alfa-2a/RBV/TVR (n = 203)

Part B will include subjects who are either treatment naive or who are relapsers to previous alfa/RBV treatment. Randomization will be stratified by prior relapse status, IL-28B CC and non-CC status, and viral GT-1a and -1b subtypes. Subjects with HCV GT-1b subtype will be capped at approximately 50%. Relapsers will be capped at approximately 20%. Subjects with compensated cirrhosis will be capped at approximately 10%.

Recent data on the risks of hepatic decompensation of an IFN-containing triple regimen with TVR is provided by the ANS CUPIC study. In this study, there were 292 compensated cirrhotic (Child Pugh A) nonresponders that were treated with alfa/RBV/TVR, and 33.1% had esophageal varices at baseline. In this interim analysis, infections were seen in 6.5% and new decompensation was seen in 2.0% of subjects treated with the TVR triple regimen. Compensated cirrhotic subjects classified as Child Pugh A may have clinically significant portal hypertension, putting them at higher intrinsic risk for decompensation events. Indicators of significant portal hypertension include hepatic venous pressure gradient (HVPG) ≥ 10 mg Hg, presence of esophageal or abdominal varices, any splenomegaly on imaging > 12 cm (diameter), and a Fibroscan score ≥ 21 kPa.

Compensated cirrhotic subjects without evidence of portal hypertension will be eligible for the study (see Section 3.3.2 - Exclusion criteria 2a and 2b). Data evaluating portal hypertension and other relevant clinical and laboratory data must be reviewed by the BMS Central Medical Monitor prior to randomization.

Treatment Period:

On Day 1, subjects will start treatment with Lambda/RBV/TVR or alfa-2a/RBV/TVR.

- TVR will only be administered for the first 12 weeks of treatment.
- Noncirrhotic subjects achieving eRVR (HCV RNA < LLOQ target not detected at Weeks 4 and 12 of treatment) will continue treatment for a further 12 weeks with only Lambda or Alfa-2a/RBV to complete a total of 24 weeks of treatment.
- Noncirrhotic subjects not achieving eRVR will continue treatment for a further 36 weeks with only Lambda or Alfa-2a/RBV to complete a total of 48 weeks of treatment.
- All cirrhotic subjects will continue treatment for a further 36 weeks with alfa-2a/RBV or Lambda/RBV to complete a total of 48 weeks of treatment.

Follow-up Period:

Subjects will be followed-up, off-treatment for 48 weeks to assess SVR 24 or to evaluate treatment emergent resistance. Subjects who wish to initiate alternative anti-HCV treatment during post-treatment follow-up must

Approved v 4.0 930056810 4.0

discontinue the study, however, they must first complete a minimum of 4 weeks of follow-up before discontinuing the study.

Figure 2: Part B: Randomized, IFN-blinded, Non-inferiority Design (N=609)

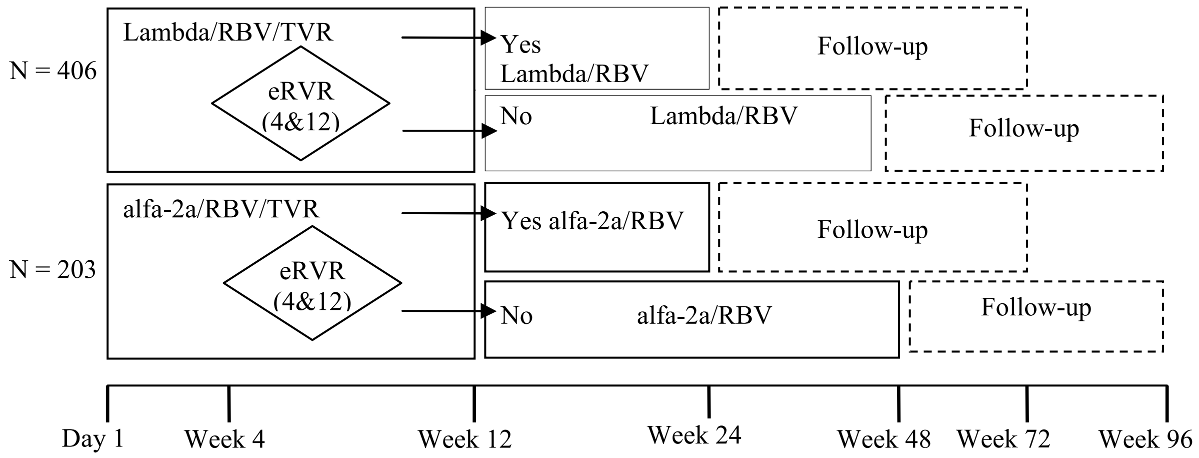

See futility criteria described in Section 3.1.3.

Futility Criteria for Parts A and B

Discontinue all drugs if:

1. Week 4 or 12: HCV RNA > 1000 IU/mL
2. Week 24: confirmed HCV RNA detected
3. Virologic Breakthrough:

   a. Confirmed* > 1 $\log_{10}$ increase in HCV RNA over nadir

   OR

   b. Confirmed* HCV RNA ≥ LLOQ after previously having an HCV RNA level of < LLOQ (target detected or target not detected) while on treatment.

   *Measurements should ideally be confirmed within 2 weeks of the original result

The Week 4, 12 and 24 futility criteria is based on the futility criteria in the local Telaprevir labels. The breakthrough criteria identify subjects who may potentially have developed resistance to the treatment regimen and as such may not benefit from further treatment.

Study Population: Subjects with GT-1 chronic HCV infection and compensated liver disease who are treatment naïve or who relapsed on prior treatment with Alfa-2a/RBV.

Key Inclusion Criteria:

- Signed Written Informed Consent
  - Freely given informed consent must be obtained from subjects prior to clinical trial participation, including informed consent for any screening procedures conducted to establish subject eligibility for the study.

Approved v4.0 930056810 4.0

- Target Population
  - Naïve to prior anti-HCV therapy (IFN and direct acting antiviral [DAA] based), **OR**
  - Relapsers, defined as subjects who had HCV RNA < LLOQ, target not detected at end of treatment with a prior alfa/RBV regimen and a HCV RNA ≥ LLOQ during the follow-up period after treatment. Relapsers previously treated with Lambda will not be excluded. The relapser population will be capped at approximately 20%.
  - Subjects with GT-1a or -1b subtypes chronic HCV infection (the naive population with GT-1b will be capped at approximately 50%). Genotype testing results should be available at the time of enrollment. Mixed genotypes are not allowed.
  - Compensated cirrhotic and non-cirrhotic subjects chronically infected with HCV as documented by either:
    - ♦ Positive anti-HCV antibody, HCV RNA or a positive HCV genotype test at least 6 months prior to screening; or
    - ♦ Liver biopsy consistent with chronic HCV infection (evidence of fibrosis and/or inflammation)

    The cirrhotic population will be capped at approximately 10%.
  - HCV RNA viral load by PCR ≥ 100,000 IU/mL at screening
  - Subjects must have one of the following assessments to evaluate for cirrhosis based on local country/institution requirements:

    i) Liver biopsy:

    For eligible non-cirrhotic subjects, liver biopsy results consistent with chronic HCV (evidence of fibrosis and/or inflammation) must be obtained within 3 years prior to enrollment. For eligible compensated cirrhotic subjects, biopsy documenting cirrhosis can be from any time period prior to randomization. Non cirrhotic subjects must have a documented liver biopsy with an Ishak score ≤ 4 or Metavir fibrosis score ≤ 3, or equivalent (Batts-Ludwig, etc). Compensated cirrhotic subjects must have a documented liver biopsy with an Ishak fibrosis score of ≥ 5 or a Metavir fibrosis score of 4, or equivalent (Batts-Ludwig, etc).

    ii) Fibroscan®:

    For countries where liver biopsy is not required prior to treatment and where non-invasive imaging tests (Fibroscan® ultrasound) are standard of care for staging of liver disease, a Fibroscan® done prior to screening is acceptable if it was performed within one year of screening (≥ 14.6 kPa should be considered consistent with cirrhosis). If the prior Fibroscan® was not performed within one year of screening, a new Fibroscan® is required before study drug dosing. If a subject has both liver biopsy and Fibroscan®, the results of the liver biopsy take precedence over those of the Fibroscan®.

    iii) All cirrhotic subjects must have at least one investigation to assess for the presence of portal hypertension (abdominal ultrasound scan or Fibroscan at a minimum). If a subject has had additional evaluation for liver disease, then all prior observations must meet the following criteria in order for the subject to be eligible for treatment: a hepatic venous pressure gradient < 10 mg Hg, no history or current findings/evidence of ascites or varices, any splenomegaly on imaging must be < 12 cm (diameter), and Fibroscan score must be < 21 kPa (see Key Exclusion Criteria). These data must be reviewed by the BMS Central Medical Monitor prior to randomization.

Key Exclusion Criteria

- Target Disease Exceptions
  - Infected with HCV other than GT-1a and GT-1b subtypes
  - Positive HBsAg or HIV-1/HIV-2 antibody at screening

Approved v4.0 930056810 4.0

- Evidence of a medical condition associated with chronic liver disease other than HCV (such as but not limited to: hemochromatosis, autoimmune hepatitis, alcoholic liver disease, biliary disease, nonalcoholic hepatic steatosis, and toxin exposure)
- Previous exposure to DAA treatment or HCV experimental agents
- Previous exposure to IFN based treatment (excluding relapsers)
- Current evidence of or history of portal hypertension, know hepatic venous pressure gradient (HVPG) ≥ 10 mm Hg, presence of esophageal or abdominal varices, any splenomegaly on imaging > 12 cm (diameter), hepatic encephalopathy, or ascites requiring diuretics or paracentesis or evidence of any of these findings on physical examination performed at screening.
- Fibroscan scores ≥ 21 kPa if Fibroscan is used to assess staging of liver fibrosis

Study Assessments:

Primary Endpoint:

Part A:

Proportion of subjects treated with Lambda/RBV/TVR who achieve eRVR, defined as HCV RNA < LLOQ target not detected at Weeks 4 and 12 of treatment, and the proportion of subjects treated with Lambda/RBV/TVR who develop safety related events (as measured by the frequency of deaths, SAEs, drug related AEs, dose reductions and discontinuations due to AEs) through end of treatment (maximum of 48 weeks) of Lambda/RBV/TVR in a sentinel cohort of subjects with GT-1 chronic HCV infection.

Part B:

Proportion of subjects who achieve efficacy as measured by SVR12, defined as HCV RNA < LLOQ (target detected or target not detected), at Week 12 of post-treatment follow-up of Lambda/RBV/TVR and alfa-2a/RBV/TVR in subjects who are treatment naive or who relapsed on prior alfa/RBV therapy.

Secondary Endpoints:

Part A

- Proportion of subjects who achieve efficacy as measured by SVR12, defined as HCV RNA < LLOQ (target detected or target not detected), at Week 12 of post treatment follow-up
- Proportion of subjects who achieve efficacy as measured by SVR24, defined as HCV RNA < LLOQ (target detected or target not detected), at Week 24 of post-treatment follow-up

Part B

- Proportion of subjects who achieve efficacy as measured by SVR12, defined as HCV RNA < LLOQ (target detected or target not detected), at Week 12 of post-treatment follow-up in treatment-naive subjects
- Cytopenic abnormalities (anemia is defined by Hg < 10 g/dL, neutropenia as defined by ANC < 750 mm^3, thrombocytopenia as defined by platelets < 50,000 mm^3) through end of treatment (maximum of 48 weeks)
- Proportion of subjects who achieve efficacy as measured by eRVR, defined as HCV RNA < LLOQ at Weeks 4 and 12 of treatment
- Flu-like symptoms (as defined by pyrexia or chills or pain) through end of treatment (maximum of 48 weeks)
- Musculoskeletal symptoms (as defined by arthralgia or myalgia or back pain) through end of treatment (maximum of 48 weeks)
- Proportion of subjects who achieve efficacy as measured by SVR24, defined as HCV RNA < LLOQ (target detected or not detected) at Week 24 of post-treatment follow-up.

Approved v4.0 930056810 4.0

Other Secondary Endpoints

- Proportion of subjects who achieve efficacy as measured by virologic response at Week 48 of post-treatment follow-up.
- Safety as measured by the frequency of deaths, SAEs, drug-related AEs, dose reductions, and discontinuations due to AEs, and treatment-emergent laboratory abnormalities through follow-up Week 4
- Constitutional symptoms (fatigue or asthenia) through end of treatment (maximum 48 weeks)
- Neurologic symptoms (headache or dizziness) through end of treatment (maximum 48 weeks)
- Psychiatric symptoms (depression or irritability or insomnia) through end of treatment (maximum 48 weeks)
- Occurrence of rash through end of treatment (maximum 48 weeks)

Exploratory Endpoints

- Pharmacokinetics (PK) of Lambda/TVR compared to alfa-2a/TVR
- Exposure and antiviral response relationship
- Viral resistance to Lambda/TVR
- Biomarkers of host immune response (potentially including serum protein markers, gene expression in whole blood)
- Immunogenicity of Lambda
- SNPs in *IL28B* (including rs12979860) or *ENT1* and clinical response relationship
- Patient Reported Outcomes (PRO)
    - 'Flu-Like' Symptoms evaluated using the Hepatitis Physical Symptom Severity Diary
    - Fatigue evaluated using the Fatigue Severity Scale
    - Depression evaluated using the Patient Health Questionnaire (PHQ-9)
    - Health-related quality of life evaluated using the EQ-5D questionnaire

Statistical Methods:

Part A:

Data from Part A will be analyzed separately from Part B. Only descriptive statistics will be provided for Part A.

Part B

A two-stage evaluation of the efficacy of Lambda compared to alfa-2a is planned. In the first stage, the non-inferiority of Lambda to alfa-2a will be tested. Sample size calculations for the non-inferiority testing assume the same response rate for SVR12 in both treatment arms. Provided non-inferiority is established, a second stage test will be conducted to demonstrate superiority. Because the test for superiority will be conducted only if the test for non-inferiority is successful, significance levels will not be adjusted for the first stage of testing.

Non-inferiority:

With 406 Lambda treated subjects and 203 alfa-2a treated subjects, there is 95% power to demonstrate non-inferiority of Lambda to alfa-2a, for the proportion of subjects with SVR12 at Week 12 post-treatment follow-up, assuming:

Approved v4.0 930056810 4.0

A response rate of 79% for both alfa-2a and Lambda

A -12% boundary for comparison with the lower limit of the two-sided 95% confidence interval for the treatment difference (λ - α)

Superiority:

With 406 Lambda treated subjects and 203 alfa-2a treated subjects, there is > 90% power for testing superiority of Lambda compared to alfa-2a assuming:

A 79% response rate for alfa-2a and an 89% rate for Lambda

A type I error of 0.05 (two-sided)

Subgroup analysis on the naïves:

Assuming 80% of the subjects are naïve, there will be 324 Lambda treated and 162 alfa-2a treated naïve subjects. This sample size will provide 90% power for the non-inferiority comparison and 82% power for the superiority comparison between two treatment groups in the naïve subjects, based on the same assumptions as above.

The primary efficacy endpoint will be analyzed using modified intent to treat (ITT):

Modified ITT: The numerator is based on subjects meeting the response criteria. The denominator is based on all treated subjects.

Approved v4.0 930056810 4.0

TABLE OF CONTENTS

TITLE PAGE 1
DOCUMENT HISTORY 3
SYNOPSIS 6
TABLE OF CONTENTS 16
1 INTRODUCTION AND STUDY RATIONALE 20
1.1 Study Rationale 23
1.2 Research Hypothesis 24
1.3 Objectives 24
1.3.1 Primary Objectives 25
1.3.2 Secondary Objectives 25
1.3.3 Other Secondary Objectives 25
1.3.4 Exploratory Objectives 26
1.4 Product Development Background 26
1.4.1 Background of Lambda 26
1.4.2 Background of Telaprevir 27
1.4.3 Non-Clinical Safety Studies 28
1.4.4 Clinical Experience with Lambda 28
1.4.5 Clinical Experience with Telaprevir 31
1.5 Overall Risk/Benefit Assessment 33
1.5.1 Lambda 33
1.5.2 Telaprevir 35
2 ETHICAL CONSIDERATIONS 39
2.1 Good Clinical Practice 39
2.2 Institutional Review Board/Independent Ethics Committee 39
2.3 Informed Consent 39
3 INVESTIGATIONAL PLAN 40
3.1 Study Design and Duration 40
3.1.1 Part A: Open Label Sentinel Cohort 40
3.1.2 Part B: Randomized, Controlled, Double-Blinded Cohort 43
3.1.3 Futility Criteria for Parts A and B 45
3.2 Post Study Access to Therapy 45
3.3 Study Population 45
3.3.1 Inclusion Criteria 46
3.3.2 Exclusion Criteria 49
3.3.3 Women of Childbearing Potential 52
3.4 Concomitant Treatments 53
3.4.1 Prohibited and/or Restricted Treatments 53
3.4.1.1 Prohibited/Restricted during Dosing of TVR 53
3.4.2 Other Restrictions and Precautions 54
3.5 Discontinuation of Subjects from Treatment 54
3.5.1 Laboratory/Clinical Criteria for Treatment Discontinuation 55
4 TREATMENTS 55
4.1 Study Treatments 56
4.1.1 Investigational Product 58

Approved v4.0 930056810 4.0

4.1.2 Non-investigational Product 58
4.1.3 Handling and Dispensing 58
4.2 Method of Assigning Subject Identification 59
4.3 Selection and Timing of Dose for Each Subject 60
4.3.1 Dose Modifications 61
4.3.1.1 Lambda and alfa-2a Dose Modifications 62
4.3.1.2 Depression Monitoring and Dose Modifications 65
4.3.1.3 Ribavirin Dose Modifications 66
4.3.1.4 Telaprevir 67
4.3.2 Dose Interruptions 67
4.4 Blinding/Unblinding 68
4.5 Treatment Compliance 69
4.6 Destruction and Return of Study Drug 69
4.6.1 Destruction of Study Drug 69
4.6.2 Return of Study Drug 70
5 STUDY ASSESSMENTS AND PROCEDURES 71
5.1 Flow Chart/Time and Events Schedule 71
5.2 Study Materials 80
5.3 Safety Assessments 80
5.3.1 Laboratory Assessments 80
5.3.2 Adverse Events Assessments 83
5.3.3 Electrocardiogram 83
5.3.4 Vital Signs and Physical Examinations 83
5.3.5 Anemia Management for TVR Treated Patients 83
5.3.6 Rash Management 84
5.3.6.1 Rash or Rash-Like Events of Special Interest 84
5.3.6.2 Rash Assessment 84
5.3.6.3 Rash Treatment 85
5.3.6.4 Rash Management for TVR 85
5.3.7 Serious Skin Reactions 87
5.4 Efficacy Assessments 87
5.4.1 Primary Efficacy Assessment 87
5.4.2 Secondary Efficacy Assessments 87
5.5 Pharmacokinetic Assessments 87
5.6 Biomarker Assessments 89
5.6.1 Pharmacodynamic Assessments 89
5.6.2 Pharmacogenomic/Pharmacogenetic Assessments 89
5.7 Outcomes Research Assessments 90
5.7.1 Patient Health Questionnaire (PHQ-9) 90
5.7.2 Columbia-Suicide Severity Rating Scale (C-SSRS) 90
5.7.3 'Flu-like symptom' index, as an exploratory endpoint, using the 'HPSS-D' diary 90
5.7.4 Fatigue Severity Scale 91
5.7.5 EQ-5D (to measure patient utilities for Health Authority submissions) .. 91
5.8 Other Assessments 91
5.8.1 Resistance Monitoring 91

Approved v 4.0 930056810 4.0

5.8.2 Immunogenicity Assessment 93
6 ADVERSE EVENTS 93
6.1 Serious Adverse Events 94
6.1.1 Serious Adverse Event Collection and Reporting 95
6.2 Nonserious Adverse Events 96
6.2.1 Nonserious Adverse Event Collection and Reporting 96
6.3 Laboratory Test Result Abnormalities 96
6.4 Pregnancy 96
6.5 Overdose 97
6.6 Potential Drug Induced Liver Injury (DILI) 97
6.7 Other Safety Considerations 98
7 DATA MONITORING COMMITTEE AND OTHER EXTERNAL COMMITTEES 98
8 STATISTICAL CONSIDERATIONS 99
8.1 Sample Size Determination 99
8.2 Populations for Analyses 100
8.3 Endpoints 100
8.3.1 Primary Endpoint(s) 100
8.3.2 Secondary Endpoint(s) 100
8.3.3 Other Secondary Endpoints 101
8.3.4 Exploratory Endpoint(s) 101
8.4 Analyses 102
8.4.1 Demographics and Baseline Characteristics 102
8.4.2 Efficacy Analyses 102
8.4.3 Safety Analyses 103
8.4.4 Pharmacokinetic Analyses 104
8.4.5 Biomarker Analyses 104
8.4.6 Outcomes Research Analyses 105
8.4.7 Other Analyses 105
8.5 Interim Analyses 106
9 STUDY MANAGEMENT 106
9.1 Compliance 106
9.1.1 Compliance with the Protocol and Protocol Revisions 106
9.1.2 Monitoring 107
9.1.3 Investigational Site Training 107
9.2 Records 107
9.2.1 Records Retention 107
9.2.2 Study Drug Records 107
9.2.3 Case Report Forms 108
9.3 Clinical Study Report and Publications 109
10 GLOSSARY OF TERMS 110
11 LIST OF ABBREVIATIONS 111
12 REFERENCES 115

Approved v4.0 930056810 4.0

APPENDIX 1 DSM IV: DIAGNOSTIC CRITERIA FOR DRUG AND ALCOHOL ABUSE 119
APPENDIX 2 DIVISION OF AIDS TABLE FOR GRADING THE SEVERITY OF ADULT AND PEDIATRIC ADVERSE EVENTS PUBLISH DATE: DECEMBER, 2004.. 120

Approved v4.0 930056810 4.0

1 INTRODUCTION AND STUDY RATIONALE

Hepatitis C Infection

Hepatitis C virus (HCV) is found in almost every region in the world. Approximately 3% of the world's population, 170 - 180 million people, are infected with HCV.[1,2,3,4] It is estimated that there are 4 million chronic HCV carriers in the United States of America (USA)[5] and 5 million in Western Europe, with higher prevalence in Eastern Europe.[4] Chronic HCV infection accounts for 20% of cases of acute hepatitis[6] and 70% of cases of chronic liver disease.[6,7] In industrialized countries, chronic HCV accounts for 40% of cases of end stage cirrhosis[6] ,60% of cases of hepatocellular carcinoma (HCC)[2,6,8] and 30% of liver transplantation.[6,9,10] Quality of life is also impaired for patients infected with the virus[11], even among those without cirrhosis.[12] Although the incidence of new infections has declined, the number of deaths may continue to increase 2- to 4-fold over the next 20 years due to prevalent cases with longstanding infection.[13,14,15] Currently, 10,000 to 12,000 deaths are attributed to HCV annually in the U.S. The morbidity and mortality associated with HCV are expected to rise, however, as the existing HCV-infected population ages[16].

HCV is a non-cytopathic ribonucleic acid (RNA) virus that preferentially replicates in hepatocytes. It is most commonly spread by direct contact with infected blood and blood products. HCV infection is often asymptomatic, making it very difficult to detect at an early stage. The virus has evolved mechanisms to evade immune elimination, thereby allowing it to persist in the liver in the majority of infected individuals. The virus is capable of mutating to escape surveillance[17] despite the production of neutralizing antibodies during the course of the infection. When the liver fails to clear the virus, the individual becomes a chronic carrier. As the initial acute infection is often asymptomatic, individuals usually present with symptoms of chronic HCV infection.

HCV is classified into at least 7 major genotypes (GT) that differ by about 30% in their nucleotide sequence. The genotypes (GT-1, -2, -3, -4, -5, -6, and -7) show differences with regard to their worldwide distribution, transmission and disease progression[18]. HCV GT-1 is primarily found in North and South America and in Australia. GT-2a is most common in Japan and China while GT-2b is the most common GT-2 in USA and Northern Europe. GT-2c is most common in Western and Southern Europe, Pakistan and India. In Japan, Taiwan and China, GT-1b and -2b are also found. While GT-3 is prevalent in some parts of the UK and USA, GT-3a is highly prevalent in Australia and South Asia. GT-4 is most common in Middle East and Northern and, central Africa. GT-4a is prevalent in Egypt. GT-4c is highly prevalent in Central Africa. GT-5 is prevalent only in South Africa. GT-6 is found mainly in Asia and Hong Kong. GT-6a is restricted to Hong Kong (where it accounts for one third of those infections), Macau and Vietnam. GT-7a and 7b are common in Thailand. GT-8a, -8b, and -9a are prevalent in Vietnam. Also the lesser known GT-10a and -11a are prevalent in Indonesia[19].

Approved v4.0 930056810 4.0

Treatment of Hepatitis C Genotype 1 Infection

Chronic HCV GT-1 is found primarily in North and South America and in Australia. About 70% of the patients in USA are infected with HCV GT-1.[19] GT-1a is also common in USA[20] and United Kingdom (UK).[19] Genotype 1b is mostly found in Europe and Asia and is common in Japan.[19] Unfortunately, studies show that infection with HCV GT-1 is more difficult to treat than infection with HCV GT-2 and -3.[16,20,21]

The main pre-treatment predictors of a favourable sustained virological response (SVR) are host genotype (in particular the CC genotype at the locus rs12979860, upstream of the IL28B gene, on chromosome 19; as opposed to the CT/TT genotype at this locus) , viral genotype and pre-treatment viral load[22,23]. Other reported baseline characteristics associated with a favourable response include the doses of peginterferon (1.5 µg/kg/week versus 0.5µg/kg/week) and RBV (> 10.6 mg6mg/kg), female gender, age less than 40 years, non-African American race, lower body weight (≤ 75 kg), the absence of insulin resistance, elevated ALT (three-fold higher than upper limit of normal) and the absence of bridging fibrosis or cirrhosis on liver biopsy.[22,23,24] In addition to pre-treatment indicators, changes in viral load at specific time points during treatment can be used to predict the likelihood of achieving SVR and, in some cases, to tailor the duration of therapy (response guided therapy-RGT). Patients who fail to achieve early virologic response [EVR; ≥ 2 log10 decrease in HCV RNA from baseline, at Weeks 12 of treatment or < HCV RNA < 25IU/ml (detected or not detected) at Week 12 of treatment] have a < 3% chance of achieving SVR. Achieving a rapid virological response (RVR-defined as HCV RNA negative at treatment week 4) is also highly predictive of obtaining an SVR independent of genotype and regardless of the treatment regimen.[25] Because of the rapid clearance of virus from serum, patients who achieve an RVR may be able to shorten the duration of treatment.[26]

Up until 2011, for patients with chronic HCV GT-1, the standard of care treatment was 48 weeks of treatment using pegylated interferon alfa (alfa), in combination with ribavirin (RBV).[21] In this population, this treatment regimen had sustained virological/ response rates, 24 weeks after the end of treatment (SVR24), of between 40% - 50%.[21] Recently however, in the US and in many countries in Europe, two protease inhibitor class of directly-acting antivirals (DAAs), Telaprevir (TVR) and Boceprevir (BOC)[27], have been approved to be used, in combination with alfa and RBV, for the treatment of naive and experienced patients with chronic HCV GT-1. Response guided treatment using TVR or BOC, each in combination with alfa/RBV, is able to reduce treatment duration, in patients who meet early virological response criteria, from 48 weeks to 24 (TVR treated subjects) or 28 weeks (BOC treated subjects) and, improve SVR24 rates to between 75% and 63% respectively, for TVR[47] and BOC,[28] treated subjects.

Toxicities Associated with Current Therapy for Chronic HCV Genotype 1 Infection

In patients infected with chronic HCV GT-1, despite the improvement in SVR rates and the option of short treatment duration, when a DAA is used in combination with alfa and RBV, the use of these DAA, TVR and BOC, are related adverse events (AE). The most common AE

Approved v4.0 930056810 4.0

associated with the use of TVR are rash, anemia, pruritus, nausea and diarrhea while treatment with BOC is commonly associated with anemia and dysgeusia.[21]

Furthermore, the use of alfa/RBV in itself is also associated with AEs that prevent initiation of therapy for a number of HCV infected subjects. The AEs commonly associated with the use of alfa are flu-like symptoms, hematological and hepatic abnormalities and, neuropsychiatric disorders.[45] Other AEs include gastrointestinal, dermatological, autoimmune, cardiac[29] as well as pulmonary and ophthalmologic.[30] The use of RBV most notably leads to hemolytic anemia, which in combination with the myelosuppressive effects of alfa can be a significant clinical problem.[31,32]

The toxicities associated with the use of TVR, BOC, alfa and RBV may lead to avoidance of therapy, delays in starting therapy or discontinuation of treatment.[33,34] Furthermore, the toxicities associated with the use of alfa or RBV may lead to dose reductions and early discontinuation of treatment.[29,35] These factors, including poor adherence to treatment, may decrease the likelihood of achieving SVR. Adherence to therapy (defined as receiving ≥ 80% of the prescribed alfa dose and ≥ 80% of the RBV dose for the duration of therapy) has been associated with higher SVR rates in chronic HCV GT-1 infected patients.[36]

Need for New Treatments for Chronic HCV

Despite the advantages of alfa/RBV based DAA treatment regimens, using the currently approved DAAs, TVR or BOC[21], these regimens are not without their toxicities and concerns about resistance.[18] These regimens also do not address many of the safety and tolerability issues associated with the use of alfa and RBV and not all patients are eligible for a shortened duration of treatment.[34] As such, there remains a need for the development of safer and simpler treatments regimens that may also efficaciously further shorten treatment duration in patients with GT-1 chronic HCV infection.

An alternative approach to improving treatment outcomes in subjects with GT-1 chronic HCV infection is to develop newer interferon molecules that maybe combined with newer DAAs, to improve the tolerability and adherence to interferon-based treatments regimens. This approach may limit interferon related dose reductions and treatment discontinuations and, may potentially enable the development of efficacious treatment regimens that may shorten treatment durations.

Key Efficacy Terminology:

The Roche COBAS® TaqMan HCV Test v.2.0 (lower limit of quantitation [LLOQ] = 25 IU/mL; limit of detection ~ 10 IU/mL for HCV GT-1 subtype) will be used to measure HCV RNA levels. When the titer result is target not detected, the results will be reported by our laboratory as, “HCV RNA not detected.” When the titer result is < 25 IU/Ml, the results will be reported as “HCV RNA < 25 IU/mL, target detected.”

Approved v4.0 930056810 4.0

Response definitions for this study are:

- RVR (Rapid Virologic Response): HCV RNA < Lower Limit of Quantification (LLOQ) target not detected at Week 4 of treatment
- eRVR (Extended Rapid Virologic Response): HCV RNA < LLOQ target not detected at Weeks 4 and 12 of treatment
- EVR (Early Virologic Response): ≥ 2 $\log_{10}$ decrease in HCV RNA from baseline, at Weeks 12 of treatment, or < LLOQ (target detected or not detected) at Weeks 12 of treatment
  - cEVR (Complete Early Virologic Response): HCV RNA < LLOQ target not detected at Week 12 of treatment
- Virologic Breakthrough:
  a. Confirmed > 1 $\log_{10}$ increase in HCV RNA over nadir
     OR
  b. Confirmed HCV RNA ≥ LLOQ after previously having an HCV RNA level of < LLOQ (target detected or target not detected) while on treatment

  Measurements should be confirmed within 2 weeks of the original result.
  - SVR4 (Sustained Virologic Response at post-treatment follow-up week 4): HCV RNA < LLOQ (target detected or target not detected) at Week 4 of post -treatment follow-up
  - SVR12 (Sustained Virologic Response at post-treatment follow-up week 12): HCV RNA < LLOQ (target detected or target not detected) at Week 12 of post -treatment follow-up
  - SVR24 (Sustained Virologic Response at post-treatment follow-up week 24): HCV RNA < LLOQ (target detected or target not detected) at Week 24 of post -treatment follow-up
  - Relapse: HCV RNA < LLOQ, target not detected at the end of treatment followed by HCV RNA ≥ LLOQ at any post-treatment follow-up visit

1.1 Study Rationale

The rationale for conducting this study is to be able to offer subjects infected with GT- 1 chronic HCV infection an opportunity to be treated and cured with a potentially more tolerable interferon, Lambda, in combination with TVR and RBV, over a shorter treatment duration.

Rationale to Support Drug Combination

The rationale to support the use of Lambda in combination with RBV (Lambda/RBV) in subjects infected with chronic HCV GT-1 is based on data from part B of the phase 2b EMERGE study (AI452004). Details of the EMERGE study can be found in the Investigators Brochure (IB). A summary of the clinically relevant data from the Phase 2b study is discussed below.

AI452020 is the first study to evaluate the clinical efficacy and safety of Lambda/RBV in combination with TVR in a human population. Preclinical data indicates that Lambda does not

Approved v4.0 930056810 4.0

inhibit or induce any of the CYP450 isoforms tested in human liver microsomes and hepatocytes and, does not alter the total CYP450 content or the individual enzyme activities in the livers of treated cynomolgus monkeys[37]. It is therefore anticipated that the likelihood of a drug interaction between Lambda and TVR, via CYP enzymes, is low. As such, it is anticipated that a treatment regimen consisting of Lambda/RBV/TVR may have the potential to result in efficacy rates that are comparable to a regimen consisting of alfa-2a/RBV/TVR and TVR, but with an improved safety and tolerability profile.

Rationale to Support Dose Selection:

Lambda

This phase 3 study will use the 180 μg dose of Lambda. The selection of the 180 μg dose was based on the Week 12 efficacy and safety data of 4 different doses of Lambda (80 μg, 120 μg, 180 μg, and 240 μg) evaluated in the Phase 2a of the EMERGE study, and on PK, dynamic viral and probabilistic modeling of those data.

Since 80 μg was thought to have minimal activity in Phase 2a, 120 μg, 180 μg and 240 μg doses were selected for further evaluation in the Phase 2b EMERGE study. Efficacy results from the Week-12 interim analysis from Phase 2b indicated that the RVR rates were higher at the 180 μg and 240 μg Lambda dose groups compared to the 120 μg dose group, with similar cEVR rates observed across the 120 μg, 180 μg and 240 μg Lambda dose groups. In addition, fewer liver-related laboratory abnormalities and dose reductions were observed at the 180 μg dose compared to the 240 μg dose. Based on the integrated assessment, the 180 μg dose is considered to have a favorable benefit-risk profile and is the recommended dose for the Phase 3 studies. Furthermore, based on the high exposure overlap seen between Lambda doses, the 180 μg dose allows for multiple step down doses (120 μg and 80 μg) should they be necessary for individual safety/tolerability concerns.

In the phase 2b part of EMERGE the 180 μg dose of Lambda in GT-2 and -3 subjects was shown to have a numerically superior SVR24 and also a more favorable overall safety profile, with low rates of dose reductions.

1.2 Research Hypothesis

Treatment with a regimen of Lambda, in combination with RBV and TVR (Lambda/RBV/TVR), will result in comparable efficacy and better tolerability than treatment with alfa-2a/RBV/TVR in treatment-naïve subjects and relapsers with GT-1-chronic (HCV) infection.

1.3 Objectives

This study has two parts, Part A and Part B. Part A is a single arm open label sentinel cohort and Part B is in a randomized controlled, double-blinded, cohort.

Approved v4.0 930056810 4.0

1.3.1 Primary Objectives

Part A: Single Arm, Open Label Sentinel Cohort

To evaluate efficacy as measured by eRVR and safety (as measured by the frequency of deaths, SAEs, drug related AEs, dose reductions and discontinuations due to AEs) of Lambda/RBV/TVR in a sentinel cohort of subjects with GT-1 chronic HCV infection who are treatment-naïve or who relapsed on a prior alfa/RBV treatment regimen.

Part B: Randomized Controlled, Double Blinded Cohort

To evaluate efficacy as measured by SVR12 of Lambda/RBV/TVR compared to alfa-2a/RBV/TVR in subjects with GT-1 chronic HCV infection who are treatment naive or who relapsed on a prior alfa/RBV treatment regimen.

1.3.2 Secondary Objectives

Part A:

- Evaluation of efficacy as measured by proportion of subjects with eRVR who achieve SVR12
- Evaluation of efficacy as measured by proportion of subjects who achieve SVR24

Part B:

- Evaluation of efficacy as measured by proportion of subjects who achieve SVR12 in treatment-naïve subjects
- Evaluation of safety of Lambda/RBV/TVR compared to alfa-2a/RBV/TVR, as measured by the reduction in treatment-emergent cytopenic abnormalities (anemia is defined by Hb < 10 g/dL, neutropenia as defined by ANC < 750 mm^3, thrombocytopenia as defined by platelets < 50,000 mm^3)
- Evaluation of efficacy as measured by proportion of subjects who achieve eRVR
- Evaluation of safety as measured by the following on-treatment interferon-associated symptoms following treatment with Lambda/RBV/TVR compared to alfa-2a/RBV/TVR:
  - Flu-like symptoms (as defined by pyrexia or chills or pain)
  - Musculoskeletal symptoms (as defined by arthralgia or myalgia or back pain)
- Evaluation of efficacy as measured by proportion of subjects who achieve SVR24

1.3.3 Other Secondary Objectives

- Evaluation of efficacy as measured by proportion of subjects who achieve virologic response at Week 48 of post-treatment follow-up

Approved v4.0 930056810 4.0

- Evaluation of safety of Lambda/RBV/TVR compared to alfa-2a/RBV/TVR (as measured by the frequency of deaths, SAEs, drug related AEs, dose reductions and discontinuations due to AEs) and treatment emergent laboratory abnormalities
- Evaluation of safety as measured by the following on-treatment interferon-associated symptoms following treatment with Lambda/RBV/TVR compared to alfa-2a/RBV/TVR:
  - Constitutional symptoms (fatigue or asthenia)
  - Neurologic symptoms (headache or dizziness)
  - Psychiatric symptoms (depression or irritability or insomnia)
- Evaluation of safety as measured by the occurrence of rash following treatment with Lambda/RBV/TVR compared to alfa-2a/RBV/TVR

1.3.4 Exploratory Objectives

- Evaluation of the pharmacokinetics of Lambda/TVR compared to alfa-2a/TVR
- Investigation of the relationship between Lambda and TVR exposure and antiviral responses
- Evaluation of viral resistance to Lambda/TVR
- Evaluation of biomarkers of host immune response (potentially including serum protein markers, gene expression in whole blood)
- Evaluation of the immunogenicity of Lambda in combination with TVR
- Determination of the association between SNPs in *IL28B* (including rs12979860) or *ENT1* and clinical responses to Lambda/RBV/TVR or alfa-2a/RBV/TVR
- Evaluation of the following Patient Reported Outcomes (PROs):
  - 'Flu-Like' Symptoms evaluated using the Hepatitis Physical Symptom Diary
  - Fatigue evaluated using the Fatigue Severity Scale
  - Depression evaluated using the Patient Health Questionnaire (PHQ)-9
  - Health-related quality of life evaluated using the EQ-5D questionnaire

1.4 Product Development Background

1.4.1 Background of Lambda

Lambda is a conjugate of a recombinant version of human IL-29 (rIL-29). It has a 20kDa linear polyethylene glycol (peg) chain, produced by reductive alkylation, to extend its half life in vivo.

Approved v4.0 930056810 4.0

Lambda Mechanism of Action:

Lambda binds to and signals through a unique cell-surface heterodimeric receptor (IL-29) composed of 2 chains: IL-28Rα, which is unique to this receptor complex, and IL-10Rβ, which is shared among several Class II cytokine receptors.[31,32,33] Binding of Lambda to its receptor stimulates intracellular phosphorylation of signal transducer and activator of transcription (STAT) proteins and, the induction of interferon-stimulated genes (ISGs), including protein kinase R (PkR), Myxovirus resistance (Mx), 2'5'-oligoadenylate synthetase (OAS), and β2-microglobulin (B2M). In a recent analysis of data from EMERGE phase 2a[38] it was observed that while Lambda causes a weaker expression of ISG compared to alfa-2a, the level of ISG induction in blood was not associated with differences in virologic response rates (RVR and cEVR) between Lambda and alfa-2a.

Based on levels of RNA expression, the IL-29 receptor is expressed in fewer cell types than the IFN-α receptor. For example, while the interferon (IFN)-α receptor is expressed in liver fibroblasts and hepatocytes, the IL-29 receptor is expressed only in hepatocytes. Similarly, RNA expression of the IL-29 receptor is low to absent in all circulating peripheral blood leukocytes (PBL) except B cells, whereas IFN-α receptor RNA is expressed at high levels in all PBLs, including B cells, T cells, natural killer (NK) cells, neutrophils, and monocytes. The more limited expression of the IL-29 receptor suggests that systemic administration of IL-29 may be associated with fewer adverse effects than systemic administration of Type I interferons such as IFN-α.

1.4.2 Background of Telaprevir

Mechanism of Action

TVR is an inhibitor of the HCV NS3/4A serine protease, necessary for the proteolytic cleavage of the HCV encoded polyprotein into mature forms of the NS4A, NS4B, NS5A, and NS5B proteins and essential for viral replication. In a biochemical assay, TVR inhibited the proteolytic activity of the recombinant HCV NS3 protease domain with an IC_{50} value of 10 nM.[49]

Antiviral Activity in Cell Culture

In an HCV subtype 1b replicon assay, the TVR EC_{50} value against wild-type HCV was 354 nM in a 2-day cell culture assay, and in a subtype 1a infectious virus assay, the EC_{50} value was 280 nM in a 5-day cell culture assay. In biochemical enzymatic assays, the median IC_{50} values of TVR against GT-2, -3a, and -4a were 16 nM (range 6 - 32 nM; n = 5), 40 nM (range 39 - 88 nM; n = 5), and 130 nM (n = 1), respectively, compared to a median IC_{50} value of 20 nM (range 16 - 23; n = 2) for GT-1a and 20 nM for GT-1b (range 13 - 33; n = 4). The presence of 40% human serum reduced the anti-HCV activity of TVR by approximately 10-fold. Evaluation of TVR in combination with peg-interferon alfa or ribavirin showed no evidence of antagonism in reducing HCV RNA levels in HCV replicon cells.

Approved v4.0 930056810 4.0

1.4.3 Non-Clinical Safety Studies

Data from the Lambda non-clinical studies can be found in the Investigator Brochure. The key TVR non-clinical data can be found in the local TVR package insert.

1.4.4 Clinical Experience with Lambda

AI452004 Study (EMERGE):

Part B of the EMERGE phase 2b study (AI452004) is a randomized control, double blinded study which evaluated the safety and efficacy of Lambda (180 μg)/RBV in comparison to alfa-2a/RBV.

Efficacy:

In part B of the phase 2b EMERGE study, for subject with chronic HCV GT-1/-4, the SVR12 rates in the Lambda (180 μg) and alfa-2a treatment groups were 38.2% and 38.8%, respectively. A summary of the EMERGE GT-1/-4 efficacy data is provided in Table 1.4.4A.

Table 1.4.4A: Part B EMERGE GT-1/-4 Efficacy Data

**Virologic Response**	**Alfa-2a/RBV (N = 103)**	**Lambda(180 μg)/RBV (N = 102)**
RVR		
Undetectable, n (%)	6 (5.8)	15 (14.7)
< LOQ, n/N (%)	13 (12.6)	24 (23.5)
cEVR		
Undetectable, n (%)	38 (36.9)	57 (55.9)
< LOQ, n (%)	56 (54.4)	74 (72.5)
EVR		
Undetectable n (%)	84 (81.6)	82 (80.4)
ETVR		
Undetectable n (%)	58 (56.3)	59 (57.8)
< LOQ at EOT, n%	59 (57.3)	60 (58.8)
SVR4		
Undetectable, n (%)	46 (44.7)	43 (42.2)
< LOQ 4 weeks post EOT	47 (45.6)	43 (42.2)
SVR12		
Undetectable, n (%)	40 (38.8)	39 (38.2)
95% CI	(29.4, 48.9)	(28.8, 48.4)

Approved v4.0 930056810 4.0

**Table 1.4.4A: Part B EMERGE GT-1/-4 Efficacy Data**		
**Virologic Response**	**Alfa-2a/RBV (N = 103)**	**Lambda(180 µg)/RBV (N = 102)**
P-value	-	0.9297
# Imputed	44	40
< LOQ 12 Wks Post EOT	40 (38.8)	39 (38.2)

Viral breakthrough (subjects with a confirmed > 1 log increase in HCV RNA above nadir or confirmed HCV RNA > LOQ after previously being confirmed undetectable) was observed in 5/67 (7.5%) subjects treated with the 180 µg dose of Lambda, compared with 3/62 (4.8%) of alfa-2a-treated evaluable subjects. Relapse was observed in 20/59 [33.9%] of subjects treated with the 180 µg dose of Lambda, compared with 19/58 (32.8%) alfa-2a-treated evaluable subjects.

Safety:

In part B of the phase 2b EMERGE study, reductions in hematologic cell counts were more frequently observed in subjects in the alfa-2a treatment group compared to subjects in the Lambda 180µg dose treatment group. A summary of the treatment emergent hematological abnormalities that were observed in the EMERGE study is provided in Table 1.4.4B.

**Table 1.4.4B: Incidence of Treatment Emergent Hematologic Laboratory Abnormalities by Highest Toxicity Grade Observed (On-Treatment)**			
**Lab Toxicity**			
	**Toxicity Grade**	**Alfa-2a/RBV (N = 103) n/N (%)**	**Lambda(180 µg)/RBV (N = 102) n/N (%)**
Hemoglobin	Evaluable	103/103 (100)	101/102 (99.0)
Low	1	29/103 (28.2)	27/101 (26.7)
	2	32/103 (31.1)	17/101 (16.8)
	3	32/103 (31.1)	6/101 (5.9)
Lymphocytes	Evaluable	103/103 (100)	101/102 (99.0)
Low	1	8/103 (7.8)	1/101 (1.0)
	2	13/103 (12.6)	2/101 (2.0)
	3	9/103 (8.7)	1/101 (1.0)
	4	3/103 (2.9)	0/101 (0.0)
Neutrophils Low	Evaluable	103/103 (100)	101/102 (99.0)
	1	26/103 (25.2)	0/101 (0.0)
	2	23/103 (22.3)	1/101 (1.0)

Approved v4.0 930056810 4.0

Table 1.4.4B: Incidence of Treatment Emergent Hematologic Laboratory Abnormalities by Highest Toxicity Grade Observed (On-Treatment)

Lab Toxicity			
	**Toxicity Grade**	**Alfa-2a/RBV (N = 103) n/N (%)**	**Lambda(180 µg)/RBV (N = 102) n/N (%)**
	3	20/103 (19.4)	1/101 (1.0)
	4	1/103 (1.0)	0/101 (0.0)
Platelets Low	Evaluable	103/103 (100)	101/102 (99.0)
	1	24/103 (23.3)	0/101 (0.0)
	2	18/103 (17.5)	2/101 (2.0)
	3	2/103 (1.9)	0/101 (0.0)
WBC Low	Evaluable	103/103 (100)	101/102 (99.0)
	1	27/103 (26.2)	1/101 (1.0)
	2	27/103 (26.2)	2/101 (2.0)
	3	7/103 (6.8)	1/101 (1.0)

Subjects in the Lambda 180 µg dose treatment group also experienced fewer interferon (see Table 1.4.4C) and RBV (see Table 1.4.4D) dose reductions compared to subjects in the alfa-2a treatment group.

Table 1.4.4C: Incidence of Interferon Related Dose Modifications (On-Treatment)

**Variable**	**Category/Statistic**	**Alfa-2a/RBV (N = 103)**	**Lambda(180 µg)/RBV (N = 102)**
Any Dose Reduction	n (%)	29 (28.2)	8 (7.8)
	95% CI	(19.7, 37.9)	(3.4, 14.9)
Dose Level Reduction per Subject, n (%)	1	22 (21.4)	7 (6.9)
	2	7 (6.8)	1 (1.0)
Subjects with Reductions Due to, n (%)	**Adverse Event**	7 (6.8)	7 (6.9)
	Sponsor Decision	0	0
	Hematologic Abn.	21 (20.4)	0
	Liver Function Abn.	1 (1.0)	1 (1.0)
Doses Held per Subject, n (%)	0	91 (88.3)	91 (89.2)
	1	4 (3.9)	9 (8.8)

Approved v4.0 930056810 4.0

**Table 1.4.4C:**	**Incidence of Interferon Related Dose Modifications (On-Treatment)**		
**Variable**	**Category/Statistic**	**Alfa-2a/RBV (N = 103)**	**Lambda(180 µg)/RBV (N = 102)**
	2	7 (6.8)	2 (2.0)
	3	1 (1.0)	0
Subjects with Held Doses Due to, n (%)	Adverse Event	9 (8.7)	9 (8.8)
	Dose reduced in error	1 (1.0)	0
	Hematologic Abn.	1 (1.0)	0
	Liver Function Abn.	1 (1.0)	2 (2.0)

**Table 1.4.4D:**	**Incidence of Ribavirin Related Dose Modifications(On-Treatment)**		
**Variable**	**Category/Statistic**	**Alfa-2a/RBV (N = 103)**	**Lambda(180 µg)/RBV (N = 102)**
Subjects with Held/Reduced Dose	n (%)	34 (33.0)	11 (10.8)
	95% CI	(24.1, 43.0)	(5.5, 18.5)
Subjects with Held/Reduced Dose due to AE/Abnormality	n (%)	34 (33.0)	10 (9.8)
	95% CI	(24.1, 43.0)	(4.8, 17.3)
Subjects with Reductions Due to, n (%)	Adverse Event	11 (10.7)	4 (3.9)
	Hemoglobin Abn.	24 (23.3)	4 (3.9)
	Other Lab Abn.	0	1 (1.0)
	Other	1 (1.0)	1 (1.0)
Subjects with Held Doses Due to, n (%)	Adverse Event	5 (4.9)	2 (2.0)
	Hemoglobin Abn.	3 (2.9)	0
	Other Lab Abn.	1 (1.0)	0
	Other	0	1 (1.0)

1.4.5 Clinical Experience with Telaprevir

The efficacy and safety profile of TVR in subjects with GT-1 chronic hepatitis C has been well characterized in both treatment-naive subjects, and prior treatment-failure subjects.[49] The key TVR clinical data can be found in the local TVR package insert.

Approved v4.0 930056810 4.0

Efficacy:

Telaprevir-based therapy demonstrated significantly higher SVR rates than alfa-2a/RBV alone across several patient populations {Study 108 (ADVANCE), Study 111 (ILLUMINATE), Study C216 (REALIZE)} and prior treatment-failure subjects.[49]

Among treatment-naive subjects, rates of SVR24 were 72% to 79% in the TVR groups compared with 46% in the placebo group ($p < 0.0001$). Among the prior relapse population, rates of SVR24 were 84% to 88% in the TVR groups compared with 22% in the placebo group ($p < 0.0001$). Among the prior partial responder population, rates of SVR24 were 56% to 61% in the TVR groups compared with 15% in the placebo group ($p < 0.001$). Among the prior null responder population, rates of SVR24 were 31% to 33% in the TVR groups compared with 5% in the placebo group ($p < 0.001$). Majority of treatment naive-patients were eligible to receive 24 weeks of treatment using response-guided therapy, which is half the duration of currently available treatments; significantly higher SVR rates were observed after 24 weeks of TVR based therapy in patients with eRVR compared to alfa-2a/RBV alone.

Overall, 58.4% of patients in Study 108, and 65.2% of patients in Study 111 achieved eRVR and were eligible for 24 weeks of total treatment, instead of 48 weeks standard treatment. Approximately 90% of patients with eRVR achieved SVR after 24 weeks of therapy. Lead-in treatment for 4 weeks with alfa-2a/RBV before TVR treatment in a prior treatment failure population did not improve SVR rates over a simultaneous start. SVR rates were similar among a broad range of subgroups. Substantial clinical benefit, compared to standard therapy, was achieved in treatment-naïve patients who were black, Hispanic or Latino, had cirrhosis or bridging fibrosis, or had high baseline levels of HCV RNA, and in patients who did not achieve SVR with a prior course of alfa-2a/RBV. The responses in the control groups were comparable to those reported in the literature for alfa-2a/RBV for the relevant populations.

The efficacy data support a response-guided regimen of 12 weeks TVR plus 24 weeks alfa-2a/RBV for treatment-naive patients and prior relapsers.

Safety:

The safety assessment is based on data from pooled adequate and well-controlled clinical trials including 1797 subjects who received alfa-2a/RBV/TVR and 493 who received alfa-2a/RBV.

Serious adverse drug reactions occurred in 3% of subjects who received alfa-2a/RBV/TVR compared to none of the subjects who received alfa-2a/RBV. The most frequent serious adverse events in subjects in the alfa-2a/RBV/TVR group were skin disorders (rash and/or pruritus) and anemia. 14% of subjects discontinued TVR due to adverse drug reactions. Rash, anemia, fatigue, pruritus, nausea, and vomiting were the most frequent adverse drug reactions leading to discontinuation of TVR.

Fatal and non-fatal serious skin reactions have been reported in patients treated with alfa-2a/RBV/TVR. Serious skin reactions, including Drug Reaction with Eosinophilia and Systemic Symptoms (DRESS) and Stevens Johnson Syndrome (SJS) were reported in less than 1% of subjects who received alfa-2a/RBV/TVR combination treatment compared to none who

Approved v4.0 930056810 4.0

received alfa-2a and RBV alone. These serious skin reactions reported during clinical trials required hospitalization, and all subjects recovered. Toxic Epidermal Necrolysis (TEN) and Erythema Multiforme (EM) have been observed in post-marketing experience. Fatal cases have been reported in patients with progressive rash and systemic symptoms who continued to receive alfa-2a/RBV/TVR combination treatment after a serious skin reaction was identified.

Table 1.4.5 presents clinical adverse drug reactions occurring with an incidence at least 5% greater in the alfa-2a/RBV/TVR group.

Table 1.4.5: Clinical Adverse Drug Reactions Reported with at Least 5 Percent Higher Frequency Among Subjects Receiving Telaprevir

	**TVR/Alfa/RBV**<br>**N = 1797**	**Alfa/RBV**<br>**N = 493**
Rash[a]	56%	34%
Fatigue	56%	50%
Pruritus	47%	28%
Nausea	39%	28%
Anemia[a]	36%	17%
Diarrhea	26%	17%
Vomiting	13%	8%
Hemorrhoids	12%	3%
Anorectal discomfort	11%	3%
Dysgeusia	10%	3%
Anal pruritus	6%	1%

[a] Rash and anemia based on SSC (Special Search Category) grouped terms.

1.5 Overall Risk/Benefit Assessment

1.5.1 Lambda

<u>Potential Risk of Lambda Treatment:</u>

Based on data from the on-going AI452004 phase 2b study (EMERGE) the key risks associated with treatment using the 180 μg dose of Lambda in treatment naive subjects with chronic HCV GT-1 and/or -4 are:

Approved v4.0 930056810 4.0

a. AST/ALT elevations
b. Direct bilirubin elevations

Details of this study can be found in the Lambda Investigator Brochure.

AI452004 Study (EMERGE)

Part B of the EMERGE phase 2b study (AI452-004) is a randomized control, double-blinded study which evaluated the safety and efficacy of Lambda (180 µg)/RBV in comparison to alfa-2a/RBV.

Safety:

A summary of the incidence of treatment-emergent hepatic laboratory abnormalities in part B of the EMERGE phase 2b study is provided in Table 1.5, below. In the Lambda treatment group, all these AST/ALT and bilirubin elevations stabilized or normalized without sequelae, either spontaneously or with dose modification or discontinuation.

Table 1.5: Incidence of treatment-emergent hepatic laboratory abnormalities, by the highest toxicity grade observed in part B of the EMERGE Study

Lab Toxicity	Toxicity Grade DAIDs Criteria	alfa-2a/RBV (N=103) n/N (%)	Lambda(180 µg)/ RBV (N=102) n/N (%)
ALT High	Evaluable	103/103 (100)	101/102 (99.0)
	G1: 1.25–2.5 x ULN	12/103 (11.7)	15/101 (14.9)
	G2: > 2.5–5.0 x ULN	8/103 (7.8)	13/101 (12.9)
	G3: > 5.0– 10 x ULN	4/103 (3.9)	1/101 (1.0)
AST High	Evaluable	103/103 (100)	101/102 (99.0)
	G1: 1.25–2.5 x ULN	19/103 (18.4)	13/101 (12.9)
	G2: > 2.5–5.0 x ULN	6/103 (5.8)	10/101 (9.9)
	G3: > 5.0–10 x ULN	5/103 (4.9)	2/101 (2.0)
	G4: 10 x ULN	1/103 (1.0)	0/101 (0.0)
ALT and/or AST High	Evaluable	103/103 (100)	101/102 (99.0)
	G1	21/103 (20.4)	20/101 (19.8)
	G2	9/103 (8.7)	14/101 (13.9)
	G3	7/103 (6.8)	3/101 (3.0)
	G4	1/103 (1.0)	0/101 (0.0)
Direct Bilirubin High	Evaluable	103/103 (100)	101/102 (99.0)
	G1: >0.4 – 0.6 mg/dL	9/103 (8.7)	17/101 (16.8)
	G2: >0.6 – 1.2 mg/dL	2/103 (1.9)	6/101 (5.9)

Approved v4.0 930056810 4.0

Table 1.5: Incidence of treatment-emergent hepatic laboratory abnormalities, by the highest toxicity grade observed in part B of the EMERGE Study

Lab Toxicity	Toxicity Grade DAIDs Criteria	alfa-2a/RBV (N=103) n/N (%)	Lambda(180 µg)/ RBV (N=102) n/N (%)
	G3: > 1.2 – 4 mg/dL	1/103 (1.0)	3/101 (3.0)
	G4: > 4.0 mg/dL	1/103 (1.0)	2/101 (2.0)
Total Bilirubin High	Evaluable	103/103 (100)	101/102 (99.0)
	G1: >ULN–1.5x ULN	16/103 (15.5)	27/101 (26.7)
	G2: >1.5–2.5 x ULN	11/103 (10.7)	14/101 (13.9)
	G3: >2.5–5 x ULN	4/103 (3.9)	5/101 (5.0)
	G4: >5 x ULN	1/103 (1.0)	2/101 (2.0)

<u>Potential Benefits of Lambda Treatment:</u>

Based on data from the on-going phase 2b AI452004 study (EMERGE) the key benefits associated with treatment using Lambda, in treatment naive subjects with chronic HCV GT-1 and/or -4, are:

- Comparable SVR12 efficacy data to treatment with alfa-2a/RBV
- Improved early on-treatment (RVR and cEVR) virological responses
- Less cytopenic abnormalities
- Less interferon associated adverse events
- Fewer Lambda and RBV dose reductions

The data summarizing these key benefits have been described in Section 1.4.4. Further details of this study can be found in the Lambda Investigator Brochure.

1.5.2 Telaprevir

Details of the safety and efficacy profile TVR data can be found in the local TVR package insert. A summary of key risk and benefits of treatment with TVR is described below.

<u>Potential Risks of Telaprevir Treatment</u>

The key risks associated with the use of TVR are the following:

a. Rash
b. Serious Skin Reactions
c. Anemia
d. Anorectal Symptoms

Approved v 4.0 930056810 4.0

e. Lymphopenia
f. Thrombocytopenia
g. Hyperbilirubinaemia
h. Hyperuricaemia
i. Resistance

Rash:

In controlled clinical trials, rash events (all grades) were reported in 56% of subjects who received alfa-2a/RBV/TVR and in 34% of subjects who received alfa-2a/RBV. Rash most frequently began during the first 4 weeks, but could occur at any time during alfa-2a/RBV/TVR treatment. Improvement of rash occurs after TVR dosing completion or discontinuation; however, rashes may take weeks for complete resolution.

Rash events led to discontinuation of TVR alone in 6% of subjects and discontinuation of alfa-2a/RBV/TVR combination treatment in 1% of subjects.

Serious Skin Reactions:

Fatal and non-fatal serious skin reactions, including Stevens Johnson Syndrome (SJS), Drug Reaction with Eosinophilia and Systemic Symptoms (DRESS), and Toxic Epidermal Necrolysis (TEN) have been reported in patients treated with alfa-2a/RBV/TVR combination treatment. Fatal cases have been reported in patients with progressive rash and systemic symptoms who continued to receive alfa-2a/RBV/TVR combination treatment after a serious skin reaction was identified.

In clinical trials, serious skin reactions, including DRESS and SJS were reported in less than 1% of subjects who received alfa-2a/RBV/TVR combination treatment compared to none who received alfa-2a and RBV alone. These serious skin reactions reported during clinical trials required hospitalization, and all subjects recovered. The presenting signs of DRESS may include rash, fever, facial edema, and evidence of internal organ involvement (eg, hepatitis, nephritis). Eosinophilia may or may not be present. The presenting signs of SJS may include fever, target lesions, and mucosal erosions or ulcerations (eg, conjunctivae, lips). TEN and Erythema Multiforme (EM) have been observed in post-marketing experience.

Anemia:

In controlled clinical trials, the overall incidence and severity of anemia increased with alfa-2a/RBV/TVR combination treatment compared with alfa-2a/RBV alone. The incidence of anemia AEs was 36% with alfa-2a/RBV/TVR compared to 17% with alfa-2a/RBV alone. A decrease in hemoglobin levels occurred during the first 4 weeks of treatment, with lowest values reached at the end of TVR dosing. Hemoglobin values gradually returned to levels observed with alfa-2a/RBV after TVR dosing was completed.

Approved v4.0 930056810 4.0

Anorectal Signs and Symptoms:

In the controlled clinical trials, 29% of subjects treated with alfa-2a/RBV/TVR experienced anorectal AEs, compared to 7% of those treated with alfa-2a/RBV alone. The majority of these events (eg, hemorrhoids, anorectal discomfort, anal pruritus, and rectal burning) were mild to moderate in severity; less than 1% led to treatment discontinuation and all resolved during or after completion of TVR dosing.

Laboratory abnormalities

White Blood Cells:

More TVR-treated subjects had decreases in lymphocyte counts to 499/mm^3 or less (15% compared to 5%). Decreases in total white cell counts to 1,499/mm^3 or less were comparable (8% compared to 5%). The incidence of decreases in absolute neutrophil counts to 749/mm^3 or less was 15% in subjects treated with alfa-2a/RBV alone compared to 12% among those treated with alfa-2a/RBV/TVR combination treatment.

Platelets:

More subjects treated with alfa-2a/RBV/TVR had decreases in mean platelet values of all grades: 47% compared to 36% treated with peg-interferon alfa and ribavirin alone. Three percent of TVR combination treatment subjects had decreases to 49,999/mm^3 or less compared to 1% of those treated with alfa-2a/RBV alone.

Bilirubin:

Forty-one percent of subjects in the alfa-2a/RBV/TVR group compared to 28% of subjects in the alfa-2a/RBV group had all grade elevations in bilirubin levels; 4% and 2% of subjects, respectively, had greater than or equal to 2.6 x ULN elevations. Bilirubin levels increased most steeply during the first 1 to 2 weeks of TVR dosing, stabilized and between Weeks 12 and 16 were at baseline levels.

Uric Acid:

During the treatment period, 73% of subjects in the alfa-2a/RBV/TVR group had elevated uric acid levels compared to 29% of subjects in the alfa-2a/RBV group. Shifts to greater than or equal to 12.1 mg/dL from baseline in uric acid levels were also more frequent among subjects treated with alfa-2a/RBV/TVR (7%) compared to alfa-2a/RBV alone (1%). Less than 1% of subjects had clinical events of gout/gouty arthritis; none were serious and none resulted in treatment discontinuation.

Resistance:

Clinical virology results from clinical studies of TVR in combination with alfa and RBV have shown a clear and consistent resistance profile across HCV GT-1 patient populations (treatment-naive and prior alfa/RBV treatment-failure). Sequence analyses in patients not achieving an SVR with a telaprevir-based regimen consistently identified amino acid substitutions at 4 positions in the NS3•4A protease region that were associated with decreased

Approved v4.0 930056810 4.0

sensitivity to TVR, consistent with the mechanism of action for TVR: V36A/M, T54A/S, R155K/T, A156S/T/V, and V36M+R155K. Phenotypic characterization of these HCV NS3 variants determined that lower-level resistance to TVR (3- to 25-fold decrease in IC_{50} to telaprevir in a replicon-based assay) was conferred by V36A/M, T54A/S, R155K/T, and A156S, and higher-level resistance to TVR (> 25-fold decrease in replicon IC_{50}) was conferred by A156T/V and V36M+R155K. TVR-resistant variants are less fit than wild-type virus and are sensitive to alfa, RBV, and polymerase inhibitors in vitro.

Predominant baseline resistance to TVR is rare (< 1% to 2.7%) and does not necessarily preclude achieving an SVR with a TVR/alfa/RBV regimen. On-treatment virologic failure during TVR treatment is associated with higher-level TVR-resistant variants, and occurs more frequently in GT-1a compared to -1b. On-treatment virologic failure rates on TVR/alfa/RBV are low in treatment-naive patients, and prior relapser patients, but are higher for prior nonresponders patients. Relapse is generally associated with wild-type or lower-level resistant variants.

Overall, TVR-resistant variants were observed in 12% of treatment-naïve patients (Study 108 ADVANCE; T12/PR arm)[49] and 22% of treatment-experienced patients, after therapy with a TVR-containing regimen. Resistant variants were observed in the majority of subjects who did not achieve an SVR. Resistant variants tend to be replaced by wild-type virus over time in the absence of TVR selective pressure.

Cross-Resistance:

Treatment-emergent NS3 amino acid substitutions detected in TVR-treated subjects who did not achieve SVR in the clinical trials (substitutions at positions V36, T54, R155, A156) have been demonstrated to reduce the anti-HCV activity of boceprevir and other HCV NS3/4A protease inhibitors. The impact of prior TVR exposure or treatment failure on the efficacy of boceprevir or other HCV NS3/4A protease inhibitors has not been studied. TVR efficacy has not been established for patients with a history of exposure to NS3/4A protease inhibitors.

Cross-resistance is not expected between TVR and interferons, or TVR and RBV. HCV replicons expressing TVR-associated resistance substitutions remained fully sensitive to interferon alfa and ribavirin, as well as other direct-acting antivirals with different mechanisms of action, such as NS5B polymerase inhibitors.

Potential Benefits of Telaprevir Treatment

The key benefits associated with the use of TVR are the following:

a. Improved SVR24
b. Possibility for shortened duration of treatment

The key efficacy and shortened duration of treatment benefits associated with the treatment of TVR have been described in section 1.4.5. Further details may be found in the local TVR package insert.

Approved v4.0 930056810 4.0

2 ETHICAL CONSIDERATIONS

2.1 Good Clinical Practice

This study will be conducted in accordance with Good Clinical Practice (GCP), as defined by the International Conference on Harmonization (ICH) and in accordance with the ethical principles underlying European Union Directive 2001/20/EC and the United States Code of Federal Regulations, Title 21, Part 50 (21CFR50).

The study will be conducted in compliance with the protocol. The protocol and any amendments and the subject informed consent will receive Institutional Review Board/Independent Ethics Committee (IRB/IEC) approval/favorable opinion prior to initiation of the study.

All potential serious breaches must be reported to BMS immediately. A serious breach is a breach of the conditions and principles of GCP in connection with the study or the protocol, which is likely to affect, to a significant degree, the safety or physical or mental integrity of the subjects of the study or the scientific value of the study.

Personnel involved in conducting this study will be qualified by education, training, and experience to perform their respective tasks.

This study will not use the services of study personnel where sanctions have been invoked or where there has been scientific misconduct or fraud (eg, loss of medical licensure, debarment).

2.2 Institutional Review Board/Independent Ethics Committee

Before study initiation, the investigator must have written and dated approval/favorable opinion from the IRB/IEC for the protocol, consent form, subject recruitment materials (eg, advertisements), and any other written information to be provided to subjects. The investigator or BMS should also provide the IRB/IEC with a copy of the Investigator Brochure or product labeling information to be provided to subjects and any updates.

The investigator or BMS should provide the IRB/IEC with reports, updates and other information (eg, expedited safety reports, amendments, and administrative letters) according to regulatory requirements or institution procedures.

2.3 Informed Consent

Investigators must ensure that subjects are clearly and fully informed about the purpose, potential risks, and other critical issues regarding clinical studies in which they volunteer to participate.

In situations where consent cannot be given to subjects, their legally acceptable representatives are clearly and fully informed about the purpose, potential risks, and other critical issues regarding clinical studies in which the subject volunteers to participate.

BMS will provide the investigator with an appropriate (ie, Global or Local) sample informed consent form which will include all elements required by ICH, GCP and applicable regulatory requirements. The sample informed consent form will adhere to the ethical principles that have their origin in the Declaration of Helsinki.

Approved v4.0 930056810 4.0

Investigators must:

1) Provide a copy of the consent form and written information about the study in the language in which the subject is most proficient prior to clinical study participation. The language must be non-technical and easily understood.
2) Allow time necessary for subject or subject's legally acceptable representative to inquire about the details of the study.
3) Obtain an informed consent signed and personally dated by the subject or the subject's legally acceptable representative and by the person who conducted the informed consent discussion.
4) Obtain the IRB/IEC’s written approval/favorable opinion of the written informed consent form and any other information to be provided to the subjects, prior to the beginning of the study, and after any revisions are completed for new information.
5) If informed consent is initially given by a subject’s legally acceptable representative or legal guardian, and the subject subsequently becomes capable of making and communicating his or her informed consent during the study, consent must additionally be obtained from the subject.
6) Revise the informed consent whenever important new information becomes available that is relevant to the subject's consent. The investigator, or a person designated by the investigator, should fully inform the subject or the subject's legally acceptable representative or legal guardian, of all pertinent aspects of the study and of any new information relevant to the subject's willingness to continue participation in the study. This communication should be documented.

The confidentiality of records that could identify subjects must be protected, respecting the privacy and confidentiality rules applicable to regulatory requirements, the subjects' signed ICF and, in the US, the subjects’ signed HIPAA Authorization.

The consent form must also include a statement that BMS and regulatory authorities have direct access to subject records.

The rights, safety, and well-being of the study subjects are the most important considerations and should prevail over interests of science and society.

3 INVESTIGATIONAL PLAN

3.1 Study Design and Duration

AI452020 is a Phase 3 clinical study in GT-1 chronic HCV infected subjects who are treatment-naïve or who are prior relapsers to alfa/RBV combination treatment. This study has two parts. Part A is a single arm, open label sentinel cohort. Part B is a randomized controlled, double-blinded cohort. All subjects will be treated for 24 or 48 weeks.

3.1.1 Part A: Open Label Sentinel Cohort

Part A is an open-label, single arm study involving approximately 25 subjects with GT-1 chronic HCV infection. Subjects will be treated with Lambda/RBV/TVR (see Figure 3.1.1).

Approved v4.0 930056810 4.0

Treatment-naive subjects with HCV GT-1b subtype will be capped at approximately 50%. Relapsers will be capped at approximately 20%. Subjects with compensated cirrhosis will be capped at approximately 10%.

HCV RNA will be made available to investigators.

Treatment Period:

On Day 1, subjects will start treatment with Lambda/RBV/TVR.

- TVR will only be administered for the first 12 weeks of treatment
- Cirrhotic and non-cirrhotic subjects achieving eRVR (HCV RNA < LLOQ target not detected at Week 4 and 12 of treatment) will continue treatment for a further 12 weeks with only Lambda/RBV to complete a total of 24 weeks of treatment.
- Cirrhotic and non-cirrhotic subjects not achieving eRVR will continue treatment for a further 36 weeks with only Lambda/RBV to complete a total of 48 weeks of treatment.

Follow-Up Period:

Subjects will be followed-up, off treatment, according to the post treatment follow-up schedule in Table 5.1C, for 48 weeks to assess virologic response at follow-up Week 24 (SVR24), durability of the sustained virologic response or to evaluate treatment emergent resistance. Subjects who wish to initiate alternative anti-HCV treatment during post-treatment follow-up must discontinue the study, however, they must first complete a minimum of 4 weeks of follow-up before discontinuing the study.

After the treatment and follow-up period, subjects treated with Lambda who continue to demonstrate SVR may be asked to enroll in a separate follow-up observational study to evaluate long-term virologic durability of the Lambda treatment regimen and HCV-related complications.

Approved v4.0 930056810 4.0

Figure 3.1.1: Part A - Open Label Sentinel Cohort (N = 25)

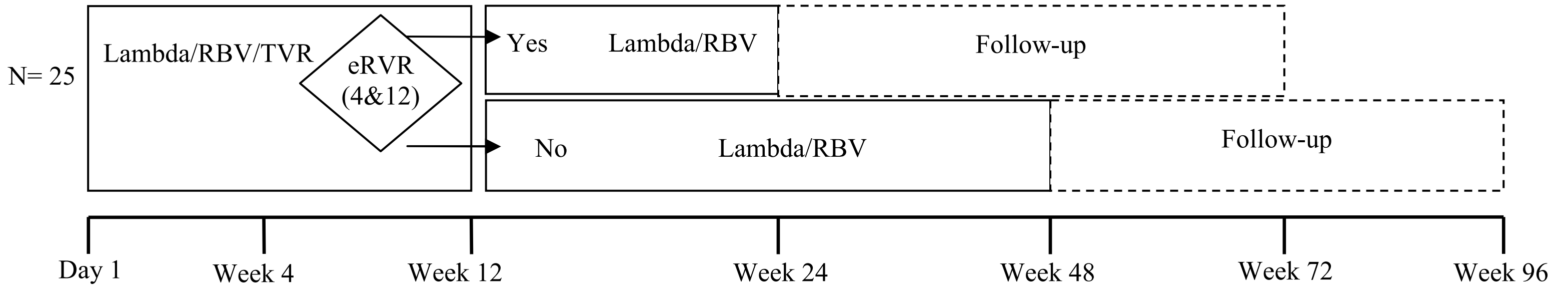

See futility criteria described in Section 3.1.3.

Approved v 4.0 930056810 4.0

Transition to Part B will depend on whether results of Part A show an acceptable safety profile and meet go-no-go criteria (described below).

Go/No-go Decision to Part B

After all the subjects have completed 6 weeks of treatment, the discontinuation rate will be reviewed. If the rate of drug-related AEs leading to discontinuation is ≤ 12%, then Part B may proceed in parallel, while subjects continue on study in Part A.

An interim analysis of Part A will be conducted after all subjects have completed 12 weeks of treatment. The study will continue in Part B only if results in Part A meet the primary safety criteria (an observed rate of ≤ 24% of drug-related AEs leading to discontinuation).

3.1.2 Part B: Randomized, Controlled, Double-Blinded Cohort

Part B is a randomized, double-blind, active controlled phase of the study. Approximately 609 subjects with GT-1 chronic HCV infection will be randomized (2:1) to one of two treatment arms:

1) Lambda in combination with RBV/TVR (N = 406)
2) alfa-2a in combination with RBV/TVR (N = 203)

Part B will include subjects who are either treatment naive or who are relapsers to previous alfa/RBV treatment. Randomization will be stratified by prior relapse status, IL-28B CC and non-CC status, and viral GT-1a and -1b subtypes. Subjects with HCV GT-1b subtype will be capped at approximately 50%. Relapsers will be capped at approximately 20%. Subjects with compensated cirrhosis will be capped at approximately 10%.

Recent data on the risks of hepatic decompensation of an IFN-containing triple regimen with TVR is provided by the ANRS CUPIC study.[39] In this study, there were 292 compensated cirrhotic (Child Pugh A) nonresponders that were treated with alfa/RBV/TVR, and 33.1% had esophageal varices at baseline. In this interim analysis, infections were seen in 6.5% and new decompensation was seen in 2.0% of subjects treated with the TVR triple regimen. Compensated cirrhotic subjects classified as Child Pugh A may have clinically significant portal hypertension, putting them at high intrinsic risk for decompensation events. Indicators of significant portal hypertension include hepatic venous pressure gradient (HVPG) ≥ 10 mm Hg[40], presence of esophageal or abdominal varices, any splenomegaly on imaging > 12 cm (diameter)[41], and a Fibroscan score ≥ 21 kPa.[42]

Compensated cirrhotic subjects without evidence of portal hypertension will be eligible for the study (see Section 3.3.2 - Exclusion Criteria 2a and 2b). Data evaluating portal hypertension and other relevant clinical and laboratory data must be reviewed by the BMS Central Medical Monitor prior to randomization.

Approved v4.0 930056810 4.0

Treatment period:

On Day 1, subjects will start treatment with Lambda/RBV/TVR or alfa-2a/RBV/TVR.

- TVR will only be administered for the first 12 weeks of treatment.
- Noncirrhotic subjects achieving eRVR (HCV RNA < LLOQ target not detected at Week 4 and 12 of treatment) will continue treatment for a further 12 weeks with Lambda/RBV or alfa-2a /RBV to complete a total of 24 weeks of treatment.
- Noncirrhotic subjects not achieving eRVR will continue treatment for a further 36 weeks with Lambda/RBV or alfa-2a/RBV to complete a total of 48 weeks of treatment.
- All cirrhotic subjects will continue treatment for a further 36 weeks with Lambda/RBV or alfa-2a/RBV to complete a total of 48 weeks of treatment.

Follow-up period:

- Subjects will be followed-up, off treatment, according to the post treatment follow-up schedule in Table 5.1C, for 48 weeks to assess virologic response at follow-up Week 24 (SVR24), durability of the sustained virologic response or to evaluate treatment emergent resistance. Subjects who wish to initiate alternative anti-HCV treatment during post-treatment follow-up must discontinue the study, however, they must first complete a minimum of 4 weeks of follow-up before discontinuing the study.

Figure 3.1.2: Part B - Randomized, IFN-blinded, Non-inferiority Design (N = 609)

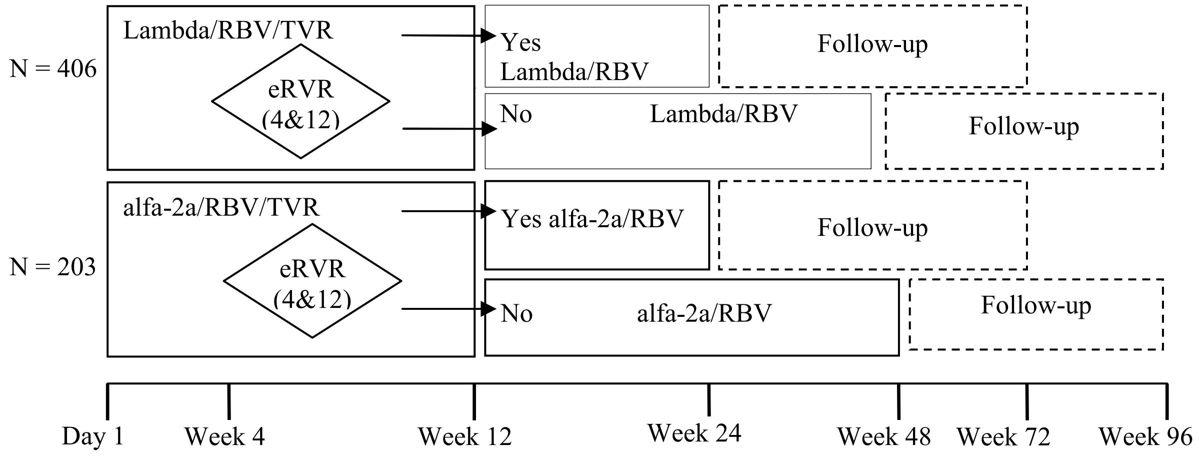

See futility criteria described in Section 3.1.3.

Approved v 4.0 930056810 4.0

After the treatment and follow-up period, subjects treated with Lambda who demonstrate continued SVR may be asked to enroll in a separate follow-up observational study to assess long-term virologic response and HCV-related complications.

3.1.3 Futility Criteria for Parts A and B

Discontinue all drugs if:

- Week 4 or 12: HCV RNA > 1000 IU/mL
- Week 24: confirmed HCV RNA detected
- Virologic Breakthrough:

  a. Confirmed* > 1 $\log_{10}$ increase in HCV RNA over nadir

  OR

  b. Confirmed* HCV RNA ≥ LLOQ after previously having an HCV RNA level of < LLOQ (target detected or target not detected) while on treatment.

  *Measurements should ideally be confirmed within 2 weeks of the original result

The Week 4, 12 and 24 futility criteria is based on the futility criteria in the local Telaprevir labels. The breakthrough criteria identify subjects who may potentially have developed resistance to the treatment regimen and as such may not benefit from further treatment.

3.2 Post Study Access to Therapy

At the end of the study, BMS will not continue to supply study drug to subjects/investigators unless BMS chooses to extend the study. The investigator should ensure that the subject receives appropriate standard of care to treat the condition under study.

3.3 Study Population

The study population of this study will consist of subjects infected with GT-1 chronic HCV infection. Re-evaluation to confirm eligibility criteria within the 42 day screening period will be allowed. However, if a subject does not meet the screening requirements within the 42 day screening period, this subject may be re-enrolled only after consultation with the study team. Relevant data evaluating portal hypertension and other relevant clinical or laboratory data must be reviewed by the BMS Central Medical Monitor prior to randomization of cirrhotic subjects.

For entry into the study, the following criteria must be met.

Approved v4.0 930056810 4.0

3.3.1 Inclusion Criteria

1) Signed Written Informed Consent

a) Freely given informed consent must be obtained from patients prior to clinical trial participation including informed consent for any screening procedures conducted to establish patient eligibility for the study.

2) Target Population

a) Subjects must be:

i. Naïve to prior anti-HCV therapy (IFN and DAA based) **OR,**

ii. Relapsers, defined as subjects who had HCV RNA < LLOQ, target not detected at end of treatment with a prior alfa/RBV regimen and a HCV RNA ≥ LLOQ during the follow-up period after treatment. Relapsers previously treated with Lambda will not be excluded.

Documentation of HCV RNA results from prior regimen should be available at the time of enrollment. The relapser population will be capped at approximately 20%.

b) Subjects with GT-1a or -1b subtypes chronic HCV infection (the naive population with GT-1b will be capped at approximately 50%). Genotype testing results should be available at the time of randomization. Mixed genotypes are not allowed.

c) Compensated cirrhotic and non-cirrhotic subjects chronically infected with HCV as documented by either:

i. positive anti-HCV antibody, HCV RNA or a positive HCV genotype test at least 6 months prior to screening; or

ii. Liver biopsy consistent with chronic HCV infection at any time prior to screening.

d) HCV RNA viral load by PCR ≥ 100,000 IU/mL at screening

e) Subjects must have one of the following assessments to evaluate for cirrhosis based on local requirements:

i. Liver biopsy:

For eligible non-cirrhotic subjects, liver biopsy results consistent with chronic HCV (evidence of fibrosis and/or inflammation) must be obtained within 3 years prior to enrollment. For eligible compensated cirrhotic subjects, biopsy documenting cirrhosis can be from any time period prior to randomization. Non cirrhotic subjects must have a documented liver biopsy with an Ishak score ≤ 4 or Metavir fibrosis score ≤ 3, or equivalent (Batts-Ludwig, etc). Compensated cirrhotic subjects must have a documented liver biopsy with an Ishak fibrosis score of ≥ 5 or a Metavir fibrosis score of 4, or equivalent (Batts-Ludwig, etc).

Approved v4.0 930056810 4.0

ii. Fibroscan:

For countries where liver biopsy is not required prior to treatment and where non-invasive imaging tests (Fibroscan ultrasound) are standard of care for staging of liver disease, a Fibroscan done prior to screening is acceptable if it was performed within one year of screening (≥ 14.6 kPa should be considered consistent with cirrhosis[43]). If the prior Fibroscan was not performed within one year of screening, a new Fibroscan is required before study drug dosing. If a subject has both liver biopsy and Fibroscan, the results of the liver biopsy take precedence over those of the Fibroscan.

Subjects with compensated cirrhosis will be capped at approximately 10%.

iii. Screening for portal hypertension:

All cirrhotic subjects must have at least one investigation to assess for the presence of portal hypertension (abdominal ultrasound scan or Fibroscan at a minimum). If a subject has had additional evaluations for liver disease, then all prior observations must meet the following criteria in order for the subject to be eligible for treatment: a hepatic venous pressure gradient < 10 mm Hg, no history or findings/evidence of ascites or varices; any splenomegaly on imaging must be < 12 cm (diameter), and Fibroscan score must be < 21 kPa (see Section 3.3.2 exclusion criteria 2a and 2b). These data must be reviewed by the BMS Central Medical Monitor prior to randomization.

f) Body Mass Index (BMI) of 18 to 35 kg/m^2, inclusive. BMI = weight (kg)/$[height (m)]^2$ at screening

3) Age and Reproductive Status

See Section 3.3.3 for the definition of WOCBP

a) Men and women, ages 18 years of age and above

b) Women of childbearing potential (WOCBP) must use two (2) separate highly effective methods of birth control (from list below) throughout the duration of the on-treatment study period and for up to 24 weeks after the last dose of study drug or of RBV (or the time specified by the country-specific RBV label, whichever is longer) to minimize the risk of pregnancy. WOCBP must follow instructions for birth control for the entire duration of the study including a minimum of 24 weeks (or the time specified by the country-specific RBV label, whichever is longer) after dosing has been completed.

Acceptable methods of highly effective birth control for this study include (must use 2 of the listed methods):

- Condom (male or female) with spermicide*
- Diaphragm and spermicide*
- Cervical cap and spermicide*

Approved v4.0 930056810 4.0

- Sponge and spermicide*
- IUD**
- Vasectomized male for a minimum of 6 months

*As noted above, these methods generally require a spermicide except in countries where it may not be available

**The use of IUDs shall be at the discretion of the investigator if it is permitted by local regulations.

Female subjects receiving study drug may use hormonal contraceptives however, it cannot be considered one of the two forms of effective birth control required because a drug interaction study verifying the effectiveness of hormone based contraceptives have not been conducted with Lambda.

c) Women must have a negative serum or urine pregnancy test (minimum sensitivity 25 IU/L or equivalent units of HCG) within 24 hours prior to the start of investigational product.

d) Women must not be breastfeeding

e) Sexually active men must use two (2) separate highly effective methods of birth control if their partners are WOCBP. Men that are sexually active with WOCBP must follow instructions for birth control for the entire duration of the study and a minimum of 24 weeks after the last dose of study drugs or of RBV (or the time specified by the country-specific RBV label, whichever is longer).

Requirements for male subjects (based on RBV label):

i) Male subjects with female partners who are WOCBP must agree to inform their female partners of the protocol-specified contraception requirement and pregnancy testing recommendations during treatment and post-treatment (ie, 2 forms of contraception and monthly pregnancy testing while the subject is enrolled in the study and 24 weeks following discontinuation of RBV [or for the post-treatment duration specified in the country-specific RBV label, whichever is longer]), and agree to adhere to these recommendations both on-treatment and during the post-treatment follow-up period.

ii) Male subjects must confirm that their female sexual partners are not pregnant at the time of screening.

iii) Acceptable methods of contraception for males are:

(a) Condom plus spermicide (this method generally requires a spermicide except in countries where it may not be available)

(b) Vasectomy for at least 6 months and with a history of confirmed azoospermia

Approved v4.0 930056810 4.0

f) Female partners of male subjects participating in the study may use hormone based contraceptives as one of the acceptable methods of contraception since they will not be receiving study drug.

3.3.2 Exclusion Criteria

1) Target Disease Exceptions

a) Infected with HCV other than GT-1a and GT-1b subtypes. Mixed genotypes are not allowed

b) Positive HBsAg or HIV-1/HIV-2 antibody at screening

c) Evidence of a medical condition associated with chronic liver disease other than HCV (such as but not limited to: hemochromatosis, autoimmune hepatitis, alcoholic liver disease, biliary disease, nonalcoholic hepatic steatosis, and toxin exposure)

d) Previous exposure to DAA treatment or HCV experimental agents

e) Previous exposure to IFN based treatment (excluding relapsers)

2) Medical History and Concurrent Diseases

a) Current evidence of or history of portal hypertension, known hepatic venous pressure gradient ≥ 10 mm Hg, varices or variceal bleeding, splenomegaly > 12 cm (diameter), hepatic encephalopathy, or ascites requiring diuretics or paracentesis or evidence of any of these findings on physical examination performed at screening

b) Fibroscan scores ≥ 21 kPa if Fibroscan is used to assess staging of liver fibrosis

NOTE: for criteria a) and b) above, all cirrhotic subjects must have at least one investigation to assess for the presence of portal hypertension (abdominal ultrasound scan or Fibroscan at a minimum). If a subject has had documentation of any of the following findings, then these constitute evidence for the presence of portal hypertension and the subject must be excluded: history of hepatic venous pressure gradient ≥ 10 mm Hg, any history or documentation of ascites or varices, any splenomegaly on imaging > 12 cm (diameter), or Fibroscan score ≥ 21 kPa. These data must be reviewed by the BMS Central Medical Monitor before randomization.

c) Current evidence of or history of pancreatitis

d) Current evidence of or history of renal dialysis, including hemodialysis or peritoneal dialysis

e) History of bone marrow or organ transplant, with the exception of corneal transplants and skin grafts, or therapy with an immunomodulatory agent, cytotoxic agent, or systemic corticosteroids within 2 months of screening

f) Current or known history of cancer (except adequately treated in situ carcinoma of the cervix, or basal or squamous cell carcinoma of the skin) within 5 years prior to screening

g) Subjects with historical or current electrocardiogram (ECG) finding indicative of cardiovascular instability, including but not limited to evidence of myocardial ischemia,

Approved v4.0 930056810 4.0

unstable re-entry phenomena, and/or other clinically significant dysrhythmias as determined by the Investigator. Subjects with QTcF > 500 mSec will be excluded.

h) Active substance abuse, such as alcohol, or inhaled or injected drugs, as defined by Diagnostic and Statistical Manual of Mental Disorders IV (DSM-IV), Diagnostic Criteria for Drug and Alcohol Abuse (Appendix 1) within 6 months prior to screening. Subjects who are receiving methadone or other substitutive product under medical supervision are eligible.

i) Current or known history of cardiomyopathy or clinically significant ischemic cardiac/valvular or cerebrovascular disease, including history of angina, myocardial infarction, interventional procedure for coronary artery/valvular disease (including angioplasty, stent procedure, or cardiac bypass surgery)

j) Confirmed uncontrolled hypertension (patients with screening systolic blood pressure > 160 mmHg or diastolic blood pressure > 100 mmHg should be excluded).

k) Current or known history of clinically significant hemoglobinopathy or hemolytic anemia

l) Subjects with pre-existing ophthalmologic disorders considered clinically significant on eye (including retinal) exam. The eye (including retinal) exam may be done by the investigator; however if a subject has underlying diabetes, hypertension or history of preexisting eye disease, a complete dilated eye exam should be done by an eye care professional.

m) Current or known history of severe chronic obstructive lung disease, interstitial lung disease or sarcoidosis

n) History of immunologically mediated disease (including, but not limited to, rheumatoid arthritis, inflammatory bowel disease, moderate to severe psoriasis [mild psoriasis is allowed], and systemic lupus erythematosus)

o) History of or current severe psychiatric disease, especially untreated or unstable depression as judged by the investigator, or psychotic disorder, such as bipolar disease, or history of hospitalization for suicidal ideation/attempt

p) Active seizure disorder as defined by either untreated seizure disorder or continued seizure activity within the past year prior to screening despite treatment with anti-seizure medication

q) Has, in the opinion of the investigator, any physical exam findings, laboratory abnormalities, or other medical, social, or psychosocial factors that may negatively impact compliance or subject’s safety by participation in this study; this should include conditions which may affect hematologic parameters such as prior or current history of porphyria cutanea tarda and/or hemophilia

r) Current evidence or known history of decompensated cirrhosis based on radiologic criteria or biopsy results and clinical criteria

s) Any gastrointestinal disease or surgical procedure that may impact the absorption of study drug

t) Inability to tolerate oral medication

Approved v4.0 930056810 4.0

u) Currently lactating or breast-feeding

v) Current use of heparin or coumadin

w) Received blood products within 30 days prior to study randomization

x) Use of hematologic growth factors within 90 days prior to study randomization

y) Systemic antibiotics, antifungals, or antivirals for treatment of active infection or for prophylactic use, within 14 days of randomization

z) Exposure to any investigational drug or placebo within 30 days of study drug administration

aa) Any criteria that would exclude the subject from receiving alfa-2a, RBV or TVR treatment

bb) Evidence of serious or severe bacterial infection or fungal infection(s) including active tuberculosis

3) Physical and Laboratory Test Findings

a) Confirmed Hemoglobin ≤ 12 g/dL (120 g/L) for women and ≤ 13 g/dL (130 g/L) for men

b) Confirmed platelet count < 90,000/mm^3

c) Confirmed creatinine clearance (CrCl) (as estimated by Cockcroft and Gault) ≤ 50 mL/min

d) Confirmed total serum bilirubin ≥ 2 mg/dL (≥ 34 mmol/L) (unless due to Gilbert's disease)

e) Confirmed INR ≥ 1.5

f) PTT ≥ 1.5 x ULN

g) Confirmed serum albumin ≤ 3.5 g/dL (35 g/L)

h) Confirmed ALT ≥ 5 x ULN at screening

i) Confirmed ANC ≤ 1.5 x 10^9 cells/L (for Black subjects, ANC ≤ 1.2 x 10^9)

j) Confirmed potassium < 3.5 mmol/L

k) Diagnosed or suspected hepatocellular carcinoma as evidenced by screening alfa-fetoprotein (AFP) of ≥ 100 ng/mL. If AFP is ≥ 50 ng/mL but < 100ng/mL at screening, absence of a mass must be demonstrated by US/CT/MRI imaging within the screening period.

l) Poorly controlled diabetes mellitus as evidenced by HbA1C ≥ 8.5% at screening

m) Antinuclear antibody (ANA) titer ≥ 1:640 at screening and/or evidence of autoimmune hepatitis on liver biopsy

n) Thyroid-stimulating hormone (TSH) < 0.8 x LLN or > 1.2 x ULN of the laboratory reference range, unless:

   i) The subject is clinically euthyroid as determined by the investigator, AND

Approved v4.0 930056810 4.0

ii) free T4 is ≥ 0.8 x LLN and ≤ 1.2 x ULN

o) Evidence of hepatic decompensation in cirrhotic subjects: history of ascites, hepatic encephalopathy, or bleeding esophageal varices, and/or screening laboratory results as described above.

4) Allergies and Adverse Drug Reaction

a) History of hypersensitivity to drugs with a similar biochemical structure to Lambda or Alfa (e.g., other interferons) or RBV or TVR

b) Any other known contraindication to alfa, RBV or TVR

c) Hypersensitivity to tartrazine (yellow dye)

5) Sex and Reproductive Status

a) Sexually active fertile men whose partners are pregnant at screening are excluded from this study (a contraindication for RBV use).

6) Other Exclusion Criteria

a) Prisoners or subjects who are involuntarily incarcerated

b) Subjects who are compulsorily detained for treatment of either a psychiatric or physical (eg, infectious disease) illness

Eligibility criteria for this study have been carefully considered to ensure the safety of the study subjects and that the results of the study can be used. It is imperative that subjects fully meet all eligibility criteria.

3.3.3 Women of Childbearing Potential

Women of childbearing potential (WOCBP) include any female who has experienced menarche and who has not undergone successful surgical sterilization (hysterectomy, bilateral tubal ligation, or bilateral oophorectomy), and who is not postmenopausal. Post menopause is defined as:

- Amenorrhea ≥ 12 consecutive months without another cause, and a documented serum follicle stimulating hormone (FSH) level > 35 mIU/mL or
- Women with irregular menstrual periods and a documented serum follicle stimulating hormone (FSH) level > 35 mIU/mL or

  NOTE: FSH level testing is not required for women ≥ 62 years old with amenorrhea of ≥ 1 year
- Women on hormone replacement therapy (HRT) <u>who have prior clinical evidence of menopause based on any of the criteria above.</u>

Women who are using oral or other hormonal contraceptives, such as vaginal products, skin patches, or implanted or injectable products, or mechanical products, such as an intrauterine device or barrier methods (diaphragm, condoms, spermicides), to prevent pregnancy or who are

Approved v4.0 930056810 4.0

practicing abstinence or who have a sterile (eg, vasectomy) partner should be considered to be of childbearing potential.

3.4 Concomitant Treatments

Medications taken within 4 weeks prior to administration of study medication must be recorded on the CRF.

3.4.1 Prohibited and/or Restricted Treatments

- The following growth factors are **prohibited** including granulocyte colony stimulating factor (G-CSF) or granulocyte macrophage colony, stimulating factor (GM-CSF), Pegylated G-CSF, Eltrombopag, Romiplostim, or Oprelvekin;
- Erythropoietic growth factors such as erythropoietin will not be reimbursed by the Sponsor. Although discouraged, during the first 12 weeks of treatment with TVR, erythropoietic growth factors may be used at the discretion of the Investigator if anemia persists despite dose reductions of RBV. The use of these growth factors is not permitted after the first 12 weeks of treatment.
- Medications with known or potential anti-HCV activity other than the assigned study treatment are **prohibited**;
- Long-term treatment (≥ 2 weeks) with agents that are immunosuppressive, or have a high risk for nephrotoxicity or hepatotoxicity, should be discussed with the BMS central medical monitor.
- Any prescription or herbal product which is not prescribed by the investigator or licensed physician for treatment of a specific clinical condition is prohibited
- The use of antibiotics, antifungals and non-HCV antivirals are permitted during the study.

3.4.1.1 Prohibited/Restricted during Dosing of TVR

- TVR is an inhibitor of CYP3A. Co-administration of TVR with drugs that are primarily metabolized by CYP3A may result in increased plasma concentrations of patient drugs, which could increase or prolong their therapeutic effect and adverse reactions.
- TVR is also an inhibitor of P-gp. Co-administration of TVR with drugs that are substrates for P-gp transport may result in increased plasma concentrations of such drugs, which could increase or prolong their therapeutic effect and adverse reactions.
- If dose adjustments of concomitant medications are made during TVR treatment, they should be readjusted after administration of TVR is completed.
- The following drugs are contraindicated for use with TVR; Alfuzosin, Rifampin, Dihydroergotamine, Ergonovine, Ergotamine, Methylergonovine, Cisapride, St. John's wort, Atorvastatin, Lovastatin, Simvastatin, Pimozide, Sildenafil, Tadalafil, oral Midazolam, oral Triazolam, Amiodarone, Bepridil, Quinidine, Astemizole, Terfenadine, Class Ia or III antiarrhythmics except for intravenous lidocaine, carbamazepine, phenytoin and phenobarbital. Please reference current label.

Approved v4.0 930056810 4.0

- TVR is a substrate of CYP3A and P-gp; therefore, drugs that induce CYP3A and/or P-gp may decrease TVR plasma concentrations and reduce the therapeutic effect of TVR. Co-administration of TVR with drugs that inhibit CYP3A and/or P-gp may increase TVR plasma concentrations.
- Co-administration of systemic corticosteroids and telaprevir is not recommended due to potentially significant drug interactions.
- Other drugs may have the potential to affect TVR. Refer to the local TVR product labels for further information.

3.4.2 Other Restrictions and Precautions

Subjects should be on stable doses of non-study medications for at least 4 weeks prior to the first dose of study drug. If the subject is on chronic medications, a consistent dosing schedule is recommended for the duration of this study when medically possible.

Assessment of concomitant medications will be performed at each on-treatment visit.

Subjects receiving theophylline therapy should undergo frequent monitoring of serum theophylline levels due to the identified increase in theophylline levels with alfa.

3.5 Discontinuation of Subjects from Treatment

Subjects must discontinue investigational product (and non-investigational product at the discretion of the investigator) for any of the following reasons:

- Withdrawal of informed consent (subject's decision to withdraw for any reason)
- Any clinical adverse event (AE), abnormal laboratory test results or intercurrent illness which, in the opinion of the investigator, indicates that continued participation in the study is not in the best interest of the subject
- Pregnancy
- Termination of the study by BMS
- Loss of ability to freely provide consent through imprisonment or involuntary incarceration for treatment of either a psychiatric or physical (eg, infectious disease) illness
- Use of an investigational therapy other than study medication
- Subjects who meet the efficacy futility criteria (see Section 3.1.3)
- Laboratory/clinical criteria (see Section 3.5.1 and Section 4.3.1)
- Interruption of alfa-2a, Lambda or RBV > 14 days (see section 4.3.2)
- Criteria for potential drug-induced liver injury (DILI) (see Section 6.6)

The duration of post-treatment follow-up and study participation for all is provided in Sections 3.1.1 and 3.1.2, for Parts A and B, respectively. All subjects who discontinue must comply with protocol specified follow-up procedures as outlined in Section 5. The only

Approved v4.0 930056810 4.0

exception to this requirement is when a subject withdraws consent for all study procedures or loses the ability to consent freely (ie, is imprisoned or involuntarily incarcerated for the treatment of either a psychiatric or physical illness).

If a subject was withdrawn before completing the study, the reason for withdrawal must be entered on the appropriate case report form (CRF) page.

3.5.1 Laboratory/Clinical Criteria for Treatment Discontinuation

Laboratory/clinical criteria which may lead to discontinuation from treatment are provided in Section 4.3.1, along with any prior dose modification that may be required.

Clinical criteria that require treatment discontinuation take precedent over rules that allow for investigator discretion. If discontinuation of therapy is required, this must occur as soon as possible.

Subjects who meet the criteria for potential drug induced liver injury (DILI) should discontinue study treatment (see Section 6.6).

4 TREATMENTS

Study drugs include both Non-investigational (NIMP) and Investigational Medicinal Products (IMP) and can consist of the following:

- All products, active or placebo, being tested or used as a comparator in a clinical trial.
- Study required premedication, and
- Other drugs administered as part of the study that are critical to claims of efficacy (eg, backbone therapy, rescue medications)
- Diagnostic agents: (such as glucose for glucose challenge) given as part of the protocol requirements must also be included in the dosing data collection.

Approved v4.0 930056810 4.0

4.1 Study Treatments

Table 4.1: Product Description:

Product Description and Dosage Form	Potency	Primary Packaging (Volume)/ Label Type	Secondary Packaging (Qty) /Label Type	Appearance	Storage Conditions (per label)
Peginterferon lambda-1a (BMS-914143, Lambda)[a, b] solution for SC injection	180 mcg/0.45 mL	0.45 mL pre-filled syringe/ Open Label (Part A) Double Blind (Part B)	Outer Carton / Open Label (Part A) Double Blind (Part B)	Solution	2°C to 8°C (36°F - 46°F) Do not freeze. Protect from light.
Peginterferon alfa-2a (Pegasys®, alfa-2a) [a, b] solution for SC injection	180 mcg/0.5 mL	0.5 mL pre-filled syringe/ Double Blind (Part B)	Outer Carton / Double Blind (Part B)	Solution	2°C to 8°C (36°F - 46°F) Do not freeze. Protect from light.
Telaprevir (TVR) [a, c] Film Coated Tablet (US sourced)	375 mg	7 Blister Strips with 6 tablets per strip / Open Label (Part A and Part B)	Outer Carton 42 Tablets (7 blisters x 6 tablets)	A purple film-coated capsule-shaped tablet. Each tablet is debossed with the characters V375 on one side.	Store at 25ºC (77ºF). Excursions permitted to 15-30ºC (59-86ºF).
Telaprevir (TVR) [a, c] Film Coated Tablet (EU sourced)	375 mg	42 tablets per bottle / Open Label (Part B)	N/A	A yellow caplet-shaped film-coated tablet. Each tablet is marked with T375 on one side.	Store at15-30ºC (59-86ºF). Store in original container. Store in a tightly closed container to protect from moisture. Do not remove desiccant.
Ribavirin (RBV, Ribasphere) [a, c] Tablet	200 mg	168 tablets per bottle/ Open Label (Part A and Part B)	NA	Capsule-shaped, light-blue color, film-coated tablet	Store at 25°C (77°F). Excursions permitted between 15°C and 30°C (59°F to 86°F). Keep bottle tightly closed.

[a] The Investigator (or designee ie, study pharmacist) will dispense enough study medication, as assigned by IVRS to satisfy dosing requirements until the subject's next study medication dispensation visit.

Approved v4.0 930056810 4.0

[b] Lambda solution for injection and alfa-2a solution for injection will be supplied by Bristol-Myers Squibb Research and Development as a single use product only. The subject should inspect the pre-filled syringe visually for particulate matter and discoloration before administration. Such syringes should not be used and should be returned to the investigator.

[c] TVR and RBV will be supplied by Bristol-Myers Squibb Research and Development. TVR and RBV may be locally sourced if necessary.

Approved v4.0 930056810 4.0

4.1.1 Investigational Product

An investigational product, also known as investigational medicinal product in some regions, is defined a pharmaceutical form of an active substance or placebo being tested or used as a reference in a clinical study, including products already with a marketing authorization but used or assembled (formulated or packaged) differently than the authorized form, or used for an unauthorized indication, or when used to gain further information about the authorized form.

The investigational product should be stored in a secure area according to local regulations. It is the responsibility of the investigator to ensure that investigational product is only dispensed to study subjects. The investigational product must be dispensed only from official study sites by authorized personnel according to local regulations.

In this protocol, investigational product(s) is/are:

- Peginterferon lambda-1a (BMS-914143, Lambda)
- Peginterferon alfa-2a (Pegasys®, alfa-2a)
- Ribavirin (Ribasphere, RBV)
- Telaprevir (TVR)

For additional information on Lambda, please refer to the IB.[44] For additional information on alfa-2a, RBV and TVR, please refer to the local package inserts[45,46], Summary of Product Characteristics (SmPC)/reference label[47,48,49], or country specific reference label.

4.1.2 Non-investigational Product

Other medications used as support or escape medication for preventative, diagnostic, or therapeutic reasons, as components of the standard of care for a given diagnosis, may be considered as non-investigational products.

In this protocol, non-investigational product(s) is/are: Not applicable in this protocol.

4.1.3 Handling and Dispensing

The product storage manager should ensure that the study drug is stored in accordance with the environmental conditions (temperature, light, and humidity) as determined by BMS. If concerns regarding the quality or appearance of the study drug arise, the study drug should not be dispensed and contact BMS immediately.

Investigational product documentation must be maintained that includes all processes required to ensure drug is accurately administered. This includes documentation of drug storage, administration and, as applicable, storage temperatures, reconstitution, and use of required processes (eg, required diluents, administration sets).

Approved v 4.0 930056810 4.0

4.2 Method of Assigning Subject Identification

At the start of the screening period for Part A and Part B, the site staff will call the Interactive Voice Response System (IVRS) designated by the sponsor to register the patient and to obtain a Patient Identification Number (PID). The site staff will call the IVRS again once protocol eligibility criteria have been determined. For subjects who meet the protocol eligibility criteria during screening, the IVRS will register the subject to treatment. It is important that the investigative staff reconfirm the subject's willingness to continue in the study prior to calling the IVRS to register the subject to treatment. For subjects who do not meet the eligibility criteria, the IVRS will register them as screen failures.

Re-evaluation to confirm eligibility criteria within the 42 day screening period will be allowed. However, beyond this, subjects should be re-evaluated only after consultation with the study team.

Subjects with HCV GT-1b subtype will be capped at approximately 50%. Relapsers will be capped at approximately 20%. Subjects with compensated cirrhosis will be capped at approximately 10%.

The site staff will call the IVRS on Day 1, and at each visit mentioned in the Table 5.1B during treatment for the system to assign study medication.

At specific time points, the site staff will call the IVRS to determine if the subject should continue dosing based on the HCV RNA result (see Table 4.2 and Table 5.1B). The Central Laboratory or Sponsor designee will provide the subject's HCV RNA result to the IVRS.

Table 4.2: Criteria for Continuing or Discontinuing Study Medication

HCV RNA > 1000 IU/mL at Week 4	At Week 6, the IVRS will announce to discontinue all study medications
HCV RNA < LLOQ Target Not Detected at Week 4 and 12	At Week 16, the IVRS will announce to continue the assigned medications for up to 24 weeks
HCV RNA < LLOQ Target Detected or HCV RNA ≥ LLOQ at Week 4 or 12	At Week 16, the IVRS will announce to continue the assigned medications for up to 48 weeks
HCV RNA > 1000 IU/mL at Week 12	At Week 16, the IVRS will announce to discontinue all study medications
HCV RNA detected at Week 24	At Week 28, the IVRS will announce to discontinue all study medications
Virologic Breakthrough:<br>a. Confirmed > 1 $\log_{10}$ increase in HCV RNA over nadir<br>OR<br>b. Confirmed HCV RNA ≥ LLOQ after previously having an HCV RNA level of < LLOQ target detected or not detected while on treatment	If there is virologic breakthrough the IVRS will announce to discontinue all study medications. Confirm HCV RNA within 2 weeks.

Approved v 4.0 930056810 4.0

More detailed information regarding IVRS will be provided in a separate document.

4.3 Selection and Timing of Dose for Each Subject

The screening period for this study (Part A and Part B) is 42 days. Eligible subjects must be dosed within 42 days of the day they were screened. On Day 1, after all Day 1 procedures have been performed, eligible subjects will start study drugs. The first dose (Day 1) of Lambda or alfa-2a must occur in the office/clinic under medical supervision, and it should be administered prior to TVR.

A designated member of the study staff at the investigative site will be unblinded in Part B only and will dispense study medications to the subject depending upon treatment assignment.

Subjects and/or their caregivers will be trained by the unblinded site staff on subcutaneous (SC) administration of the Lambda or alfa-2a study medication. Subjects will be instructed to discuss any concerns regarding the dosage preparation or SC administration of Lambda or alfa-2a with only the unblinded study personnel. Subjects will be provided with written guidelines for drug storage and administration and safe handling (including discarding) of needles.

Selection and timing of dose for each subject are as follows (with the exceptions described below):

- **Lambda or alfa-2a**: All subjects will self-administer 180 μg injection SC once weekly.

  The following applies if a scheduled dose of Lambda/alfa-2a is missed:
  - The subject takes the Lambda/alfa-2a when the subject next remembers to take the dose and then either:
    - Resets the schedule of dosing and laboratory assessments to be 7 days later
      - ***OR***
    - The subject may re-dose again, after taking the missed dose, but no less than 5 days must elapse between sequential doses.

- **RBV**: All subjects will take RBV orally twice daily with food. For subjects weighing < 75 kg, the total dose is 1000 mg per day and for those weighing ≥ 75 kg, the dose is 1200 mg per day. Therefore, subjects should take either 400 mg (2 tablets for subjects < 75 kg) or 600 mg (3 tablets for subjects ≥ 75 kg) in the morning with food and 600 mg (3 tablets) in the evening with food.

  The following applies if a scheduled dose of RBV is missed:
  - If a subject accidentally misses a scheduled dose of RBV, the investigator should advise the subject according to the manufacturer's prescribing information for the medication.

Approved v4.0 930056810 4.0

- **TVR**: All subjects will take 750 mg orally three times a day approximately 7 - 9 hours apart (two 375 mg tablets three times per day approximately 7 - 9 hours apart);
  - Additional instructions for TVR:
    - ♦ Subjects must swallow the tablets whole (without chewing or breaking or dissolving them);
    - ♦ The tablets will be administered in the fed state, meaning that subjects should have food intake within 30 minutes before dose administration. Food should contain approximately 20 grams of fat. Subjects should be advised that fat content of the meal or snack is critical for the absorption of TVR. Examples of some foods that can be taken with TVR include: a bagel with cream cheese, 1/2 cup (120 mL) of nuts, 3 tablespoons (45 mL) peanut butter, 1 cup (240 mL) ice cream, 2 ounces (56 g) of American or cheddar cheese, 2 ounces (56 g) potato chips, or 1/2 cup (120 mL) trail mix.
    - ♦ If the subject accidentally misses a dose of TVR and the missed dose is remembered within 4 hours of the scheduled dose time, the dose should be taken as soon as possible. If the subject accidentally misses a dose of TVR and the missed dose is remembered more than 4 hours after the scheduled dose time, the dose should be skipped and the next dose taken at the appropriate time.

4.3.1 Dose Modifications

Dose modifications should be managed according to Table 4.3.1.1A, Table 4.3.1.1B, Table 4.3.1.1C, Table 4.3.1.1D, Table 4.3.1.1E, Table 4.3.1.1F, and Table 4.3.1.2. These tables are based on recommendations from the alfa-2a and RBV package inserts[45,46] and Lambda clinical data in HCV-infected subjects (EMERGE Study[44]) and have been modified as necessary for this study.

Dose modification decisions should be based on central laboratory results when possible. If local labs are used for dose modification decisions, central labs must also be collected. It is recommended that local labs be requested, in addition to routine central labs, in the event that the subject is clinically unwell or when a central lab result indicates a deterioration of lab parameters. In such cases it would be reasonable to use a local lab result (assuming these results are known before central lab results) to determine if dose modification is required. If the central lab results are available prior to, or at the same time as local lab results, dose modifications must be based on central lab results.

For subjects who experience depression or other neuropsychiatric disorders on study, the guidelines in Table 4.3.1.2 should be followed. Subjects with new onset or worsening depression on study should be treated with antidepressants at the discretion of the investigator. Subjects with other neuropsychiatric disorders on study should be managed at the discretion of the investigator.

Approved v4.0 930056810 4.0

4.3.1.1 Lambda and alfa-2a Dose Modifications

The specific doses of Lambda and alfa-2a to be administered in the event of dose modifications are provided for each treatment group in Table 4.3.1.1A.

Table 4.3.1.1A: Dose Reductions of Lambda or alfa-2a

Assigned Treatment	Starting Dose (µg)<br>Dose Line 4	First Dose Reduction (µg)<br>Dose Line 3	Second Dose Reduction (µg)<br>Dose Line 2
Lambda	180	120	80
Alfa-2a	180	135	90

Table 4.3.1.1B: Guidelines for Dose Modification of Pegylated Interferon (Lambda/alfa-2a) due to Adverse Events

Toxicity	Dose Modification	Additional Instructions
**Adverse Events**		
≥ Grade 3 AE considered related to study drug and considered clinically significant	Hold dose until ≤ Grade 1 or baseline value, then restart at first dose reduction level	Permanently discontinue if either of the following is true:<br>1. Event occurs or recurs when subject is receiving pegIFN after the second dose reduction<br>2. Event does not resolve within 14 days of the date that treatment was held (no more than 2 sequential doses can be held)

Table 4.3.1.1C: Guidelines for Dose Modification of Pegylated Interferon (Lambda/alfa-2a) due to Hematological Abnormalities

Toxicity	Dose Modification	Additional Instructions
**ANC**		
≥ 750/mm^3	Maintain dose	
≥ 500/mm^3 and < 750/mm^3	Reduce to first dose reduction level	Permanently discontinue if event occurs or recurs when subject is receiving pegIFN at the second dose reduction level
< 500/mm^3	Hold pegIFN treatment until ANC > 1000/mm^3, then restart pegIFN treatment at the 2nd dose reduction level	Permanently discontinue pegIFN and consult medical monitor if either of the following is true:<br>1. Event occurs or recurs when subject is receiving pegIFN at the 2nd reduced dose<br>2. Event does not resolve within 2 weeks of the date that treatment was withheld (no more than 2 sequential doses can be missed for an event)

Approved v4.0 930056810 4.0

**Table 4.3.1.1C: Guidelines for Dose Modification of Pegylated Interferon (Lambda/alfa-2a) due to Hematological Abnormalities**		
**Toxicity**	**Dose Modification**	**Additional Instructions**
**Platelets**		
< 50,000/mm^3	Reduce dos e of pegIFN to 2nd dose reduction level	Permanently discontinue if occurs when subject is receiving pegIFN at the 2nd dose reduction level
< 25,000/mm^3		Permanently discontinue study pegIFN

**Table 4.3.1.1D: Guidelines for Dose Modification of Pegylated Interferon (Lambda/alfa-2a) due to Liver Abnormalities**		
**Toxicity**	**Dose Modification**[a]	**Additional Instructions**
ALT or AST > 5-10 x ULN ***and*** > 3 x baseline value	Withhold pegIFN until ALT or AST ≤ 5 x ULN or ≤ 3 x baseline value, whichever comes first, and then, restart pegIFN at the 1st dose reduction level.<br>If recurs, withhold pegIFN until ≤ 5 x ULN or ≤ 3 x baseline value, then, restart pegIFN at the 2nd dose reduction level.	Permanently discontinue study drug and consult medical monitor if any of the following is true:<br>1. Event occurs or recurs when subject is receiving pegIFN at the 2nd reduced dose<br>2. Event does not resolve within 2 weeks of the date that pegIFN was withheld (no more than 2 sequential doses can be missed for an event).<br>3. There is evidence of hepatic decompensation.
ALT or AST > 10 x ULN, regardless of baseline values	Withhold pegIFN until ALT or AST ≤ 10 x ULN, then restart pegIFN at the 1st dose reduction level.<br>If recurs, withhold pegIFN until ALT or AST ≤ 10 x ULN, then restart pegIFN at the 2nd dose reduction level.	Permanently discontinue study drug and consult medical monitor if any of the following is true:<br>1. Event occurs or recurs when subject is receiving pegIFN at the 2nd reduced dose<br>2. Event does not resolve within 2 weeks of the date that pegIFN was withheld (no more than 2 sequential doses can be missed for an event).<br>3. There is evidence of hepatic decompensation
Total bilirubin > 2.5 x ULN ***and*** direct bilirubin > 50% of total bilirubin level, regardless of ALT values.	Withhold pegIFN until total bilirubin ≤ 1.5 x ULN, then restart pegIFN at the 1st dose reduction level.<br>If recurs, withhold pegIFN until total bilirubin ≤ 1.5 x ULN, then restart pegIFN at the 2nd lower dose reduction level.	Permanently discontinue pegIFN and consult medical monitor if any of the following is true:<br>1. Event occurs or recurs when subject is receiving pegIFN at the 2nd reduced dose<br>2. Event does not resolve within 2 weeks of the date that treatment was withheld (no more than 2 sequential doses can be missed for an event).<br>3. There is evidence of hepatic decompensation
Child-Pugh Score (CP) > 6.		Permanently discontinue pegIFN.<br>Exception: If CP = 7 and is due to isolated hyperbilirubinemia only, discontinuation of study medication is not required.

Approved v4.0 930056810 4.0

[a] In general, the dose modification guidelines for hepatic lab abnormalities should be followed. However the Invesgtigator has the discretion to dose reduce interferon without withholding dosing, provided the subject does not have Grade 3 or 4 symptoms and is clinically stable without evidence of hepatic decompensation. Grade 4 lab abnormalities (ALT/AST > 10 x ULN or total bilirubin > 5 x ULN) should always be managed with a dose interruption followed by a dose reduction.

- Any subject who has a ≥ 2 fold increase in AST/ALT or any increase in bilirubin above upper limit of normal should have an additional visit within 2 weeks for repeat laboratory tests.
- When subjects have clinical jaundice or evidence of impairment of liver function which require dose modification, subjects must be monitored no less than weekly to ensure improvement and will also have a thorough clinical evaluation.
- Clinical jaundice is considered to be an important medical event for this study and should be reported as an SAE (see Section 6.1.1. for reporting details).
- When subjects have clinical signs of liver abnormalities eg, clinical jaundice, dose must be held until liver laboratory results are known.
- If it is uncertain whether a subject meets a drug discontinuation criteria, please contact study Medical Monitor to discuss the case, prior to discontinuation.
- Subjects who meet criteria for treatment discontinuation based on impaired liver function, should also have a clinical work-up which includes consideration of the following:
  - autoimmune markers (ANA, anti-SMA, anti-LKM1)
  - serologies for hepatitis A, hepatitis, B, hepatitis E, herpes simplex virus (HSV), Epsteinbarr-virus (EBV) and cytomegalovirus (CMV)
  - detailed medical history including concomitant medication use (including herbal or over the counter medications) and drug and alcohol intake
  - early consultation with a hepatologist should be considered (if not already being managed by a hepatologist)
  - Imaging studies for a possible extrahepatic cholestasis (ie, ultrasound)
  - liver biopsy with light and electron microscopic assessment
  - toxicology screening and blood alcohol monitoring

Initial liver-related laboratory abnormalities should be confirmed in 3 - 5 days prior to the reporting of a potential drug induced liver injury (DILI) event and discussed with the sponsor. All confirmed occurrences of potential DILIs, meeting the defined criteria (see Section 6.6), must be reported as SAEs (see Section 6.1.1. for reporting details). Subjects who meet criteria for DILI should be strongly considered for liver biopsy, provided clinical parameters do not contraindicate this.

Approved v4.0 930056810 4.0

Table 4.3.1.1E: Guidelines for Dose Modification of Lambda or alfa-2a due to Renal Impairment

Toxicity	Dose Modification	Additional Instructions
30-50 mL/min	None	
30 mL/min	Reduce to first dose reduction level	
Hemodialysis	Reduce to first dose reduction level	

Table 4.3.1.1F: Guidelines for Dose Modification of Lambda or alfa-2a due to New Ocular Symptoms

Toxicity	Dose Modification	Additional Instructions
New clinically significant decrease or loss of vision or other clinically significant ocular sign or symptom	Interrupt therapy and obtain complete eye examination performed by an ophthalmologist. Discuss further management with the medical monitor prior to restarting therapy.	

4.3.1.2 Depression Monitoring and Dose Modifications

In this study, subjects will be monitored real-time for signs and symptoms of depression using Patient Health Questionnaire (PHQ-9) starting at Day 1 and during all scheduled visits as indicated in Section 5.1. Subjects with treatment-emerging depression (PHQ-9 $\geq$ 15) or who respond positively to suicidal ideation will be further evaluated for suicidal behavior with the Columbia Suicide Severity Rating Scale (C-SSRS). All identified suicidal cases will be referred to a mental health professional for management. In addition to the close monitoring, all cases of clinically significant treatment-emergent depression will be managed with dose modification or discontinuation of the study drug and any suicidal subjects will discontinue study therapy.

For subjects who experience depression on study, the guidelines in Table 4.3.1.2 should be followed. Subjects with new onset or worsening depression on study should be prescribed antidepressants, including initiation of new medication or increase in dose, adjusted at the discretion of the clinical investigator. Subjects with other neuropsychiatric disorders on study should be managed at the discretion of the investigator.

Approved v4.0 930056810 4.0

Table 4.3.1.2: Guidelines for Dose Modification of Lambda/alfa-2a due to Depression					
	**Initial management (4 - 8 weeks)**		**Depression**		
**Depression Severity**	**Dose Modification**	**Visit Schedule**	**Remains Stable**	**Improves**	**Worsens**
Mild	No change	Evaluate once weekly by visit and/or phone	Continue weekly visit schedule	Resume normal visit schedule	(See moderate or severe depression
Moderate	Decrease dose to 1st dose reduction level (in some cases dose reduction to 2nd dose reduction level may be needed)	Evaluate once weekly (office visit at least once every other week)	Consider psychiatric consultation. Continue reduced dosing	If symptoms improve and are stable for 4 weeks, may resume normal visit schedule. Continue with reduced dosing	(See severe depression)
Severe	Discontinue Lambda or alfa-2a permanently	Obtain immediate psychiatric consultation	Psychiatric therapy necessary		

4.3.1.3 Ribavirin Dose Modifications

RBV must not be administered as monotherapy. Dose modifications of RBV should be made in accordance with the RBV package insert.[46] If Grade 3 or higher adverse events or clinically significant laboratory abnormalities develop during RBV treatment, the dose of RBV should be modified or held, if appropriate, until the adverse reactions abate. Subjects who require dose modifications for laboratory abnormalities should have follow-up laboratory assessments performed at least weekly until the abnormality has stabilized or returned to baseline. If intolerance persists after dose adjustment, RBV therapy should be discontinued. If RBV is permanently discontinued for any reason, Lambda or alfa-2a and TVR must also be discontinued. Subjects with a history of cardiac disease should be appropriately monitored during therapy and if there is any deterioration of cardiovascular status, therapy should be stopped. Guidelines for dose modifications and discontinuation of RBV for management of changes in hemoglobin and creatinine clearance are provided in Table 4.3.1.3.

Table 4.3.1.3: Ribavirin Dose Modification Guidelines		
**Laboratory Value/Clinical Criteria:**	**Dose Modification:**	**Additional Instructions:**
**<u>Hemoglobin:</u>**		
> 8.5 to ≤ 10 g/dL	Reduce to 600 mg daily	Take 1 tablet in the morning and 2 tablets in the evening

Approved v 4.0 930056810 4.0

**Table 4.3.1.3: Ribavirin Dose Modification Guidelines**		
**Laboratory Value/Clinical Criteria:**	**Dose Modification:**	**Additional Instructions:**
≤ 8.5 g/dL	RBV should be interrupted until hemoglobin values return to more than 10 g/dL	When values return to more than 10 g/dL, restart at 600 mg daily and further increase dose to 800 mg daily based on investigator discretion
**Creatinine Clearance:**		
30-50 mL/min	Alternating doses, 200 mg and 400 mg every other day;	
30 mL/min	200 mg daily	
Hemodialysis	200 mg daily	

4.3.1.4 Telaprevir

TVR should be administered according to local label indications. TVR must not be adjusted or administered as monotherapy. Specific guidance for TVR anemia, rash management, or serious skin reactions is provided in Sections 5.3.5, 5.3.6 and 5.3.7 of the protocol.

4.3.2 Dose Interruptions

Should drug interruption/suspension be necessary for any laboratory abnormality or AE, the following rules must be applied, and the central medical monitor informed (dose interruptions due to lack of compliance that deviate from these rules must be discussed with the medical monitor to determine the proper course of action):

- No more than 2 sequential doses of Lambda or alfa-2a may be interrupted and if not re-initiated within 14 days from the day of the interruption, the subject must discontinue all study drug treatment and enter follow-up.
- If the Lambda or alfa-2a dose is not administered:
  - RBV must be interrupted the same day as the missed Lambda or alfa-2a dose and, if not re-initiated with Lambda or alfa-2a, within 14 days of the interruption, the subject must discontinue all study drug treatment and enter follow-up.
  - TVR must be interrupted. If TVR is interrupted for an adverse event that is thought to be related to TVR such as rash or anemia, it must not be restarted. Subjects may continue treatment with Lambda or alfa-2a and RBV for the duration of their assigned treatment regimen.
- If RBV is discontinued or interrupted for more than 14 days, the subject must discontinue all study drug treatment and enter follow-up.

Table 4.3.2 indicates permissible regimens in the event that interruption of individual study medication(s) is necessary.

Approved v4.0 930056810 4.0

Table 4.3.2: Permissible Regimens for Up To 14 Days in the Event of Treatment Interruption

Potential Treatment Regimens During a Drug Interruption	
Lambda or alfa-2a monotherapy	Yes
RBV monotherapy	No
TVR monotherapy	No
Lambda or alfa-2a + RBV[a]	Yes
RBV + TVR	No
Lambda or alfa-2a + TVR	Yes

[a] May be continued beyond 14 days if TVR must be discontinued.

4.4 Blinding/Unblinding

Blinding is critical to the integrity of this clinical study. However, in the event of a medical emergency or pregnancy in an individual subject, **in which knowledge of the investigational product is critical to the subject's management,** the blind for that subject may be broken by the treating physician.

Before breaking the blind of an individual subject's treatment, the investigator determines whether that the information is necessary, ie, that it will alter the subject's immediate management. In many cases, particularly when the emergency is clearly not investigational product-related, the problem may be properly addressed by assuming that the subject is receiving active product without the need for unblinding.

In cases of accidental unblinding, contact the Medical Monitor and ensure every attempt to preserve the blind is made.

Part A of this study is open-label as treatment blinding is not required. In Part B of this study, treatment assignment will be site and subject blinded for the entire duration of the study.

A designated member of the study staff at the investigative site will be unblinded in Part B only and will dispense study medications to the subject depending upon treatment assignment. In addition, only necessary personnel at the Sponsor not directly involved in the assessment of safety in the study will be unblinded to HCV RNA results for on-treatment visits. Investigative staff will receive screening HCV RNA results for the purpose of randomization, but will not receive HCV RNA results during the study treatment or IL28B results. Sites will receive HCV RNA results for subjects at the end of study treatment and during follow-up. The virologic breakthrough and treatment futility criteria will be programmed through IVRS in order to notify sites of the subjects meeting these criteria, for further management and to maintain the integrity of the study.

Approved v4.0 930056810 4.0

4.5 Treatment Compliance

Assessment of study medication use will be performed at each study visit. The subject should be instructed to bring all unused and empty bottles of RBV and TVR and empty containers that the Lambda/alfa-2a syringes are provided in to each visit. The dates, number of RBV and TVR tablets and Lambda/alfa-2a containers dispensed and returned must be recorded on the drug accountability form maintained on-site. Opened bottles of RBV and TVR study medication should be returned to the subject so that dosing may continue from the in-use bottle until it has been emptied. Lambda/alfa-2a syringes should be discarded in the provided sharps container after their use. The empty containers that the syringes are provided in must be returned to the site to ensure drug accountability is completed. Subjects will be instructed to record dosing in a diary, which will be reviewed at each visit to confirm treatment compliance. The site staff should discuss with the subject if there are discrepancies between the diary and the drug accountability form to reconcile actual dosing at each visit.

4.6 Destruction and Return of Study Drug

4.6.1 Destruction of Study Drug

For this study, study drugs (those supplied by BMS or sourced by the investigator) such as partially used study drug containers, vials and syringes may be destroyed on site.

Any unused study drugs can only be destroyed after being inspected and reconciled by the responsible BMS Study Monitor unless study drug containers must be immediately destroyed as required for safety, or to meet local regulations (eg, cytotoxics or biologics).

On-site destruction is allowed provided the following minimal standards are met:

- On-site disposal practices must not expose humans to risks from the drug.
- On-site disposal practices and procedures are in agreement with applicable laws and regulations, including any special requirements for controlled or hazardous substances.
- Written procedures for on-site disposal are available and followed. The procedures must be filed with the site's SOPs and a copy provided to BMS upon request.
- Records are maintained that allow for traceability of each container, including the date disposed of, quantity disposed, and identification of the person disposing the containers. The method of disposal, i.e. incinerator, licensed sanitary landfill, or licensed waste disposal vendor must be documented.
- Accountability and disposal records are complete, up-to-date, and available for the Monitor to review throughout the clinical trial period.

If conditions for destruction cannot be met the responsible BMS Study Monitor will make arrangements for return of study drug.

It is the investigator's responsibility to arrange for disposal of all empty containers, provided that procedures for proper disposal have been established according to applicable federal, state, local,

Approved v4.0 930056810 4.0

and institutional guidelines and procedures, and provided that appropriate records of disposal are kept.

4.6.2 Return of Study Drug

If study drug will not be destroyed upon completion or termination of the study, all unused and/or partially used study drug that was supplied by BMS must be returned to BMS. The return of study drug will be arranged by the responsible BMS Study Monitor.

It is the investigator's responsibility to arrange for disposal of all empty containers, provided that procedures for proper disposal have been established according to applicable federal, state, local, and institutional guidelines and procedures, and provided that appropriate records of disposal are kept.

Approved v4.0 930056810 4.0

5 STUDY ASSESSMENTS AND PROCEDURES

5.1 Flow Chart/Time and Events Schedule

Table 5.1A: Screening Procedural Outline (AI452020)

Procedure	Screening Visit	Notes
**Eligibility Assessments**		
Informed Consent	X	
Inclusion/Exclusion Criteria	X	
Medical History	X	
HCV Genotype	X	Required at randomization for stratification
IL28B SNP (rs12979860)	X	Required at randomization for stratification
HBV, HCV, HIV Serology	X	
Baseline Liver Biopsy or Fibroscan®	X	Biopsy results obtained within 3 years of randomization (unless cirrhotic); H and E stained slide if available. Fibroscan® within 1 year prior to randomization. Data for cirrhotic subjects must be evaluated by the BMS Central Medical Monitor prior to randomization.
AFP	X	If AFP is ≥ 50 but < 100 ng/mL at screening, absence of a mass must be demonstrated by US/CT/MRI imaging within the screening period
**Safety Assessments**		
Full Physical Examination	X	
ECG, Single 12 Lead	X	
Vital Signs/Weight/Height	X	
Eye exam (including retinal exam)	X	The eye (including retinal) exam may be done by the investigator; however if a subject has underlying diabetes, hypertension or history of preexisting eye disease, a complete dilated eye exam should be done by an eye care professional.
Laboratory Tests	X	
Pregnancy Test	X	For WOCBP only; positive results to be confirmed by serum

Approved v4.0 930056810 4.0

Table 5.1A: Screening Procedural Outline (AI452020)		
**Procedure**	**Screening Visit**	**Notes**
Concomitant Medication Use	X	
Assessment of Signs and Symptoms	X	
Serious Adverse Events Assessment	X	Report SAEs that occur after informed consent is obtained
**Efficacy Assessment**		
HCV RNA (serum sample)	X	
HCV RNA back-Back up sample	X	To be stored on site and sent to central lab in separate shipment to minimize chance of loss due to delay or damage of samples

Approved v4.0 930056810 4.0

Approved v4.0 930056810 4.0

Table 5.1B: Procedural Outline On Treatment (AI452020)

Procedure	Baseline Day 1 (within 42 days of the Screening Visit)	During Treatment (± 3 days)											End of Treatment Wk 24 or 48 or early discontinuation (± 3 days)	Notes
		Wk 1	Wk 2	Wk 4	Wk 5	Wk 6	Wk 8	Wk 10	Wk 12	Wk 16	Wk 20	Wks 24, 28, 32, 36, 40, & 44 for subjects with detectable HCV RNA at Weeks 4 or 12		
		D 8	D 15	D 29	D 36	D 43	D 57	D 71	D 85	D 113	D 141			
**Eligibility Assessments**														
Inclusion/Exclusion Criteria	X													Prior to randomization to ensure eligibility criteria are met
**Safety Assessments**														
Targeted Physical Examination	X	X	X	X		X	X	X	X	X	X	X	X	On treatment targeted exam only at the discretion of Investigator.
Vital Signs/Weight	X	X	X	X		X	X	X	X	X	X	X	X	
ECG, 12 Lead	X			X								Week 24	Week 24	
Concomitant Medication Use	X	X	X	X		X	X	X	X	X	X	X	X	
Serious Adverse Event/ Adverse Event Assessment	X	X	X	X		X	X	X	X	X	X	X	X	Day 1 AE assessment occurs after study drug administration
Laboratory Tests	X	X	X	X		X	X	X	X	X	X	X	X	
Pregnancy Test, WOCBP	X			X			X		X	X	X	X	X	Urine pregnancy test performed on Day 1, and then every 4 weeks while on treatment for WOCBP; positive results to be confirmed by serum.

Approved v4.0 930056810 4.0

Table 5.1B: Procedural Outline On Treatment (AI452020)

Procedure	Baseline Day 1 (within 42 days of the Screening Visit)	During Treatment (± 3 days)											End of Treatment Wk 24 or 48 or early discontinuation (± 3 days)	Notes
		Wk 1	Wk 2	Wk 4	Wk 5	Wk 6	Wk 8	Wk 10	Wk 12	Wk 16	Wk 20	Wks 24, 28, 32, 36, 40, & 44 for subjects with detectable HCV RNA at Weeks 4 or 12		
		D 8	D 15	D 29	D 36	D 43	D 57	D 71	D 85	D 113	D 141			
**Efficacy Assessments**														
Serum Specimen for HCV RNA	X	X	X	X		X	X	X	X	X	X	Weeks 24 & 36	X	Samples to be drawn pre-dose
Plasma Specimen for HCV Resistance	X	X	X	X			X		X	X		Week 24	X	Samples to be drawn pre-dose
HCV RNA Back up Samples	X	X	X	X		X	X	X	X	X	X	Weeks 24 & 36	X	To be stored on-site at -70°C and sent to central lab in separate shipment to minimize change of loss due to delay or damage of sample
**Other Assessments**														
PK trough Samples for Lambda, alfa-2a & TVR	X		X	X					X			Week 24		See Table 5.5A for timing. All Samples are collected pre-dose
Intensive PK Samples or Lambda, alfa-2a-& TVR	X		X	D29 -35	X				X			Week 24		Please refer to Table 5.5B for intensive PK sampling time schedule
Blood sample for analysis of exploratory Genetic Biomarkers	X													Preferable at baseline, but may be obtained at any time after randomization

Table 5.1B: Procedural Outline On Treatment (AI452020)

Procedure	Baseline Day 1 (within 42 days of the Screening Visit)	During Treatment (± 3 days)											End of Treatment Wk 24 or 48 or early discontinuation (± 3 days)	Notes
		Wk 1	Wk 2	Wk 4	Wk 5	Wk 6	Wk 8	Wk 10	Wk 12	Wk 16	Wk 20	Wks 24, 28, 32, 36, 40, & 44 for subjects with detectable HCV RNA at Weeks 4 or 12		
		D 8	D 15	D 29	D 36	D 43	D 57	D 71	D 85	D 113	D 141			
Blood sample for potential bridging of rs12979860 SNP (for storage)	X													For Part A
Serum biomarkers for host immune response	X			X					X					
Gene expression biomarkers for host immune response	X			X					X					
Serum sample for immunogenicity testing	X		X	X					X			Week 24	X	Samples to be drawn pre-dose.
Hepatitis Physical Symptom Diary (HPSS-D)	X			X			X		X			Weeks 24, 32 & 40	X	Completed at baseline before treatment is initiated and for the next six days after baseline. Subjects will complete the diary at home at each time point indicated for seven days based on language availability.
Fatigue Severity Scale (FSS)	X			X					X			Week 24	X	To be completed by the subject at the site based on language availability.

Approved v4.0 930056810 4.0

Approved v4.0 930056810 4.0

Table 5.1B: Procedural Outline On Treatment (AI452020)

Procedure	Baseline Day 1 (within 42 days of the Screening Visit)	During Treatment (± 3 days)											End of Treatment Wk 24 or 48 or early discontinuation (± 3 days)	Notes
		Wk 1	Wk 2	Wk 4	Wk 5	Wk 6	Wk 8	Wk 10	Wk 12	Wk 16	Wk 20	Wks 24, 28, 32, 36, 40, & 44 for subjects with detectable HCV RNA at Weeks 4 or 12		
		D 8	D 15	D 29	D 36	D 43	D 57	D 71	D 85	D 113	D 141			
EQ-5D	X			X			X		X			Week 24	X	To be completed by the subject at the site based on language availability.
PHQ-9	X	X	X	X		X	X	X	X	X	X	X	X	
Columbia Suicide Severity Rating Scale (C-SSRS)	X	X	X	X		X	X	X	X	X	X	X	X	Only for subjects with PHQ-9 ≥ 15
**Clinical Drug Supplies**														
Call IVRS to register eligible subjects for treatment	X													
Call IVRS to determine if subject should continue dosing based on HCV RNA result				X		X	X	X	X	X	X	Week 28		Part B only (HCV RNA results are blinded)
Call IVRS to Dispense Lambda (Part A)	X		X	X		X	X		X	X	X	X		
Call IVRS to Dispense Lambda or alfa-2a (Part B)	X		X	X		X	X		X	X	X	X		
Call IVRS to Dispense RBV	X			X			X		X	X	X	X		

Approved v4.0 930056810 4.0

Table 5.1B: Procedural Outline On Treatment (AI452020)

Procedure	Baseline Day 1 (within 42 days of the Screening Visit)	During Treatment (± 3 days)											End of Treatment Wk 24 or 48 or early discontinuation (± 3 days)	Notes
		Wk 1	Wk 2	Wk 4	Wk 5	Wk 6	Wk 8	Wk 10	Wk 12	Wk 16	Wk 20	Wks 24, 28, 32, 36, 40, & 44 for subjects with detectable HCV RNA at Weeks 4 or 12		
		D 8	D 15	D 29	D 36	D 43	D 57	D 71	D 85	D 113	D 141			
Call IVRS to Dispense TVR	X		X	X		X	X							
Assessment of Study Medication Use		X	X	X		X	X	X	X	X	X	X	X	

Approved v4.0 930056810 4.0

Table 5.1C: Procedural Outline Off Treatment (AI452020)						
**Procedure**	**Post-Treatment Follow-up (± 5 days)**					**Notes**
	Wk 4	Wk 12	Wk 24	Wk 36	Wk 48	
Safety Assessments						
Targeted Physical Examination	X	X	X	X	X	
Vital Signs/Weight	X	X	X	X	X	
Concomitant Medication Use	X	X	X	X	X	After post-treatment Week 4, assess for anti-HCV medications only. Once alternative HCV medications initiated, subject should discontinue from post-treatment follow-up phase.
Serious Adverse Event Assessment	X					SAEs will be collected after Week 4 post-treatment if the investigator believes it is related to the study drug or protocol specified procedures.
Adverse Event Assessment	X	X	X			
Laboratory Tests	X	X	X	X	X	See Section 5.3.1
Urine Pregnancy Test, WOCBP	X	X	X			Pregnancy testing is required every 4 weeks following discontinuation of RBV for 6 months post-treatment, or the duration specified in the label used for RBV in that country, whichever is longer. Home pregnancy testing may be performed on post treatment Weeks 8, 16, and 20; however any positive result must be verified by serum pregnancy test. Telephone contacts are required to obtain results for subjects who perform post-treatment at-home pregnancy testing and subjects will be instructed to record test results in a pregnancy test result log.
Efficacy Assessments						
Serum Specimen for HCV RNA	X	X	X	X	X	
Plasma Specimen for HCV Resistance	X	X	X	X	X	

Approved v4.0 930056810 4.0

Table 5.1C: Procedural Outline Off Treatment (AI452020)

Procedure	Post-Treatment Follow-up (± 5 days)					Notes
	Wk 4	Wk 12	Wk 24	Wk 36	Wk 48	
HCV RNA Back up Samples	X	X	X	X	X	To be stored on-site at -70°C and sent to central lab in separate shipment to minimize change of loss due to delay or damage of sample.
Other Assessments						
Serum sample for immunogenicity		X	X	X	X	
Hepatitis Physical Symptom Severity Diary (HPSS)	X	X	X			To be completed by the subject at the site before study treatment is administered and for the next 6 consecutive days at home based on language availability.
Fatigue Severity Scale (FSS)		X	X		X	To be completed by the subject at the site based on language availability.
EQ-5D		X	X		X	To be completed by the subject at the site based on language availability.
PHQ-9	X	X	X	X	X	
Columbia Suicide Severity Rating Scale (C-SSRS)	X	X	X	X	X	Only for subjects with PHQ-9 ≥ 15

5.2 Study Materials

The site will provide all required materials for the tests performed locally (ie, relevant clinical laboratory tests). The site will have available a well calibrated scale for recording body weight, a calibrated sphygmomanometer and thermometer for vital sign assessments. The site will have a monitored refrigerator, and freezer (-70°C or below), as well as containers and dry ice for shipment and storage of blood samples. A refrigerated centrifuge is also recommended. The site will provide all materials required for accurate source documentation of study activities and for housing the subjects during the study.

BMS will provide a BMS-approved protocol and any amendments or administrative letters (if required). Case report forms (electronic or hard copy) will be provided by BMS. The Central Laboratory will provide a laboratory manual and labels and tubes for the collection of all required materials for the clinical laboratory tests performed by the Central Laboratory. Investigational products will be supplied by BMS. BMS will also provide the Investigator Brochure, and the IVRS manual and other study related supplies (eg, sharps container, gel packs, etc) to be used by subjects. Dosing diaries will be provided by BMS.

5.3 Safety Assessments

Only data for the procedures and assessments specified in this protocol should be submitted to BMS on a case report form. Additional procedures and assessments may be performed as part of the subject's standard medical care; however, data for these assessments should remain in the subject's medical record and should not be provided to BMS, unless specifically requested from the sponsor.

5.3.1 Laboratory Assessments

The following assessments listed in Table 5.3.1 will be analyzed by a central or other BMS-specified laboratory:

Table 5.3.1: Laboratory Assessments

	**Screening (Outlined in Table 5.1A)**	**Study Visits (Outlined in Table 5.1B and Table 5.1C)**
Hematology:		
Hemoglobin	X	X
White Blood Cell Count with differential	X	X
ANC (neutrophils plus bands)	X	X
ANA	X	X
INR	X	X
PTT	X	X
Platelets	X	X

Approved v4.0 930056810 4.0

Table 5.3.1: Laboratory Assessments

	**Screening (Outlined in Table 5.1A)**	**Study Visits (Outlined in Table 5.1B and Table 5.1C)**
Hematocrit	X	X
Chemistry:		
Albumin	X	X
Total Protein	X	X
Aspartate aminotransferase (AST) (reflex to creatinine phosphokinase [CPK] for ≥ Grade 1 AST elevation without ALT elevation)	X	X
Alanine aminotransferase (ALT)	X	X
Total bilirubin	X	X
Direct bilirubin	X	X
Alkaline phosphatase	X	X
Lactate dehydrogenase (LDH)	X	X
Creatinine	X	X
Creatinine Clearance	X	X
Creatinine phosphokinase (CPK)		For AST elevation Grade 1 or above without ALT elevation
Amylase	X	X
Lipase	X	X
γ-glutamyl transferase (GGT)	X	X
Thyroid stimulating hormone (TSH)	X	Week 12 then every 12 weeks while on treatment<br>Post-treatment Week 4
Thyroid Function tests (T4 Free)	X	Week 12 then every 12 weeks while on treatment<br>Post-treatment Week 4
Electrolytes (Sodium, Bicarbonate, Potassium, Chloride)	X	X
Blood Urea Nitrogen (BUN)	X	
Glucose (random)	X	
HbA1c	X	
FSH	X	
Calcium	X	
Phosphate	X	
Uric Acid	X	X
Alpha Fetoprotein	X	

Approved v4.0 930056810 4.0

Table 5.3.1: Laboratory Assessments

	Screening (Outlined in Table 5.1A)	Study Visits (Outlined in Table 5.1B and Table 5.1C)
Triglycerides	X	Day 1, Weeks 4, 12, then every 12 weeks while on treatment
Lipids profile (cholesterol, HDL, LDL, apo B, apo A-I, apo E, apo C-III)	X	Day 1, Weeks 4, 12, then every 12 weeks while on treatment
Urine pregnancy test, if positive reflex to serum	In WOCBP	In WOCBP
Serum Pregnancy Testing	Only if urine pregnancy test is positive	Only if urine pregnancy test is positive
HCV RNA	X	X
HCV resistance specimen for storage		X
HIV-1 and -2 Ab	X	
Hepatitis B sAg	X	
HCV GT	X	
Anti-HCV antibody	X	

Results of all laboratory tests required by this protocol must be provided to BMS, either recorded on the laboratory pages of the CRF or by another mechanism as agreed upon between the investigator and BMS (e.g., provided electronically). If the units of a test result differ from those printed on the CRF, the recorded laboratory values must specify the correct units. Refer to Section 6.3 for reporting laboratory test abnormalities.

Pregnancy testing must be completed for WOCBP at post-treatment follow-up Weeks 4, 8, 12, 16, 20, and 24 (see Table 5.1C). Pregnancy testing may be performed at home if an in-office visit is not otherwise required. Telephone contacts are required to obtain results for all subjects who perform post-treatment at-home pregnancy testing. Although testing may be performed with home pregnancy testing kits, any positive result must be confirmed by serum pregnancy testing at study site.

The Roche COBAS® TaqMan HCV Test v.2.0 (lower limit of quantitation [LLOQ] = 25 IU/mL for HCV GT-1 subtype at the time of protocol development) will be used to measure HCV RNA levels. The VERSANT HCV genotype 2.0 assay (LIPA) will be used for genotype/subtype assessments. In cases where the VERSANT HCV genotype 2.0 assay (LiPA) provides unavailable or inconclusive results for HCV genotype/subtype, additional analysis may be performed to determine HCV genotype/subtype by sequencing of the NS5B region of HCV (analysis to be performed by Janssen Diagnostics). Serum is utilized for HCV RNA viral load testing, whereas plasma is used for the HCV resistance testing. This information is provided in the central laboratory manual that is made available to all participating study sites.

Approved v4.0 930056810 4.0

Further details of laboratory sample collection, processing and shipping will be provided to the site in the procedure manual.

5.3.2 Adverse Events Assessments

Subjects will be closely monitored throughout the study for AEs. AEs should be reported throughout the on-treatment period and through post-treatment follow-up Week 24 (see Table 5.1B and Table 5.1C). Subjects who discontinue assigned therapy prematurely should proceed to the post-treatment follow-up visits as indicated in Table 5.1C. All study drug-related AEs must be followed until resolution or stabilization. SAEs should be reported through screening, the on-treatment period and through post-treatment follow-up Week 4. SAEs will also be collected after Week 4 post-treatment if the investigator believes it is related to the study drug or protocol specified procedures.

5.3.3 Electrocardiogram

A 12-lead ECG performed while the subject is resting in a supine position will be recorded at study visits outlined in Table 5.1A and Table 5.1B. The ECG should be recorded after the subject has been supine for at least 5 minutes.

5.3.4 Vital Signs and Physical Examinations

Vital signs (seated blood pressure and heart rate), weight, and physical measurements and examinations must be performed at study visits outlined in Table 5.1A, Table 5.1B, and Table 5.1C. Physical measurements including height and weight for calculation of BMI will be performed at screening.

All subjects should be evaluated by qualified study site personnel at every visit, capable of making proper safety assessments based on the clinical history obtained from the subject.

A full physical examination will be performed at the Screening visit. A targeted physical exam should be performed at Day 1, during on-treatment visits, and the post-treatment Week 4 visit, when deemed necessary by the investigator when safety or other assessments warrant additional physical examination. A targeted physical examination may be performed by a qualified professional guided by the examiner’s observations and/or subject complaints on new or changed conditions, symptoms or concerns. Targeted physical exam includes assessment of heart, lung and abdomen.

An eye exam (including retinal exam) will be done at screening. This may be done by the investigator; however if a subject has underlying diabetes, hypertension or history of preexisting eye disease, a complete dilated eye exam should be done by an eye care professional.

5.3.5 Anemia Management for TVR Treated Patients

For the management of anemia, RBV dose reductions should be made in accordance with the product labeling for RBV. If RBV is discontinued for the management of anemia, TVR must also be discontinued. The use of erythropoetic growth factors is discouraged and will not be reimbursed by the Sponsor. Erythropoetic growth factors may be used at the discretion of the

Approved v4.0 930056810 4.0

Investigator in addition to RBV dose reductions during the first 12 weeks of treatment. The use of these growth factors is prohibited after the first 12 weeks of treatment. TVR dose reductions are prohibited and once TVR treatment is discontinued, it should not be reinitiated.

5.3.6 Rash Management

The rash management plan is adapted from the ADVANCE protocol.[28]

5.3.6.1 Rash or Rash-Like Events of Special Interest

All skin reactions involving rash or rash-like events that occur during the study and which meet any of the following 3 criteria will be considered an 'Event of Special Interest' (EOSI):

- Permanent discontinuation of any or all study drugs due to rash
- Grade 3 or 4 rash
- Rash which meets the criteria to be an SAE (serious adverse event)

All EOSIs should be recorded on a specific CRF module, and reported to the central Medical Monitor within 24 hours. For subjects on TVR treatment, rash management should follow guidelines specified in Section 5.3.6.4.

5.3.6.2 Rash Assessment

The grade and severity rating of rash events should be assigned using the following criteria:

Grade 1, mild: Rash is defined as a localized skin eruption and/or a skin eruption with a limited distribution (eg, up to several isolated sites on the body), with or without associated pruritis. A mild rash will have no target lesions, no signs of systemic involvement and no involvement of mucous membranes or signs of epidermal detachment.

Grade 2, moderate: Rash is defined as a diffuse skin eruption involving up to approximately 50% of the body surface; with or without superficial skin peeling, pruritis, or mucous membranes involvement with no ulceration. A moderate rash will have no signs of target lesions or epidermal detachment. A moderate rash may have systemic symptoms which are mild and /or limited.

Grade 3, severe: Rash is defined as a generalized rash involving over 50% of the body surface; or rash presenting with any of the following characteristics

- Rash with vesicles or bullae
- Superficial ulceration of mucous membranes
- Epidermal detachment (full thickness epidermal necrosis and separation of epidermis from underlying dermis)
- Atypical or typical target lesions
- Palpable purpura/non-blanching erythema

Approved v4.0 930056810 4.0

Rash with appearance of significant systemic signs or symptoms that are new and are considered related to the onset and/or progression of rash should be considered to be Grade 3.

In addition to events meeting the criteria above, any events of Stevens-Johnson Syndrome (SJS), Toxic Epidermal Necrolysis (TEN, Drug-Related Eosinophilia with Systemic Symptoms (DRESS), or Erythema Multiforme (EM) should always be categorized as Grade 3.

5.3.6.3 Rash Treatment

Antihistamines and topical corticosteroids may provide symptomatic relief to subjects who develop rash and experience associated symptoms such as pruritis. Permitted topical and systemic antihistamine drugs allowed for use for all grades of rash include diphenhydramine (Benadryl), hydroxyzine, levocetirizine (Xyzal), and desloratadine (Clarinex). The following antihistamines are metabolized by CYP3A4 and are prohibited with the use of TVR due to potentially severe side effects: astemizole, terfenadine and fexofenadine.

Because immunosuppression due to systemic corticosteroids can cause significant elevations in HCV RNA levels, systemic corticosteroids should be used only when clinically necessary and after sufficient efforts using other treatments and measure have been employed, when possible, including consultation with a dermatologist. Co-administration of systemic corticosteroids and telaprevir is not recommended due to potentially significant drug interactions. See local label guidelines.

Topical corticosteroid use is permitted, but should be limited to brief periods, eg, up to 2 weeks of continuous/regular use, and limited to use on up to 50% of the body surface. Use of topical corticosteroids for longer than 2 weeks or over more than 50% of the body surface should be discussed with the central Medical Monitor. If topical corticosteroids are needed, cream or lotion preparations are recommended, due to a lower absorptions potential. Use of gel or ointment preparations of topical corticosteroids is discouraged due to a relative higher absorption potential. Treatment of skin eruptions with investigational agents or use of approved medications in an off-label manner is also discouraged.

5.3.6.4 Rash Management for TVR

Management of Grade 1 or 2 Rash

General recommendations: For subjects experiencing a Grade 1 or 2 rash, medical management will be at the discretion of the investigator and should follow generally accepted medical standards. For subjects with a Grade 2 rash, a consultation with a specialist in dermatology should be considered. Medications to help alleviate symptoms are described above and may be employed. In addition, subjects experiencing skin rash or pruritis should be advised of other strategies to minimize the intensity or progression of their signs and symptoms (eg, limiting sun exposure and heat; baking soda or oatmeal baths; loose-fitting clothing). Rash associated with RBV may complicate the assessment of rash in subjects receiving TVR; in all cases, the investigator should follow subjects carefully if there are any skin adverse events.

Approved v4.0 930056810 4.0

Study drug discontinuation: For subjects experiencing a Grade 1 rash, discontinuation of Lambda/alfa-2a, RBV and/or TVR is generally not necessary. However, for subjects experiencing a progressive Grade 2 rash, discontinuation of study drug dosing should be considered. If discontinuation of study drug due to rash is necessary, it is recommended that TVR be discontinued first. If the rash does not improve, symptomatically or objectively, within 7 days following TVR discontinuation, RBV use should be interrupted. Interruption of RBV dosing may be done sooner if the rash worsens despite discontinuation of TVR. Lambda/alfa-2a may be continued unless interruption is also medically indicated.

Resumption of study drug dosing: TVR dosing cannot be restarted after it is discontinued for rash. Lambda/alfa-2a and/or RBV may be restarted if interrupted due to rash if there is subsequent improvement in the rash within 14 days following their respective discontinuation. Resumed administration of either Lambda/alfa-2a and/or RBV is not permitted following 14 days after their interruption for rash, respectively. RBV monotherapy is not permitted so if Lambda/alfa-2a and/or RBV are interrupted and RBV is restarted, Lambda/alfa-2a must also be restarted.

Management: Subjects should be closely monitored for any progression or worsening of signs or symptoms of systemic involvement. Consider consultation with a specialist in dermatology for any subject with a Grade 2 rash. For subjects with a Grade 1 or 2 rash that results in permanent discontinuation of any study drug the event meets criteria for EOSI. For subjects with a rash that does not result in changes to study drug dosing, additional diagnostic work-up will be at the investigator's discretion, as clinically indicated.

In all cases, subjects should be followed until the rash has resolved completely.

Management of Grade 3 Rash

General recommendations: For subjects experiencing a Grade 3 rash, the same general recommendations for subjects experiencing Grade 1 and Grade 2 rashes apply. Consultation with a specialist in dermatology is recommended. Refer to Section 5.3.7 for management of serious skin reactions.

Study drug discontinuation: For subjects experiencing a Grade 3 rash, TVR should be discontinued immediately. If the rash does not improve, symptomatically or objectively within 7 days following TVR discontinuation, RBV use should also be interrupted.

Interruption of RBV dosing may be done sooner if the subject's rash worsens despite discontinuation of TVR. Lambda/alfa-2a may be continued unless interruption is also medically indicated. The investigator may discontinue all study drugs simultaneously and immediately, if clinically indicated.

However, any subjects diagnosed with or suspected to have SJS, TEN, DRESS, EM, or a skin rash considered life threatening must have all study drugs permanently discontinued immediately.

Management: Close clinical follow-up and appropriate medical intervention should be instituted. Daily follow-up in person or by telephone to monitor for progression of the event may be necessary from the onset of the event until improvement is observed. Additional visits should be

Approved v4.0 930056810 4.0

performed (eg, each week or more often as clinically appropriate). All subjects should be followed until rash has resolved completely.

A consultation with a dermatologist for further characterization of the rash and skin biopsy is highly recommended, and additional testing should be performed as needed, such as WBC with differential, ALT/AST, serum creatinine, CPK (creatinine phosphokinase, and LDH (lactate dehydrogenase) etc.

5.3.7 Serious Skin Reactions

For serious skin reactions, including rash with systemic symptoms or a progressive severe rash, TVR, pegIFN and RBV must be discontinued immediately. Discontinuing other medications known to be associated with serious skin reactions should be considered. Subjects should be promptly referred to urgent medical care including consultation with a dermatologist.

5.4 Efficacy Assessments

Only data for the procedures and assessments specified in this protocol should be submitted to BMS on a case report form. Additional procedures and assessments may be performed as part of the subject's standard medical care; however data for these assessments should remain in the subject's medical record and should not be provided to BMS, unless specifically requested by the sponsor.

5.4.1 Primary Efficacy Assessment

HCV RNA will be measured at Weeks 4 and 12 (Part A), and post-treatment follow-up Week 12 (Part B) for the primary antiviral assessment in this study.

5.4.2 Secondary Efficacy Assessments

The HCV RNA measured at Weeks 4, 12, and follow-up Week 24 will be used for the secondary antiviral assessments in this study. HCV RNA measured at other time points are used for other antiviral assessments in this study.

5.5 Pharmacokinetic Assessments

Table 5.5A and Table 5.5B lists the sampling schedule for the assessment of pharmacokinetics of Lambda (or alfa-2a) and TVR. Further details of blood collection and processing will be provided to the site in the procedure manual. The number of subjects for the intensive PK studies will be up to 20 subjects from either Part A or Part B (minimum 16 subjects of which 5 subjects are from Part A). The initiation of Part B will not be dependent on the availability of PK data from part A. However, it will be dependent on availability of safety findings from part A.

Approved v4.0 930056810 4.0

Table 5.5A: PK Sampling Schedule (All Subjects in Parts A and B)

Time point	Time (Event) Hour	Time (Relative To Dosing) Hour:Min	Blood Sample (PK)	
			Lambda or alfa-2a (Serum)	TVR (Plasma)
Baseline (Day 1)	0 h (pre-dose)	00:00	X	X
Week 2 (Day 15)	0 h (pre-dose)	00:00	X	X
Week 4 (Day 29)	0 h (pre-dose)	00:00	X	X
Week 12 (Day 85)	0 h (pre-dose)	00:00	X	X
Week 24 (Day 169)	0 h (pre-dose)	00:00	X	
Number of Samples			5	4

Table 5.5B: PK Sampling Schedule (Intensive PK Subjects in Part A and Part B)

<table>
<tr><th colspan="2" rowspan="2">Time point</th><th rowspan="2">Time (Event)
Hour</th><th rowspan="2">Time
(Relative To Dosing)
Hour:Min</th><th colspan="2">Blood Sample
(PK)</th></tr>
<tr><th>Lambda or alfa-2a (Serum)</th><th>TVR (Plasma)</th></tr>
<tr><td colspan="2">Baseline (Day 1)</td><td>0 h (pre-dose)</td><td>00:00</td><td>X</td><td>X</td></tr>
<tr><td colspan="2">Week 2 (Day 15)</td><td>0 h (pre-dose)</td><td>00:00</td><td>X</td><td>X</td></tr>
<tr><td rowspan="14">Week 4 (Days 29-35)</td><td rowspan="8">Day 29</td><td>0 h (pre-dose)</td><td>00:00</td><td>X</td><td>X</td></tr>
<tr><td>0.5h</td><td>00:30</td><td>X</td><td>X</td></tr>
<tr><td>1 h</td><td>01:00</td><td>X</td><td>X</td></tr>
<tr><td>2.5 h</td><td>02:30</td><td>X</td><td>X</td></tr>
<tr><td>4 h</td><td>04:00</td><td>X</td><td>X</td></tr>
<tr><td>6 h</td><td>06:00</td><td>X</td><td>X</td></tr>
<tr><td>8 h</td><td>08:00</td><td>X</td><td>X</td></tr>
<tr><td>12h</td><td>12:00</td><td>X</td><td></td></tr>
<tr><td>Day 30</td><td>0 h</td><td>24:00</td><td>X</td><td></td></tr>
<tr><td>Day 31</td><td>0 h</td><td>48:00</td><td>X</td><td></td></tr>
<tr><td>Day 32</td><td>0 h</td><td>72:00</td><td>X</td><td></td></tr>
<tr><td>Day 33</td><td>0 h</td><td>96:00</td><td>X</td><td></td></tr>
<tr><td>Day 34</td><td>0 h</td><td>120:00</td><td>X</td><td></td></tr>
<tr><td>Day 35</td><td>0 h</td><td>144:00</td><td>X</td><td></td></tr>
</table>

Approved v4.0 930056810 4.0

Table 5.5B: PK Sampling Schedule (Intensive PK Subjects in Part A and Part B)

Time point	Time (Event) Hour	Time (Relative To Dosing) Hour:Min	Blood Sample (PK)	
			Lambda or alfa-2a (Serum)	TVR (Plasma)
Week 5 (Day 36)	0 h (pre-dose)	00:00	X	
Week 12 (Day 85)	0 h (pre-dose)	00:00	X	X
Week 24 (Day 169)	0 h (pre-dose)	00:00	X	
Number of Samples			19	10

The serum samples will be analyzed for Lambda by a validated MSD-ECL assay. In addition, the plasma samples for TVR will be archived for potential analysis, if the need arises.

5.6 Biomarker Assessments

5.6.1 Pharmacodynamic Assessments

Markers of host immune response will be monitored to determine the relationship between host immune response and clinical responses to Lambda/RBV/TVR or alfa-2a/RBV/TVR. Improvement in immune response markers has been described for alfa-2a/RBV therapy. This study will compare the changes in host immune responses obtained with alfa-2a/RBV/TVR therapy to Lambda/RBV/TVR therapy and determine if there is an association with improved host immune response and clinical response.

Serum protein markers may include but are not limited to: IP-10, iTAC, Mig, MCP-1, IL6, and IFN gamma. Whole blood will be collected at baseline and during treatment to potentially explore changes in host gene expression. Genes to be measured will be related to anti-viral or immune responses and may include but are not limited to: Interferon stimulated genes (MX1, IFIT1, ISG15, OAS1, OAS2, OAS3, OASL), signaling genes (STAT1, STAT2, SOCS1, SOCS3) and IFN receptors (IFNAR1, IFNAR2, IL28RA, IL10RB).

5.6.2 Pharmacogenomic/Pharmacogenetic Assessments

The rs12979860 SNP in the IL28B gene has been associated with improved rates of SVR with alfa-2a/RBV therapy. The relationship of this SNP GT with virologic endpoints will be analyzed. Additional exploratory analysis may be performed to investigate the role of other SNPs in IL28B and ENT1 (a RBV transporter gene) with virologic responses in this study. In addition, SNPs in ITPA may be examined to be correlated to anemia if deemed necessary. A blood sample for potential bridging of rs12979860 IL28B SNP assay will also be collected at baseline for storage (only to be analyzed if additional validation of rs12979860 IL28B SNP assay is required).

Approved v4.0 930056810 4.0

5.7 Outcomes Research Assessments

Patient reported outcomes are important parameters to gauge subjects' experience with treatment and can be a key predictor of persistence with treatment. Mean scores for the Flu-Like Symptom Index (sub-set of the hepatitis physical symptom diary (HPSS-D) at week 12 (from EMERGE) for the alfa-2a group (N = 36) increased from baseline by 2.39 (sd = 4.95) as compared to 0.83 (sd = 5.05) for Lambda subjects (N = 26). The percent of subjects reporting any flu-like symptoms was significantly ($p < .001$) higher for alfa-2a (75%) as compared to Lambda subjects (27%) at Week 12 despite similar rates at study start (alfa-2a: 47%, Lambda: 42%). Similarly, mean total fatigue scores (as measured by the fatigue severity scale – FSS) at baseline for subjects in EMERGE were worse than those for a healthy adult population. Subjects on alfa-2a and Lambda reported increasing fatigue when on treatment. The increase in fatigue was significantly worse with alfa-2a than Lambda.

5.7.1 Patient Health Questionnaire (PHQ-9)

The PHQ-9 is a screening instrument implemented in medical setting to evaluate symptoms of depression. It is a self-report inventory developed to assess the existence and severity of symptoms of depression from a patient perspective and will be use to assess real-time on treatment emerging depressive symptoms during treatment visits. The PHQ-9 consists of 9 items which can be scored from 0 (not at all) to 3 (nearly every day). The PHQ-9 score can range from 0 to 27.

Subjects with treatment-emerging depression (PHQ-9 ≥ 15) will be further evaluated for suicidality with the Columbia Suicide Severity Rating Scale (C-SSRS). All identified suicidal cases will be referred to a mental health professional for management.

5.7.2 Columbia-Suicide Severity Rating Scale (C-SSRS)

For subjects with a PHQ-9 ≥ 15 or who express suicidal ideation the C-SSRS will be used. The C-SSRS is a scale that assesses the intensity and presence of suicidal ideation and behavior. It maps to the Columbia-Classification Algorithm for Suicide Assessment (C-CASA) and meets the criteria listed in the recent FDA draft guidance (The US Department of Health and Human Services Food and Drug Administration Center for Drug Evaluation and Research (CDER): DRAFT Guidance for Industry Suicidality: Prospective Assessment of Occurrence in Clinical Trials, September 2010).

5.7.3 'Flu-like symptom' index, as an exploratory endpoint, using the 'HPSS-D' diary

This assessment will be based on a subset of questions of the Hepatitis Physical Symptom Severity diary (HPSS-D) and will consist of the following symptoms fever, chills, muscle ache and joint pain. This endpoint will be defined as proportion of subjects reporting 'flu-like symptoms' which is defined as the presence of any of these symptoms as reported by subjects. In addition severity of these symptoms will be defined as the total score across these four items.

Approved v4.0 930056810 4.0

The endpoint will be defined as the change in the proportion of subjects reporting 'flu like symptoms' from baseline to Week 12 and as the change in the mean score from baseline to Week 12.

The HPSS-D will be operationalized as a diary that subjects will complete for seven consecutive days at certain pre-defined intervals during the course of the study. The diary will require subjects to assess the severity of each symptom based on the previous 24 hour period.

5.7.4 Fatigue Severity Scale

The FSS is a self-administered instrument designed to assess the effect of fatigue on everyday life. Subjects are required to indicate the level of agreement with a series of 9 items as recalled over the previous 2 weeks. Item responses are measured using a Likert type scale ranging from 1 (strongly disagree) to 7 (strongly agree). The 9 items are combined into a total fatigue score, where higher scores indicate greater impact of fatigue on everyday life. The FSS total score is calculated as the average of the individual item responses.

5.7.5 EQ-5D (to measure patient utilities for Health Authority submissions)

The EQ-5D is the most widely used generic preference-based measure of health-related quality of life which produces utility scores anchored at 0 for dead and 1 for perfect health. The utility scores represent preferences for particular health states. The descriptive system has 5 dimensions (mobility, self-care, usual activity, pain/discomfort and anxiety/depression) and 5 levels (no problems, slight problems, moderate problems, severe problems, and extreme problems). The EQ-5D valued using the UK TTO value set is preferred by NICE.

5.8 Other Assessments

5.8.1 Resistance Monitoring

To characterize the pre-existence, emergence and persistence of HCV viral variants associated with exposure to telaprevir, plasma samples from all subjects will be obtained on Days 1 (baseline) and at Weeks 1, 2, 4, 8, 12 during treatment with TVR, and at Weeks 16, 24, and 48 while treatment continues with pegylated interferon lambda and ribavirin. Blood samples for HCV resistance genotyping will also be obtained at Weeks 4, 12, 24, and (36 and 48 for subjects who fail treatment) post-treatment.

For subjects who fail telaprevir-based treatment, baseline samples will be analyzed for the pre-existence of HCV NS3 protease variants using population sequencing. Resistance testing will also be performed on samples close to the time of virologic failure where HCV RNA ≥ 1000 IU/mL.

Virologic failure in this protocol is defined as previously described in Section 3.1.3 and includes:

1. Any HCV RNA > 1000 IU/mL at Week 4 or 12
2. Any confirmed HCV RNA detectable from Week 24
3. Virologic breakthrough:

Approved v4.0 930056810 4.0

a. Confirmed* > 1 log_{10} increase in HCV RNA or nadir OR

b. Confirmed* HCV RNA ≥ LLOQ after previously having an HCV RNA level of < LLOQ (target detected or target not detected) while on treatment.

*Measurements should ideally be confirmed within 2 weeks of the original result

Analysis of additional time points representing either virologic breakthrough with HCV RNA < 1000 IU/mL or persistence of emerging TVR-resistant variants will be performed at the discretion of the virology, statistical and medical teams. Study data relevant to the performance and interpretation of the resistance testing will be shared between the medical, statistical and virology teams under the direction of the medical monitor.

A single member (or designate) of the BMS discovery virology group will be notified of subjects who meet criteria for virologic failure and are randomized to active treatment (telaprevir + Lambda + RBV) study arms. The purpose is to minimize the unnecessary resistance analysis of samples from subjects receiving TVR + pegIFN α -2a/RBV.

The pre-existence of resistance-associated variants at baseline, the emergence of drug-resistant variants or any sequence changes in the NS3 protease region will be monitored and compared with the respective baseline sample sequences and reference sequences 1a(H77) and 1b(Con1). For subjects who have detectable, well characterized resistance substitutions at the EOT that are known to persist, the Week 48 post-treatment time-point will be evaluated. For less characterized or novel resistance substitutions, resistance testing will be carried out every 12 weeks post-treatment to determine the pattern of decay up to Week 48 post-treatment. If these less characterized or novel substitutions have reverted to baseline sequence, genotypic analysis of the Week 4 post-treatment time-point will be evaluated. For subjects who have undetectable viral RNA at the EOT, genotypic analysis of sample time-points post-treatment will be evaluated if viral relapse is detected.

Clonal analysis may be employed to quantify novel resistance substitutions that appear as a mixture or to determine any linkage with other variants with respect to time-on-treatment to assess the rate of emergence and persistence of resistance variants. Clonal analysis of the respective baseline sequences will also be compared. If the virologic failure of a particular subject sample cannot be explained genotypically, the subject sample quasi species encoding the respective target regions will be phenotyped.

For a novel emerging NS3 protease variant that may (or may not) be linked to a previously reported telaprevir-resistant variant whose phenotype does not explain virologic failure, a replicon construct containing specific substitutions or the NS3 protease region represented by the subject population will be analyzed. The resistance profile and replication capacity (fitness) will be determined in the presence of telaprevir in a transient HCV replicon reporter assay. If the resistant phenotype (in combination with knowledge of drug exposure and compliance) does not explain treatment failure, genotypic analysis will be expanded to include the subject NS4A and NS3 protease cleavage sites.

Approved v4.0 930056810 4.0

5.8.2 Immunogenicity Assessment

Anti-Lambda antibodies will be assessed at protocol defined time points: baseline, dosing phase Weeks 2, 4, 12, 24 and post-treatment follow-up Weeks 12, 24, 36, and 48 (Table 5.1B and Table 5.1C). Analysis of anti-Lambda antibodies will be restricted to subjects who are treated with Lambda. Analysis of anti-alfa-2a antibodies will be restricted to subjects who are treated with Pegasys.

The immunogenicity testing strategy is a tiered approach that is designed to screen for binding antibodies, confirm antibody specificity, determine antibody titer, and assesses the neutralizing potential of antibodies to Lambda (or alfa-2a) subject serum samples. The binding antibody method is a fully validated electrochemiluminescent-based assay that utilizes drug product in a bridging format (biotin-Lambda capture and ruthenium-Lambda detection) to detect all antibody isotypes that may be present in clinical samples. The neutralizing antibody assay is a fully validated cell-based bioassay that assesses the neutralizing potential of anti-Lambda antibodies that may be present in clinical samples.

The first tier in the binding antibody assay screens subject samples for the presence of anti-Lambda antibodies. Samples that have values greater than or equal to the screening cut-point, established during validation to result in a 5% false positive rate, are considered reactive, and are assayed in the second tier. The second tier confirms antibody specificity to Lambda drug product through immunodepletion whereby excess soluble Lambda drug product is used to compete with the assay bridging components (biotin-Lambda and ruthenium-Lambda) for binding to any anti-Lambda antibodies that might be present in the subject sample. Samples that have % inhibition values greater than or equal to the confirmatory cut-point established during validation, are considered to be positive for Lambda antibodies. In Tier 3, antibody titer is determined through serial dilution of confirmed positive samples. The relative sensitivity of the binding antibody assay is ≤ 100 ng/mL using an affinity purified cynomolgus monkey polyclonal antibody control. In Tier 4, antibody neutralization potential is determined using a cell-based bioassay. Samples that have % neutralization values greater than or equal to the neutralization cut-point established during validation, are determined to be positive for neutralization potential. The relative sensitivity of the neutralization antibody assay, based on an affinity purified cynomolgus monkey polyclonal antibody control, is ≤ 1 μg/mL.

6 ADVERSE EVENTS

An ***Adverse Event (AE)*** is defined as any new untoward medical occurrence or worsening of a preexisting medical condition in a clinical investigation subject administered an investigational (medicinal) product and that does not necessarily have a causal relationship with this treatment. An AE can therefore be any unfavorable and unintended sign (such as an abnormal laboratory finding), symptom, or disease temporally associated with the use of investigational product, whether or not considered related to the investigational product.

The causal relationship to study drug is determined by a physician and should be used to assess all AEs. The casual relationship can be one of the following:

Approved v4.0 930056810 4.0

Related: There is a reasonable causal relationship between study drug administration and the AE.

Not related: There is not a reasonable causal relationship between study drug administration and the AE.

The term "reasonable causal relationship" means there is evidence to suggest a causal relationship.

Adverse events can be spontaneously reported or elicited during open-ended questioning, examination, or evaluation of a subject. (In order to prevent reporting bias, subjects should not be questioned regarding the specific occurrence of one or more AEs.)

6.1 Serious Adverse Events

A S***erious Adverse Event (SAE)*** is any untoward medical occurrence that at any dose:

- results in death
- is life-threatening (defined as an event in which the subject was at risk of death at the time of the event; it does not refer to an event which hypothetically might have caused death if it were more severe)
- requires inpatient hospitalization or causes prolongation of existing hospitalization (see **NOTE** below)
- results in persistent or significant disability/incapacity
- is a congenital anomaly/birth defect
- is an important medical event (defined as a medical event(s) that may not be immediately life-threatening or result in death or hospitalization but, based upon appropriate medical and scientific judgment, may jeopardize the subject or may require intervention [eg, medical, surgical] to prevent one of the other serious outcomes listed in the definition above.) Examples of such events include, but are not limited to, intensive treatment in an emergency room or at home for allergic bronchospasm; blood dyscrasias or convulsions that do not result in hospitalization.) Clinical jaundice is considered to be an important medical event for this study and should be reported as an SAE. Potential drug induced liver injury (DILI) is also considered an important medical event. (See Section 6.6 for the definition of potential DILI.)

Suspected transmission of an infectious agent (eg, pathogenic or nonpathogenic) via the study drug is an SAE.

Although pregnancy, overdose, cancer, and potential drug induced liver injury (DILI) are not always serious by regulatory definition, these events must be handled as SAEs. (See Section 6.1.1 for reporting pregnancies).

NOTE:

The following hospitalizations are not considered SAEs in BMS clinical studies:

– a visit to the emergency room or other hospital department < 24 hours, that does not result in admission (unless considered an important medical or life-threatening event)

Approved v4.0 930056810 4.0

- elective surgery, planned prior to signing consent
- admissions as per protocol for a planned medical/surgical procedure
- routine health assessment requiring admission for baseline/trending of health status (eg, routine colonoscopy)
- medical/surgical admission other than to remedy ill health and planned prior to entry into the study. Appropriate documentation is required in these cases
- admission encountered for another life circumstance that carries no bearing on health status and requires no medical/surgical intervention (eg, lack of housing, economic inadequacy, caregiver respite, family circumstances, administrative reason).

6.1.1 Serious Adverse Event Collection and Reporting

Following the subject's written consent to participate in the study, all SAEs, whether related or not related to study drug, must be collected, including those thought to be associated with protocol-specified procedures. All SAEs must be collected that occur during the screening period and within 30 days of discontinuation of dosing. The investigator should report any SAE that occurs after these time periods and that is believed to be related to study drug or protocol-specified procedure.

An SAE report should be completed for any event where doubt exists regarding its seriousness.

If the investigator believes that an SAE is not related to study drug, but is potentially related to the conditions of the study (such as withdrawal of previous therapy or a complication of a study procedure), the relationship should be specified in the narrative section of the SAE Report Form.

SAEs, whether related or not related to study drug, and pregnancies must be reported to BMS (or designee) within 24 hours. SAEs must be recorded on the SAE Report Form (electronic); pregnancies on a Pregnancy Surveillance Form (electronic or paper forms). When using paper forms, the reports are to be transmitted via email or confirmed facsimile (fax) transmission to:

SAE Email Address: See Contact Information list.
SAE Facsimile Number: See Contact Information list.

For studies capturing SAEs/pregnancies through electronic data capture (EDC), electronic submission is the required method for reporting. The paper forms should be used and submitted immediately, only in the event the electronic system is unavailable for transmission. When paper forms are used, the original paper forms are to remain on site.

SAE Telephone Contact (required for SAE and pregnancy reporting): See Contact Information list.

If only limited information is initially available, follow-up reports are required. (Note: Follow-up SAE reports should include the same investigator term(s) initially reported.)

If an ongoing SAE changes in its intensity or relationship to study drug or if new information becomes available, a follow-up SAE report should be sent within 24 hours to the BMS (or designee) using the same procedure used for transmitting the initial SAE report.

Approved v4.0 930056810 4.0

All SAEs should be followed to resolution or stabilization.

6.2 Nonserious Adverse Events

A ***nonserious adverse event*** is an AE not classified as serious.

6.2.1 Nonserious Adverse Event Collection and Reporting

The collection of nonserious AE information should begin at initiation of study drug. Nonserious AE information should also be collected from the start of a placebo lead-in period or other observational period intended to establish a baseline status for the subjects.

Nonserious AEs should be followed to resolution or stabilization, or reported as SAEs if they become serious (see Section 6.1.1). Follow-up is also required for nonserious AEs that cause interruption or discontinuation of study drug and for those present at the end of study treatment as appropriate. Nonserious AEs must be collected during the on-treatment period and through post-treatment follow-up Week 24. All identified nonserious AEs must be recorded and described on the nonserious AE page of the CRF (paper or electronic).

Completion of supplemental CRFs may be requested for AEs and/or laboratory abnormalities that are reported/identified during the course of the study.

6.3 Laboratory Test Result Abnormalities

The following laboratory test result abnormalities should be captured on the nonserious AE CRF page or SAE Report Form (paper or electronic) as appropriate:

- Any laboratory test result that is clinically significant or meets the definition of an SAE
- Any laboratory test result abnormality that required the subject to have study drug discontinued or interrupted
- Any laboratory test result abnormality that required the subject to receive specific corrective therapy.

It is expected that wherever possible, the clinical rather than laboratory term would be used by the reporting investigator (eg, anemia versus low hemoglobin value).

If grading of laboratory abnormalities is reported as AE or SAE, the Division of AIDS table for Grading the Severity of Adult and Pediatric Adverse Events should be used (Appendix 2).

6.4 Pregnancy

If, following initiation of the investigational product, it is discovered that a study subject is pregnant or may have been pregnant at the time of investigational product exposure, including during at least 6 half lives after product administration, the investigational product will be permanently discontinued in an appropriate manner (eg, dose tapering if necessary for subject safety). Protocol-required procedures for study discontinuation and follow-up must be performed on the subject unless contraindicated by pregnancy (eg, x-ray studies). Other appropriate pregnancy follow-up procedures should be considered if indicated.

Approved v4.0 930056810 4.0

The investigator must immediately notify the BMS (or designee) Medical Monitor of this event and complete and forward a Pregnancy Surveillance Form to BMS (or designee) within 24 hours and in accordance with SAE reporting procedures described in Section 6.1.1.

Follow-up information regarding the course of the pregnancy, including perinatal and neonatal outcome and, where applicable, offspring information must be reported on the Pregnancy Surveillance Form.

Any pregnancy that occurs in a female partner of a male study participant should be reported to the sponsor. Information on this pregnancy will be collected on the Pregnancy Surveillance Form.

6.5 Overdose

An overdose is defined as the accidental or intentional administration of any dose of a product that is considered both excessive and medically important. All occurrences of overdose must be reported as an SAE. All occurrences of overdose must be reported as SAEs (see Section 6.1.1 for reporting details).

6.6 Potential Drug Induced Liver Injury (DILI)

Wherever possible, timely confirmation of initial liver-related laboratory abnormalities should occur prior to the reporting of a potential DILI event. All occurrences of potential DILIs, meeting the defined criteria, must be reported as SAEs (see Section 6.1.1. for reporting details).

Potential drug induced liver injury is defined as:

1. ALT ≥ 5 times baseline or nadir value, whichever is lower

   AND

2. ALT ≥ 10 times upper limit of normal (ULN)

   AND

3. Total bilirubin ≥ 2 times ULN,

   AND

4. No other immediately apparent possible causes of ALT elevation and hyperbilirubinemia, including, but not limited to, acute viral hepatitis, cholestasis, pre-existing hepatic disease excluding HCV or the administration of other drug(s), herbal medications and substances known to be hepatotoxic.

Subjects who meet the DILI criteria should discontinue study treatment (all drugs). If a subject meets the drug discontinuation criteria, please contact the study Medical Monitor to discuss the case, prior to discontinuation.

Approved v4.0 930056810 4.0

For subjects who meet the criteria for discontinuation based on liver abnormalities, it is strongly recommended that the following evaluations be performed (See Section 4.3.1.1 - management of subjects with impaired liver function):

- Imaging studies for a possible extrahepatic cholestasis (ie, ultrasound)
- When etiology remains unclear, liver biopsy (if clinically feasible) with light and electron microscopic assessment

6.7 Other Safety Considerations

Any significant worsening noted during interim or final physical examinations, electrocardiogram, x-ray filming, any other potential safety assessment required or not required by protocol should also be recorded as a nonserious or serious AE, as appropriate, and reported accordingly.

7 DATA MONITORING COMMITTEE AND OTHER EXTERNAL COMMITTEES

In Part B of this study, an independent DMC will periodically review interim safety data. The DMC will review data by masked treatment group, but can request unblinded data by treatment group or by individual subject as they deem necessary. The schedule of review will be defined in the DMC charter. Data to be reviewed will include, but are not limited to, adverse events and laboratory values. In addition, all expedited serious adverse event reports will be provided to the DMC members concurrent with the submission to the regulatory authorities. Antiviral data will also be provided to the DMC to facilitate assessments of benefit vs. risk. After each review, the DMC will provide the Medical Review Team (MST) with minutes blinded to treatment assignment and any recommendations. After consideration, the MST will inform the DMC of any action that will be taken in response to the DMC's recommendations.

The role and responsibilities of the DMC, its operational procedures, and methods of communication with the Sponsor will be described in a separate DMC charter. The DMC will consist of a minimum of 3 expert members who are independent of the Sponsor. The members will be appointed by the Sponsor based on their expertise in biostatistics, hepatology, and internal medicine. All DMC members will have experience in the conduct of clinical trials. Members will not be investigators in the trial, nor will they have any conflict of interest with the Sponsor. Sponsor representatives are not eligible for membership on the DMC.

Approved v4.0 930056810 4.0

8 STATISTICAL CONSIDERATIONS

8.1 Sample Size Determination

(1) Part A

The target sample size of 25 subjects in the Lambda/RBV/TVR treatment regimen can detect, with more than 72% probability, a safety event that occurs at an incident rate of 5%. The probability will be > 92% for a safety event that occurs at an incident rate of 10%.

With 25 subjects in Part A, if the discontinuation rate due to drug related events is 10%, there will be a > 99% chance to proceed to Part B based on the go/no go decision. If the discontinuation rate is 20%, the chance to proceed to Part B is 78%.

(2) Part B

A two-stage evaluation of the efficacy of Lambda compared to alfa-2a is planned. In the first stage, the non-inferiority of Lambda to alfa-2a will be tested. Sample size calculations for the non-inferiority testing assume same response rate for SVR12 in both treatment arms. Provided non-inferiority is established, a second stage test will be conducted to demonstrate superiority if there are larger improvements of clinical importance. Because the test for superiority will be conducted only if the test for non-inferiority is successful, significance levels will not be adjusted for the first stage of testing.

Non-inferiority

With 406 Lambda treated subjects and 203 alfa-2a treated subjects, there is 95% power to demonstrate non-inferiority of Lambda to alfa-2a, for the proportion of subjects with SVR12 at post-dosing follow up Week 12, assuming:

- A response rate of 79% for both Lambda and alfa-2a
- A -12% boundary for comparison with the lower limit of the two-sided 95% confidence interval for the treatment difference (λ - α)

Superiority

With 406 Lambda treated subjects and 203 alfa-2a treated subjects, there is > 90% power for testing superiority of Lambda compared to alfa-2a assuming:

- A 79% response rate for alfa-2a and an 89% rate for Lambda
- A type I error of 0.05 (two-sided)

Subgroup analysis on the naïves

Assuming 80% of the subjects are naive, there will be 324 Lambda treated and 162 alfa-2a treated naive subjects. This sample size will provide 90% power for the non-inferiority comparison and 82% power for the superiority comparison between two treatment groups in the naive subjects, based on the same assumptions as above.

Approved v4.0 930056810 4.0

The primary efficacy endpoint will be analyzed using modified intent to treat (ITT):

Modified ITT: The numerator is based on subjects meeting the response criteria. The denominator is based on all treated subjects.

8.2 Populations for Analyses

- Enrolled subjects are those who signed an informed consent form and were assigned a Patient Identification number (PID).
- Randomized subjects are enrolled subjects who received a treatment assignment from the IVRS.
- Treated subjects are randomized subjects who received at least 1 dose of study therapy.

8.3 Endpoints

8.3.1 Primary Endpoint(s)

Part A:

Proportion of subjects treated with Lambda/RBV/TVR who achieve eRVR, defined as HCV RNA < LLOQ target not detected at Weeks 4 and 12 of treatment, and the proportion of subjects treated with Lambda/RBV/TVR who develop safety related events (as measured by the frequency of deaths, SAEs, drug related AEs, dose reductions and discontinuations due to AEs) through end of treatment (maximum of 48 weeks) of Lambda/RBV/TVR in a sentinel cohort of subjects with GT-1 chronic HCV infection.

Part B:

Proportion of subjects who achieve efficacy as measured by SVR12, defined as HCV RNA <LLOQ (target detected or target not detected) at Week 12 of post-treatment follow-up of Lambda/RBV/TVR compared to alfa-2a/RBV/TVR in subjects with GT-1 chronic HCV infection who are treatment-naive or who relapsed on prior alfa/RBV therapy.

8.3.2 Secondary Endpoint(s)

Part A:

- Proportion of subjects who achieve efficacy as measured by SVR12, defined as HCV RNA < LLOQ (target detected or target not detected) at Week 12 of post-treatment follow-up
- Proportion of subjects who achieve efficacy as measured by SVR24, defined as HCV RNA < LLOQ (target detected or target not detected) at Week 24 of post-treatment follow-up

Part B:

- Proportion of subjects who achieve efficacy as measured by SVR12, defined as HCV RNA < LLOQ (target detected or target not detected) at Week 12 of post-treatment follow-up in treatment-naïve subjects

Approved v4.0 930056810 4.0

- Cytopenic abnormalities (anemia is defined by Hb < 10 g/dL, neutropenia as defined by ANC < 750 mm3, thrombocytopenia as defined by platelets < 50,000 mm3) through end of treatment (maximum of 48 weeks)
- Proportion of subjects who achieve efficacy as measured by eRVR, defined as HCV RNA < LLOQ (target not detected) at Weeks 4 and 12 of treatment
- Flu-like symptoms (as defined by pyrexia or chills or pain) through end of treatment (maximum of 48 weeks)
- Musculoskeletal symptoms (as defined by arthralgia or myalgia or back pain) through end of treatment (maximum of 48 weeks)
- Proportion of subjects who achieve efficacy as measured by SVR 24, defined as HCV RNA < LLOQ (target detected or target not detected) at Week 24 of post-treatment follow-up

8.3.3 Other Secondary Endpoints

- Proportion of subjects who achieve efficacy as measured by virologic response at Week 48 of post-treatment follow-up
- Safety as measured by the frequency of deaths, SAEs, drug related AEs, dose reductions and discontinuations due to AEs and treatment emergent laboratory abnormalities through follow-up Week 4
- Constitutional symptoms (fatigue or asthenia) through end of treatment (maximum of 48 weeks)
- Neurologic symptoms (headache or dizziness) through end of treatment (maximum of 48 weeks)
- Psychiatric symptoms (depression or irritability or insomnia) through end of treatment (maximum of 48 weeks)
- Occurrence of rash through end of treatment (maximum 48 weeks)

8.3.4 Exploratory Endpoint(s)

- Pharmacokinetics (PK) of Lambda/TVR compared to alfa-2a/TVR
- Exposure and antiviral response relationship
- Viral resistance to Lambda/RBV/TVR
- Biomarkers of host immune response (potentially including serum protein markers, gene expression in whole blood)
- Immunogenicity of Lambda/TVR
- SNPs in IL28B (including rs12979860) or ENT1 and response relationship
- Patient reported outcomes :
  - ‘Flu-Like’ symptoms evaluated using the Hepatitis Physical Symptom Severity Diary
  - Fatigue evaluated using the Fatigue Severity Scale
  - Depression evaluated using the Patient Health Questionnaire (PHQ-9)
  - Health-related quality of life evaluated using the EQ-5D questionnaire

Approved v4.0 930056810 4.0

8.4 Analyses

Categorical variables will be summarized with counts and percents. Confidence intervals (CI) for differences in proportions will be based on the normal approximation with unpooled proportions used in the computation of the standard error of the difference. Continuous variables will be summarized with univariate statistics (e.g., mean, median, standard error).

Longitudinal summaries of efficacy and laboratory endpoints will use pre-defined visit week windows. Windows will be constructed based on the midpoint between planned study visits. On-treatment endpoints will be assessed using measurements from the start of study therapy through the last dose of study therapy plus 10 days (based on the half-life of Lambda). Follow-up endpoints will be assessed with measurements after the last dose of study therapy plus 10 days.

Part A data will be analyzed separately from Part B. Only descriptive statistics will be provided for Part A.

8.4.1 Demographics and Baseline Characteristics

Baseline demography, HCV disease characteristics, and other baseline laboratory values will be tabulated by treatment regimen, including:

- Demographics: age, race, gender, ethnicity, geographic region;
- Disease characteristics at baseline: HCV RNA level, HCV GT, IL28B SNP GT, and cirrhosis status, naïve vs. relapser;
- Physical measurements at baseline: height, weight, body mass index;
- Laboratory tests at baseline;
- Prior medications.

8.4.2 Efficacy Analyses

A two-stage evaluation of the primary endpoint SVR12 for Part B is planned. In the first stage, the non-inferiority of Lambda to alfa-2a will be tested. The treatment difference between Lambda and alfa-2a together with the 95% two-sided confidence interval (CI) will be calculated. Non-inferiority is demonstrated if the lower limit of the 95% CI is greater than -12%. Provided non-inferiority is established, a second stage test will be conducted to demonstrate superiority for Lambda compared to alfa-2a. If the lower limit of the 95% CI is greater than 0, it will support the conclusion of superior efficacy benefit of Lambda relative to alfa-2a. Because the test for superiority will be conducted only if the test for non-inferiority is successful, significance levels will not be adjusted for these testings.

Efficacy analysis uses as-randomized treatment regimen and treated subjects. Analyses of antiviral activity will be based on HCV RNA measurements closest to the planned visits and within pre-defined visit windows.

The proportions of subjects with antiviral activity endpoints will be summarized using modified intent to treat (ITT):

Approved v4.0 930056810 4.0

- Modified ITT: The numerator is based on subjects meeting the response criteria. The denominator is based on all treated subjects;

These endpoints will also be summarized in a sensitivity analysis using observed values:

- Observed values: Similar to modified ITT, the numerator is based on subjects meeting the response criteria and the denominator is based on treated subjects with HCV RNA at visit week(s) defining the endpoint.

Treatment difference in response rate and 95% CIs will be estimated using stratum-adjusted Mantel-Haenszel (MH) approach stratified by prior relapse status (naives, relapsers), genotype subtype (1a, 1b), and IL28B rs12979860 host genotype (CC, non-CC), using modified ITT.

Provided non-inferiority in SVR12 between Lambda and alfa-2a is established in Part B, key secondary endpoints will be tested hierarchically to compare Lambda with alfa-2a at a significance level of 0.05 according to the order they are described earlier. Testing on SVR12 in naives, eRVR and SVR24 will follow the same two-stage evaluation strategy for the primary endpoint SVR12. Other key secondary endpoints will be tested for superiority. Testing of key secondary endpoints will not be proceeded if Lambda fails to demonstrate non-inferiority to alfa-2a on SVR12 in treatment-naive subjects or eRVR or once the corresponding p-values for superiority testing of the other endpoint is ≥ 0.05.

8.4.3 Safety Analyses

Deaths will be listed for enrolled subjects without regard to onset.

The frequencies of the following safety events will be summarized by treatment regimen for treated subjects:

- SAEs (separated by on treatment and follow-up);
- AEs leading to discontinuation of study therapy;
- AEs (related and regardless of relationship to study therapy) by intensity;
- Treatment emergent laboratory abnormalities by toxicity grade.

The investigators will determine the intensity of AEs and the relationship of AEs to study therapy. The investigators' terms will be coded and grouped by system organ class using the latest version of the Medical Dictionary for Regulatory Activities (MedDRA) in production at BMS. AEs will be presented by system organ class and preferred term. If a subject had an AE with different intensities over time, then only the greatest intensity will be reported for a study period.

Laboratory toxicities will be graded according to the Division of AIDS (DAIDS) of the US National Institutes of Health table for grading the severity of adverse experiences (2004). The laboratory value during the study period with the highest toxicity grade will be reported for each

Approved v4.0 930056810 4.0

test. Treatment emergent laboratory abnormalities are those with highest on-treatment toxicity grade greater than the baseline toxicity grade. Levels and changes from baseline in select laboratory tests over time will be summarized by treatment regimen for treated subjects using observed values.

Cut score guidelines for the PHQ-9 are given with the recommendation that thresholds be adjusted based on the characteristics of the sample, and the purpose for use of the PHQ-9. Total score of 5-9 is considered minimal range, 10-14 is mild, 15-19 is moderate, and ≥ 20 is severe. The primary analyses will tabulate summary statistics for the actual scores and change from baseline by treatment group and visit. Percentage of subjects in the mild, moderate and severe categories will be tabulated at baseline and subsequent time points.

The proportion of subjects who had a dose reduction will be calculated by treatment regimen.

8.4.4 Pharmacokinetic Analyses

In all subjects, trough concentrations will be assessed. In a subset of up to 20 subjects from both Part A and Part B (a minimum 16 subjects, of which 5 subjects are from Part A), an intensive PK sub study will be conducted to investigate the drug-drug interaction potential following administration of Lambda with TVR at steady state.

The following steady state PK parameters of Lambda (or alfa-2a) and TVR will be derived from the serum and plasma concentrations versus time data, by non-compartmental methods by a validated pharmacokinetic analysis program. The PK parameters will be described by summary statistics:

Cmax Maximum observed concentration;
Tmax Time of maximum observed concentration;
Cmin Serum concentration 24 hours post observed dose. C0 is used as an estimate of Cmin if sample is not collected;
Ctrough Observed trough serum/plasma concentration;
AUCtau Area under the concentration-time curve, in 1 dosing interval

The PK samples collected in this study will be pooled with PK data from other studies to perform an integrated population PK analysis, the results of which will be reported separately.

8.4.5 Biomarker Analyses

For the pharmacodynamic (PD) variables to measure host immune response biomarkers (including serum protein markers and gene expression in whole blood), summary statistics will be tabulated by treatment and time for PD variables. Linear mixed effect models may be applied to the PD biomarkers with treatment, time, and treatment-by-time interaction as fixed effects if appropriate. Different covariance structures may be explored to select a suitable covariance structure for the samples from the same subject. Additional association analyses between the PD biomarkers and Lambda exposure may be explored if deemed relevant.

Approved v4.0 930056810 4.0

Genetic biomarker analyses will focus on SNPs in IL28B and ENT1. For each SNP in each candidate gene, allele and GT frequencies will be summarized by treatment regimen. Departures from Hardy Weinberg equilibrium (DHWE) will be assessed for each genetic marker. The relationship between the primary endpoint (eg, SVR) and SNPs will be explored using frequency table by treatment and GT. Logistic regression models may be used to quantify the effect of the GT if deemed appropriate. Predictors of the logistic regression may include baseline HCV RNA, SNP GT, treatment regimen and the interaction between GT and treatment.

A bioanalytical scientist in Bioanalytical Science department (or designee in the external central bioanalytical laboratory) will be unblinded to the randomization treatment assignments in order to minimize unnecessary bioanalytical analysis of samples from control group subjects.

8.4.6 Outcomes Research Analyses

HCV related flu-like index:

Similar analyses will be conducted for the HCV related physical symptoms.

'Flu-like symptom' index, as an exploratory endpoint, using the 'HPSS-D' diary:

Summary statistics for the actual scores and change from baseline will be tabulated by treatment group and visit. This endpoint will be defined as proportion of subjects reporting 'flu-like symptoms' which is defined as the presence of any of these symptoms as reported by subjects. In addition severity of these symptoms will be defined as the total score across these four items.

The endpoint will be defined as the change in the proportion of subjects reporting 'flu like symptoms' from baseline to Week 12 and as the change in the mean score from baseline to Week 12.

HCV related Physical symptoms, as an exploratory objective, using the 'HPSS-D' diary:

This endpoint will be operationalized as 'change in proportion of subjects reporting 1) Nausea 2) stomach /GI problems 3) appetite problems 4) Dizziness or Loss of balance 5) Shortness of breath 6) Skin problems 7) Sleep problems 8) Physical fatigue.

Fatigue Severity Scale:

Total FSS score will be calculated based on all 9 items, ranging from 1 (strongly disagree) to 7 (strongly agree), where higher scores indicate greater impact of fatigue on everyday life. Analyses will focus on comparing mean change from baseline to Week 12 by treatment arms.

EQ-5D (to measure patient utilities for Health Authority submissions):

The EQ-5D utilities will be summarized at baseline and Week12 by treatment arms.

8.4.7 Other Analyses

Not applicable.

Approved v4.0 930056810 4.0

8.5 Interim Analyses

Part A

The following three analyses are planned for Part A of this study:

- Week 12 analysis when all subjects in Part A complete the 12 weeks of treatment;
- SVR4 analysis among subjects in Part A who achieve eRVR when those subjects with eRVR complete the 4 weeks of post treatment follow-up;
- SVR24 analysis when all subjects in Part A complete the 24 weeks of post treatment follow-up.

Part B

The following two analyses are planned for Part B of this study:

- Primary analysis when all subjects in Part B complete the 12 weeks of post treatment follow-up;
- SVR24 analysis when all subjects in Part B complete the 24 weeks of post treatment follow-up;

A final analysis will be conducted when all subjects including both Part A and Part B complete the 48 weeks of post-treatment follow-up.

9 STUDY MANAGEMENT

9.1 Compliance

9.1.1 Compliance with the Protocol and Protocol Revisions

The study shall be conducted as described in this approved protocol. All revisions to the protocol must be discussed with, and be prepared by, BMS. The investigator should not implement any deviation or change to the protocol without prior review and documented approval/favorable opinion from the IRB/IEC of an amendment, except where necessary to eliminate an immediate hazard(s) to study subjects.

If a deviation or change to a protocol is implemented to eliminate an immediate hazard(s) prior to obtaining IRB/IEC approval/favorable opinion, as soon as possible the deviation or change will be submitted to:

- IRB/IEC for review and approval/favorable opinion
- BMS
- Regulatory Authority(ies), if required by local regulations

Approved v4.0 930056810 4.0

Documentation of approval signed by the chairperson or designee of the IRB(s)/IEC(s) must be sent to BMS.

If an amendment substantially alters the study design or increases the potential risk to the subject: (1) the consent form must be revised and submitted to the IRB(s)/IEC(s) for review and approval/favorable opinion; (2) the revised form must be used to obtain consent from subjects currently enrolled in the study if they are affected by the amendment; and (3) the new form must be used to obtain consent from new subjects prior to enrollment.

If the revision is an administrative letter, investigators must inform their IRB(s)/IEC(s).

9.1.2 Monitoring

Representatives of BMS must be allowed to visit all study site locations periodically to assess the data quality and study integrity. On site they will review study records and directly compare them with source documents, discuss the conduct of the study with the investigator, and verify that the facilities remain acceptable.

In addition, the study may be evaluated by BMS internal auditors and government inspectors who must be allowed access to CRFs, source documents, other study files, and study facilities. BMS audit reports will be kept confidential.

The investigator must notify BMS promptly of any inspections scheduled by regulatory authorities, and promptly forward copies of inspection reports to BMS.

9.1.3 Investigational Site Training

Bristol-Myers Squibb will provide quality investigational staff training prior to study initiation. Training topics will include but are not limited to: GCP, AE reporting, study details and procedure, electronic CRFs, study documentation, informed consent, and enrollment of WOCBP.

9.2 Records

9.2.1 Records Retention

The investigator must retain all study records and source documents for the maximum period required by applicable regulations and guidelines, or institution procedures, or for the period specified by BMS, whichever is longer. The investigator must contact BMS prior to destroying any records associated with the study.

BMS will notify the investigator when the study records are no longer needed.

If the investigator withdraws from the study (eg, relocation, retirement), the records shall be transferred to a mutually agreed upon designee (eg, another investigator, IRB). Notice of such transfer will be given in writing to BMS.

9.2.2 Study Drug Records

It is the responsibility of the investigator to ensure that a current disposition record of investigational product Lambda, alfa-2a, RBV and TVR is maintained at each study site where

Approved v4.0 930056810 4.0

study drug is inventoried and dispensed. Records or logs must comply with applicable regulations and guidelines and should include:

- amount received and placed in storage area
- amount currently in storage area
- label identification number or batch number
- amount dispensed to and returned by each subject, including unique subject identifiers
- amount transferred to another area/site for dispensing or storage
- nonstudy disposition (eg, lost, wasted)
- amount destroyed at study site, if applicable
- amount returned to BMS
- retain samples for bioavailability/bioequivalence, if applicable
- dates and initials of person responsible for Investigational Product dispensing/accountability, as per the Delegation of Authority Form.

BMS will provide forms to facilitate inventory control if the investigational site does not have an established system that meets these requirements.

9.2.3 Case Report Forms

An investigator is required to prepare and maintain adequate and accurate case histories designed to record all observations and other data pertinent to the investigation on each individual treated or entered as a control in the investigation. Data that are derived from source documents and reported on the CRF must be consistent with the source documents or the discrepancies must be explained.

For sites using the BMS electronic data capture tool, electronic CRFs will be prepared for all data collection fields except for fields specific to SAEs and pregnancy, which will be reported on the paper or electronic SAE form and Pregnancy Surveillance form, respectively. Spaces may be left blank only in those circumstances permitted by study-specific CRF completion guidelines provided by BMS.

The confidentiality of records that could identify subjects must be protected, respecting the privacy and confidentiality rules in accordance with the applicable regulatory requirement(s).

The investigator will maintain a signature sheet to document signatures and initials of all persons authorized to make entries and/or corrections on CRFs.

The completed CRF, including any paper or electronic SAE/pregnancy CRFs, must be promptly reviewed, signed, and dated by the investigator or qualified physician who is a subinvestigator and who is delegated this task on the Delegation of Authority Form. For electronic CRFs, review and approval/signature is completed electronically through the BMS electronic data capture tool. The investigator must retain a copy of the CRFs including records of the changes and corrections.

Approved v4.0 930056810 4.0

Each individual electronically signing electronic CRFs must meet BMS training requirements and must only access the BMS electronic data capture tool using the unique user account provided by BMS. User accounts are not to be shared or reassigned to other individuals.

9.3 Clinical Study Report and Publications

A Signatory Investigator must be selected to sign the clinical study report.

For this protocol, the Signatory Investigator will be selected considering the following criteria:

- Subject recruitment (eg, among the top quartile of enrollers)
- Regional representation (eg, among top quartile of enrollers from a specified region or country)
- Other criteria (as determined by the study team)

The data collected during this study are confidential and proprietary to BMS. Any publications or abstracts arising from this study require approval by BMS prior to publication or presentation and must adhere to BMS's publication requirements as set forth in the approved clinical trial agreement (CTA). All draft publications, including abstracts or detailed summaries of any proposed presentations, must be submitted to BMS at the earliest practicable time for review, but at any event not less than 30 days before submission or presentation unless otherwise set forth in the CTA. BMS shall have the right to delete any confidential or proprietary information contained in any proposed presentation or abstract and may delay publication for up to 60 days for purposes of filing a patent application.

Approved v4.0 930056810 4.0

10 GLOSSARY OF TERMS

Term	Definition
Adverse Reaction	An adverse event that is considered by either the investigator or BMS as related to the investigational product
Unexpected Adverse Reaction	An adverse reaction, the nature or severity of which is not consistent with the applicable product information (eg, Investigator Brochure for an unapproved investigational product)

Approved v4.0 930056810 4.0

11 LIST OF ABBREVIATIONS

Term	Definition
AE	adverse event
Alfa-2a	Peginterferon alfa-2a (PEGASYS®)
ALT	alanine amino transferase
ALP	alkaline phosphatase
ANA	Antinuclear antibody
ANC	absolute neutrophil count
AST	aspartate Aminotransferase
BID	twice daily
BMS	Bristol-Myers Squibb
BOC	Boceprevir (VICTRELIS™)
BUN	blood urea nitrogen
CC	cc host GT
cEVR	complete early virologic response
CI	confidence interval
CO_2	Bicarbonate
CPK	creatinine phosphokinase
CRF	Case Report Form
C-CASA	Columbia-Classification Algorithm for Suicide Assessment
C-SSRS	Columbia-Suicide Severity Rating Scale
CT	computed tomography
CTA	Clinical Trial Application
Ctrough	observed trough serum/plasma concentration
DAA	direct antiviral agent
DAIDS	Division of AIDS Table for Grading the Severity of Adult and Pediatric Adverse Events
DHWE	Departures from Hardy Weinberg equilibrium
DILI	drug-induced liver injury
Dl	Deciliter
DLT	dose-limiting toxicity

Approved v4.0 930056810 4.0

Term	Definition
DNA	deoxyribonucleic acid
EASL	European Association for the Study of the Liver
EC_{50}	half maximal effective concentration
ECG	Electrocardiogram
EE	ethinyl estradiol
EOSI	events of special interest
EOT	end of treatment
EMCV	encephalomyocarditis virus
ESA	erythropoiesis-stimulating agent
EVR	extended virologic response
eRVR	extended rapid virologic response
EQ-5D	EuroQol
FSS	Fatigue Severity Scale
FSH	Follicular Stimulating Hormone
GCP	Good Clinical Practice
GGT	gamma-glutamyl transferase
GM-CSF	granulocyte-macrophage colony-stimulating factor
GT	Genotype
HBsAg	hepatitis B surface antigen
HBV	hepatitis B virus
HCC	hepatocellular carcinoma
hCG	β-human chorionic gonadotropin
Hct	Hematocrit
HCV	hepatitis C virus
Hgb or Hg	Hemoglobin
HDL	high density lipoprotein
HDV	hepatitis D virus
HPSS-D	Hepatitis Physical Symptom Severity Diary
HIV	human immunodeficiency virus
IB	Investigator Brochure

Approved v4.0 930056810 4.0

Term	Definition
IC	informed consent
IC_{50}	half maximal inhibitory concentration
ICH	International Conference on Harmonization
IFN	Interferon
IEC	Independent Ethics Committee
IL	interleukin
IP	investigational product
IRB	Institutional Review Board
ISGs	interferon stimulated genes
ITT	intent to treat
IU	international unit
IVRS	interactive voice response system
Kg	Kilogram
Lambda	peginterferon lambda-1a
LDH	lactate dehydrogenase
LDL	low density lipoprotein
LLOQ	limit of quantitation
LLN	lower limit of normal
MedDRA	Medical Dictionary for Regulatory Activities
Mg	Milligram
mITT	modified intent to treat
mL	Milliliter
MRI	magnetic resonance imaging
NDA	New Drug Application
NE	norethindrone
Ng	Nanogram
OCP	oral contraceptive pill
pDILI	potential drug-induced liver injury
Peg	polyethylene glycol
PEG-rIL-29	PEGylated recombinant interleukin 29
PHQ-9	Patient Health Questionnaire-9

Approved v4.0 930056810 4.0

Term	Definition
PK	Pharmacokinetics
PRO	patient-reported outcomes
PT	prothrombin time
PTT	partial prothrombin time
QD	once daily
RBC	red blood cells (count)
RBV	ribavirin
RGT	response-guided therapy
rIL	recombinant human interleukin
RNA	ribonucleic acid
RVR	rapid virologic response
SAE	serious adverse event
SC	subcutaneous
SmPC	Summary of Product Characteristics
SNP	single nucleotide polymorphisms
SOC	standard of care
STAT	signal transducing activator of transcription
SVR	sustained virologic response
TID	three times a day
Tmax	time of maximum observed serum/plasma concentration
TSH	thyroid-stimulating hormone
TVR	telaprevir
µg	microgram
ULN	upper limit of normal
WBC	white blood cells (count)
WOCBP	women of childbearing potential

Approved v4.0 930056810 4.0

12 REFERENCES

[1] Hepatitis C. (Accessed at http://www.who.int/immunization/topics/hepatitis_c/en/

[2] Alter MJ. Epidemiology of hepatitis C in the West. Semin Liver Dis 1995;15:5-14

[3] Trepo C, Pradat P. Hepatitis C virus infection in Western Europe. J Hepatol 1999;31 Suppl 1:80-3

[4] Naoumov NV. Hepatitis C virus infection in Eastern Europe. J Hepatol 1999;31 Suppl 1:84-7

[5] Armstrong GL, Wasley A, Simard EP, McQuillan GM, Kuhnert WL, Alter MJ. The prevalence of hepatitis C virus infection in the United States, 1999 through 2002. Ann Intern Med 2006;144:705-14.

[6] EASL. EASL International Consensus Conference on Hepatitis C. Paris, 26-28, February 1999, Consensus Statement. European Association for the Study of the Liver. J Hepatol 1999;30:956-61.

[7] Williams R. Global challenges in liver disease. Hepatology 2006;44:521-6.

[8] Villano SA, Vlahov D, Nelson KE, Cohn S, Thomas DL. Persistence of viremia and the importance of long-term follow-up after acute hepatitis C infection. Hepatology 1999;29:908-14.

[9] Freeman RB, Jr., Steffick DE, Guidinger MK, Farmer DG, Berg CL, Merion RM. Liver and intestine transplantation in the United States, 1997-2006. Am J Transplant 2008;8:958-76.

[10] Thuluvath PJ, Guidinger MK, Fung JJ, Johnson LB, Rayhill SC, Pelletier SJ. Liver transplantation in the United States, 1999-2008. Am J Transplant 2010;10:1003-19.

[11] Marco daCosta DiBonaventura YY, Gilbert L'Italien, Ray Kim. The Impact of Hepatitis C on Health-Related Quality of Life, Work Productivity, and Healthcare Utilization. In: American Association for The Study of Liver Disease (AASLD). Boston, USA; 2010.

[12] Spiegel BM, Younossi ZM, Hays RD, Revicki D, Robbins S, Kanwal F. Impact of hepatitis C on health related quality of life: a systematic review and quantitative assessment. Hepatology 2005;41:790-800.

[13] Davis GL, Albright JE, Cook SF, Rosenberg DM. Projecting future complications of chronic hepatitis C in the United States. Liver Transpl 2003;9:331-8.

[14] Wong JB, McQuillan GM, McHutchison JG, Poynard T. Estimating future hepatitis C morbidity, mortality, and costs in the United States. Am J Public Health 2000;90:1562-9.

[15] Deuffic-Burban S, Poynard T, Sulkowski MS, Wong JB. Estimating the future health burden of chronic hepatitis C and human immunodeficiency virus infections in the United States. J Viral Hepat 2007;14:107-15.

[16] GHANY, M.G., STRADER, D. B., THOMAS, D. L. & SEEFF, L. B. 2009. Diagnosis, management, and treatment of hepatitis C: an update. Hepatology, 49, 1335-74.)

Approved v4.0 930056810 4.0

17 von Hahn T, Yoon JC, Alter H, et al. Hepatitis C virus continuously escapes from neutralizing antibody and T-cell responses during chronic infection in vivo. Gastroenterology 2007;132:667-78

18 Kuiken C, Simmonds P. Nomenclature and numbering of the hepatitis C virus. Methods Mol Biol 2009;510:33-53

19 Bostan N, Mahmood T. An overview about hepatitis C: a devastating virus. Crit Rev Microbiol 2010;36:91-133

20 Craxi A. EASL Clinical Practice Guidelines: Management of hepatitis C virus infection. J Hepatol 2011

21 Ghany MG, Nelson DR, Strader DB, Thomas DL, Seeff LB. An update on treatment of genotype 1 chronic hepatitis C virus infection: 2011 practice guideline by the American Association for the Study of Liver Diseases. Hepatology 2011;54:1433-44.

22 Fried MW, Shiffman ML, Reddy KR, et al. Peginterferon alfa-2a plus ribavirin for chronic hepatitis C virus infection. N Engl J Med 2002;347:975-82

23 Hadziyannis SJ, Sette H, Jr., Morgan TR, et al. Peginterferon-alpha2a and ribavirin combination therapy in chronic hepatitis C: a randomized study of treatment duration and ribavirin dose. Ann Intern Med 2004;140:346-55

24 Romero-Gomez M, Del Mar Viloria M, Andrade RJ, et al. Insulin resistance impairs sustained response rate to peginterferon plus ribavirin in chronic hepatitis C patients. Gastroenterology 2005;128:636-41.

25 Ferenci P, Fried MW, Shiffman ML, et al. Predicting sustained virologic responses in chronic hepatitis C patients treated with peginterferon alfa-2a (40 KD)/ribavirin. J Hepatol 2005;43:425-33

26 Jensen DM, Morgan TR, Marcellin P, et al. Early identification of HCV genotype 1 patients responding to 24 weeks peginterferon alpha-2a (40 kd)/ribavirin therapy. Hepatology 2006;43:954-60

27 VICTRELIS (Boceprevir)-US Label; 2011

28 Poordad F, McCone J, Jr., Bacon BR, et al. Boceprevir for untreated chronic HCV genotype 1 infection. N Engl J Med 2011;364:1195-206

29 Gish RG. Treating hepatitis C: the state of the art. Gastroenterol Clin North Am 2004;33:S1-9

30 Hashemi N, Rossi S, Navarro VJ, Herrine SK. Safety of peginterferon in the treatment of chronic hepatitis C. Expert Opin Drug Saf 2008;7:771-81

Approved v4.0 930056810 4.0

[31] Strader DB, Wright T, Thomas DL, Seeff LB. Diagnosis, management, and treatment of hepatitis C. Hepatology 2004;39:1147-71; Kowdley KV. Hematologic side effects of interferon and ribavirin therapy. J Clin Gastroenterol 2005;39:S3-8

[32] Kowdley KV. Hematologic side effects of interferon and ribavirin therapy. J Clin Gastroenterol 2005;39:S3-8

[33] Soriano V, Vispo E, Poveda E, Labarga P, Barreiro P. Treatment failure with new hepatitis C drugs. Expert Opin Pharmacother 2012;13:313-23

[34] Marcellin P, Chousterman M, Fontanges T, et al. Adherence to treatment and quality of life during hepatitis C therapy: a prospective, real-life, observational study. Liver Int 2011;31:516-24

[35] Pearlman BL. Hepatitis C treatment update. Am J Med 2004;117:344-52

[36] McHutchison JG, Manns M, Patel K, et al. Adherence to combination therapy enhances sustained response in genotype-1-infected patients with chronic hepatitis C. Gastroenterology 2002;123:1061-9

[37] Determination of the inhibition or induction potential of PEG-rIL-29 on liver cytochrome P450 metabolism. ZymoGenetics Report RES-10773; 2005. Bristol-Myers Squibb document control number 93004240

[38] Freeman JA HM, Gray T, Lopez-Talavera JC, Horga M, Fontana D, Hillson J, Kansra V, Wind-Rotolo M. The Effect of Pegylated Interferon Lambda on the Expression of Interferon-Stimulated Genes in Whole Blood in Chronic Hepatitis C Patients in a Phase 2a Study. In: AASLD. San Francisco, USA; 2011

[39] Hezode C, Fontaine H, Dorival C et al. Triple therapy in treatment-experienced patients with HCV-cirrhosis in a multi-centre cohort of the French early access programme. J Hepatol 2013 (in press), http://dx.doi.org/10.1016/j.jhep.2013.04.035.

[40] Garcia-Tsao G, Friedman S, Iredale J, Pinzani M. Now there are many (stages) where before there wa one: in search of a pathophysiological classification of cirrhosis. Hepatology 2010;51:1445-1449.

[41] Berzigotti A, Seijo S, Arena U et al. Elastography, spleen size, and platelet count identify portal hypertension in patients with coompensated cirrhosis. Gastroenterology 2013;144:103-111.

[42] Robic MA, Procopet B, Metivier S et al. Liver stiffness accurately predicts portal hypertension related complications in patients which chronic liver disease: a prospective study. J Hepatol 2011;55:1017-1024.

[43] ZIOL, M., HANDRA-LUCA, A., KETTANEH, A., CHRISTIDIS, C., MAL, F., KAZEMI, F., DE LEDINGHEN, V., MARCELLIN, P., DHUMEAUX, D., TRINCHET, J. C. & BEAUGRAND, M. 2005. Noninvasive assessment of liver fibrosis by measurement of stiffness in patients with chronic hepatitis C. Hepatology, 41, 48-54.

Approved v4.0 930056810 4.0

44 Pegylated Interferon Lambda (Lambda) Investigator Brochure (Version 9). ZymoGenetics; April 2012.

45 Pegasys (peginterferon alfa-2a) U.S. Package Insert. Roche Pharmaceuticals. October 2008.

46 Copegus (ribavirin) U.S. Package Insert. Roche Pharmaceuticals. April 2008. Pegasys (peginterferon alfa-2a) U.S. Package Insert. Roche Pharmaceuticals. October 2008.

47 Pegasys (peginterferon alfa-2a) Summary of Product Characteristics. Roche Pharmaceuticals. July 2010.

48 Copegus (ribavirin) Summary of Product Characteristics. Roche Pharmaceuticals. April 2010.

49 INCIVEKÔ (Telaprevir) Summary of Product Characteristics. Vertex Pharmaceuticals Incorporated. September 2011.

Approved v4.0 930056810 4.0

APPENDIX 1 DSM IV: DIAGNOSTIC CRITERIA FOR DRUG AND ALCOHOL ABUSE

Criteria for Alcohol & Substance Abuse

1. A maladaptive pattern of substance use leading to clinically significant impairment or distress, as manifested by one (or more) of the following, occurring within a 12-month period:
   a) recurrent substance use resulting in a failure to fulfill major role obligations at work, school, or home (eg, repeated absences or poor work performance related to substance use; substance-related absences, suspensions, or expulsions from school; neglect of children or household)
   b) recurrent substance use in situations in which it is physically hazardous (eg, driving an automobile or operating a machine when impaired by substance use)
   c) recurrent substance-related legal problems (eg, arrests for substance-related disorderly conduct)
   d) continued substance use despite having persistent or recurrent social or interpersonal problems caused or exacerbated by the effects of the substance (eg, arguments with spouse about consequences of intoxication, physical fights)
2. The symptoms have never met the criteria for Substance Dependence for this class of substance.

Approved v4.0 930056810 4.0

APPENDIX 2 DIVISION OF AIDS TABLE FOR GRADING THE SEVERITY OF ADULT AND PEDIATRIC ADVERSE EVENTS PUBLISH DATE: DECEMBER, 2004

DIVISION OF AIDS TABLE FOR GRADING THE SEVERITY OF ADULT AND PEDIATRIC ADVERSE EVENTS
VERSION 1.0, DECEMBER, 2004; CLARIFICATION AUGUST 2009

The Division of AIDS Table for Grading the Severity of Adult and Pediatric Adverse Events ("DAIDS AE Grading Table") is a descriptive terminology which can be utilized for Adverse Event (AE) reporting. A grading (severity) scale is provided for each AE term.

This clarification of the DAIDS Table for Grading the Severity of Adult and Pediatric AE's provides additional explanation of the DAIDS AE Grading Table and clarifies some of the parameters.

I. Instructions and Clarifications

Grading Adult and Pediatric AEs

The DAIDS AE Grading Table includes parameters for grading both Adult and Pediatric AEs. When a single set of parameters is not appropriate for grading specific types of AEs for both Adult and Pediatric populations, separate sets of parameters for Adult and/or Pediatric populations (with specified respective age ranges) are given in the Table. If there is no distinction in the Table between Adult and Pediatric values for a type of AE, then the single set of parameters listed is to be used for grading the severity of both Adult and Pediatric events of that type.

> **Note:** In the classification of adverse events, the term "**severe**" is not the same as "**serious**." Severity is an indication of the intensity of a specific event (as in mild, moderate, or severe chest pain). The term "**serious**" relates to a participant/event outcome or action criteria, usually associated with events that pose a threat to a participant's life or functioning.

Addenda 1-3 Grading Tables for Microbicide Studies

For protocols involving topical application of products to the female genital tract, male genital area or rectum, strong consideration should be given to using Appendices I-III as the primary grading scales for these areas. The protocol would need to specifically state that one or more of the Appendices would be primary (and thus take precedence over the main Grading Table) for items that are listed in both the Appendix and the main Grading Table.

- Addendum 1 - Female Genital Grading Table for Use in Microbicide Studies - PDF
- Addendum 2 - Male Genital Grading Table for Use in Microbicide Studies - PDF
- Addendum 3 - Rectal Grading Table for Use in Microbicide Studies - PDF

Grade 5

For any AE where the outcome is death, the severity of the AE is classified as Grade 5.

Estimating Severity Grade for Parameters Not Identified in the Table

In order to grade a clinical AE that is not identified in the DAIDS AE grading table, use the category "Estimating Severity Grade" located on Page 3.

Determining Severity Grade for Parameters "Between Grades"

If the severity of a clinical AE could fall under either one of two grades (e.g., the severity of an AE could be either Grade 2 or Grade 3), select the higher of the two grades for the AE. If a laboratory value that is graded as a multiple of the ULN or LLN falls between two grades, select the higher of the two grades for the AE. For example, Grade 1 is 2.5 x ULN and Grade 2 is 2.6 x ULN for a parameter. If the lab value is 2.53 x ULN (which is between the two grades), the severity of this AE would be Grade 2, the higher of the two grades.

Values Below Grade 1

Any laboratory value that is between either the LLN or ULN and Grade 1 should not be graded.

Approved v4.0 930056810 4.0

DIVISION OF AIDS TABLE FOR GRADING THE SEVERITY OF ADULT AND PEDIATRIC ADVERSE EVENTS
VERSION 1.0, DECEMBER, 2004; CLARIFICATION AUGUST 2009

Determining Severity Grade when Local Laboratory Normal Values Overlap with Grade 1 Ranges
In these situations, the severity grading is based on the ranges in the DAIDS AE Grading Table, even when there is a reference to the local lab LLN.

For example: Phosphate, Serum, Low, Adult and Pediatric > 14 years (Page 20) Grade 1 range is 2.50 mg/dL - < LLN. A particular laboratory's normal range for Phosphate is 2.1 – 3.8 mg/dL. A participant's actual lab value is 2.5. In this case, the value of 2.5 exceeds the LLN for the local lab, but will be graded as Grade 1 per DAIDS AE Grading Table.

.

II. Definitions of terms used in the Table:

Basic Self-care Functions	Adult<br>Activities such as bathing, dressing, toileting, transfer/movement, continence, and feeding.<br><br>Young Children<br>Activities that are age and culturally appropriate (e.g., feeding self with culturally appropriate eating implement).
LLN	Lower limit of normal
Medical Intervention	Use of pharmacologic or biologic agent(s) for treatment of an AE.
NA	Not Applicable
Operative Intervention	Surgical OR other invasive mechanical procedures.
ULN	Upper limit of normal
Usual Social & Functional Activities	Adult<br>Adaptive tasks and desirable activities, such as going to work, shopping, cooking, use of transportation, pursuing a hobby, etc.<br><br>Young Children<br>Activities that are age and culturally appropriate (e.g., social interactions, play activities, learning tasks, etc.).

Approved v4.0 930056810 4.0

DIVISION OF AIDS TABLE FOR GRADING THE SEVERITY OF ADULT AND PEDIATRIC ADVERSE EVENTS
VERSION 1.0, DECEMBER, 2004; CLARIFICATION AUGUST 2009

PARAMETER	GRADE 1 MILD	GRADE 2 MODERATE	GRADE 3 SEVERE	GRADE 4 POTENTIALLY LIFE-THREATENING
**ESTIMATING SEVERITY GRADE**				
Clinical adverse event NOT identified elsewhere in this DAIDS AE Grading Table	Symptoms causing no or minimal interference with usual social & functional activities	Symptoms causing greater than minimal interference with usual social & functional activities	Symptoms causing inability to perform usual social & functional activities	Symptoms causing inability to perform basic self-care functions OR Medical or operative intervention indicated to prevent permanent impairment, persistent disability, or death
**SYSTEMIC**				
Acute systemic allergic reaction	Localized urticaria (wheals) with no medical intervention indicated	Localized urticaria with medical intervention indicated OR Mild angioedema with no medical intervention indicated	Generalized urticaria OR Angioedema with medical intervention indicated OR Symptomatic mild bronchospasm	Acute anaphylaxis OR Life-threatening bronchospasm OR laryngeal edema
Chills	Symptoms causing no or minimal interference with usual social & functional activities	Symptoms causing greater than minimal interference with usual social & functional activities	Symptoms causing inability to perform usual social & functional activities	NA
Fatigue<br>Malaise	Symptoms causing no or minimal interference with usual social & functional activities	Symptoms causing greater than minimal interference with usual social & functional activities	Symptoms causing inability to perform usual social & functional activities	Incapacitating fatigue/ malaise symptoms causing inability to perform basic self-care functions
Fever (nonaxillary)	37.7 – 38.6°C	38.7 – 39.3°C	39.4 – 40.5°C	> 40.5°C
Pain (indicate body site)<br>DO NOT use for pain due to injection (See Injection Site Reactions: Injection site pain)<br>See also Headache, Arthralgia, and Myalgia	Pain causing no or minimal interference with usual social & functional activities	Pain causing greater than minimal interference with usual social & functional activities	Pain causing inability to perform usual social & functional activities	Disabling pain causing inability to perform basic self-care functions OR Hospitalization (other than emergency room visit) indicated

Basic Self-care Functions – Adult: Activities such as bathing, dressing, toileting, transfer/movement, continence, and feeding.

Basic Self-care Functions – Young Children: Activities that are age and culturally appropriate (e.g., feeding self with culturally appropriate eating implement).

Usual Social & Functional Activities – Adult: Adaptive tasks and desirable activities, such as going to work, shopping, cooking, use of transportation, pursuing a hobby, etc.

Usual Social & Functional Activities – Young Children: Activities that are age and culturally appropriate (e.g., social interactions, play activities, learning tasks, etc.).

Approved v4.0 930056810 4.0

DIVISION OF AIDS TABLE FOR GRADING THE SEVERITY OF ADULT AND PEDIATRIC ADVERSE EVENTS
VERSION 1.0, DECEMBER, 2004; CLARIFICATION AUGUST 2009

PARAMETER	GRADE 1 MILD	GRADE 2 MODERATE	GRADE 3 SEVERE	GRADE 4 POTENTIALLY LIFE-THREATENING
Unintentional weight loss	NA	5 – 9% loss in body weight from baseline	10 – 19% loss in body weight from baseline	≥ 20% loss in body weight from baseline OR Aggressive intervention indicated [e.g., tube feeding or total parenteral nutrition (TPN)]
**INFECTION**				
Infection (any other than HIV infection)	Localized, no systemic antimicrobial treatment indicated AND Symptoms causing no or minimal interference with usual social & functional activities	Systemic antimicrobial treatment indicated OR Symptoms causing greater than minimal interference with usual social & functional activities	Systemic antimicrobial treatment indicated AND Symptoms causing inability to perform usual social & functional activities OR Operative intervention (other than simple incision and drainage) indicated	Life-threatening consequences (e.g., septic shock)
**INJECTION SITE REACTIONS**				
Injection site pain (pain without touching) Or Tenderness (pain when area is touched)	Pain/tenderness causing no or minimal limitation of use of limb	Pain/tenderness limiting use of limb OR Pain/tenderness causing greater than minimal interference with usual social & functional activities	Pain/tenderness causing inability to perform usual social & functional activities	Pain/tenderness causing inability to perform basic self-care function OR Hospitalization (other than emergency room visit) indicated for management of pain/tenderness
Injection site reaction (localized)				
**Adult > 15 years**	Erythema OR Induration of 5x5 cm – 9x9 cm (or 25 cm^2 – 81cm^2)	Erythema OR Induration OR Edema > 9 cm any diameter (or > 81 cm^2)	Ulceration OR Secondary infection OR Phlebitis OR Sterile abscess OR Drainage	Necrosis (involving dermis and deeper tissue)
**Pediatric ≤ 15 years**	Erythema OR Induration OR Edema present but ≤ 2.5 cm diameter	Erythema OR Induration OR Edema > 2.5 cm diameter but < 50% surface area of the extremity segment (e.g., upper arm/thigh)	Erythema OR Induration OR Edema involving ≥ 50% surface area of the extremity segment (e.g., upper arm/thigh) OR Ulceration OR Secondary infection OR Phlebitis OR Sterile abscess OR Drainage	Necrosis (involving dermis and deeper tissue)

Basic Self-care Functions – Adult: Activities such as bathing, dressing, toileting, transfer/movement, continence, and feeding.

Basic Self-care Functions – Young Children: Activities that are age and culturally appropriate (e.g., feeding self with culturally appropriate eating implement).

Usual Social & Functional Activities – Adult: Adaptive tasks and desirable activities, such as going to work, shopping, cooking, use of transportation, pursuing a hobby, etc.

Usual Social & Functional Activities – Young Children: Activities that are age and culturally appropriate (e.g., social interactions, play activities, learning tasks, etc.).

28 Dec 04/Clarification Aug 09 Page 4 of 21 Version 1.0/Clarification 1

Approved v4.0 930056810 4.0

DIVISION OF AIDS TABLE FOR GRADING THE SEVERITY OF ADULT AND PEDIATRIC ADVERSE EVENTS
VERSION 1.0, DECEMBER, 2004; CLARIFICATION AUGUST 2009

PARAMETER	GRADE 1 MILD	GRADE 2 MODERATE	GRADE 3 SEVERE	GRADE 4 POTENTIALLY LIFE-THREATENING
Pruritis associated with injection<br>See also Skin: Pruritis (itching - no skin lesions)	Itching localized to injection site AND Relieved spontaneously or with < 48 hours treatment	Itching beyond the injection site but not generalized OR Itching localized to injection site requiring ≥ 48 hours treatment	Generalized itching causing inability to perform usual social & functional activities	NA
**SKIN – DERMATOLOGICAL**				
Alopecia	Thinning detectable by study participant (or by caregiver for young children and disabled adults)	Thinning or patchy hair loss detectable by health care provider	Complete hair loss	NA
Cutaneous reaction – rash	Localized macular rash	Diffuse macular, maculopapular, or morbilliform rash OR Target lesions	Diffuse macular, maculopapular, or morbilliform rash with vesicles or limited number of bullae OR Superficial ulcerations of mucous membrane limited to one site	Extensive or generalized bullous lesions OR Stevens-Johnson syndrome OR Ulceration of mucous membrane involving two or more distinct mucosal sites OR Toxic epidermal necrolysis (TEN)
Hyperpigmentation	Slight or localized	Marked or generalized	NA	NA
Hypopigmentation	Slight or localized	Marked or generalized	NA	NA
Pruritis (itching – no skin lesions)<br>(See also Injection Site Reactions: Pruritis associated with injection)	Itching causing no or minimal interference with usual social & functional activities	Itching causing greater than minimal interference with usual social & functional activities	Itching causing inability to perform usual social & functional activities	NA
**CARDIOVASCULAR**				
Cardiac arrhythmia (general)<br>(By ECG or physical exam)	Asymptomatic AND No intervention indicated	Asymptomatic AND Non-urgent medical intervention indicated	Symptomatic, non-life-threatening AND Non-urgent medical intervention indicated	Life-threatening arrhythmia OR Urgent intervention indicated
Cardiac-ischemia/infarction	NA	NA	Symptomatic ischemia (stable angina) OR Testing consistent with ischemia	Unstable angina OR Acute myocardial infarction

Basic Self-care Functions – Adult: Activities such as bathing, dressing, toileting, transfer/movement, continence, and feeding.

Basic Self-care Functions – Young Children: Activities that are age and culturally appropriate (e.g., feeding self with culturally appropriate eating implement).

Usual Social & Functional Activities – Adult: Adaptive tasks and desirable activities, such as going to work, shopping, cooking, use of transportation, pursuing a hobby, etc.

Usual Social & Functional Activities – Young Children: Activities that are age and culturally appropriate (e.g., social interactions, play activities, learning tasks, etc.).

28 Dec 04/Clarification Aug 09 Page 5 of 21 Version 1.0/Clarification 1

Approved v4.0 930056810 4.0

DIVISION OF AIDS TABLE FOR GRADING THE SEVERITY OF ADULT AND PEDIATRIC ADVERSE EVENTS
VERSION 1.0, DECEMBER, 2004; CLARIFICATION AUGUST 2009

PARAMETER	GRADE 1 MILD	GRADE 2 MODERATE	GRADE 3 SEVERE	GRADE 4 POTENTIALLY LIFE-THREATENING
Hemorrhage (significant acute blood loss)	NA	Symptomatic AND No transfusion indicated	Symptomatic AND Transfusion of ≤ 2 units packed RBCs (for children ≤ 10 cc/kg) indicated	Life-threatening hypotension OR Transfusion of > 2 units packed RBCs (for children > 10 cc/kg) indicated
Hypertension				
**Adult > 17 years** (with repeat testing at same visit)	140 – 159 mmHg systolic OR 90 – 99 mmHg diastolic	160 – 179 mmHg systolic OR 100 – 109 mmHg diastolic	≥ 180 mmHg systolic OR ≥ 110 mmHg diastolic	Life-threatening consequences (e.g., malignant hypertension) OR Hospitalization indicated (other than emergency room visit)
**Correction**: in Grade 2 to 160 - 179 from > 160-179 (systolic) and to ≥ 100 -109 from > 100-109 (diastolic) and in Grade 3 to ≥ 180 from > 180 (systolic) and to ≥ 110 from > 110 (diastolic).				
**Pediatric ≤ 17 years** (with repeat testing at same visit)	NA	91st – 94th percentile adjusted for age, height, and gender (systolic and/or diastolic)	≥ 95th percentile adjusted for age, height, and gender (systolic and/or diastolic)	Life-threatening consequences (e.g., malignant hypertension) OR Hospitalization indicated (other than emergency room visit)
Hypotension	NA	Symptomatic, corrected with oral fluid replacement	Symptomatic, IV fluids indicated	Shock requiring use of vasopressors or mechanical assistance to maintain blood pressure
Pericardial effusion	Asymptomatic, small effusion requiring no intervention	Asymptomatic, moderate or larger effusion requiring no intervention	Effusion with non-life threatening physiologic consequences OR Effusion with non-urgent intervention indicated	Life-threatening consequences (e.g., tamponade) OR Urgent intervention indicated
Prolonged PR interval				
**Adult > 16 years**	PR interval 0.21 – 0.25 sec	PR interval > 0.25 sec	Type II 2nd degree AV block OR Ventricular pause > 3.0 sec	Complete AV block
**Pediatric ≤ 16 years**	1st degree AV block (PR > normal for age and rate)	Type I 2nd degree AV block	Type II 2nd degree AV block	Complete AV block

Basic Self-care Functions – Adult: Activities such as bathing, dressing, toileting, transfer/movement, continence, and feeding.

Basic Self-care Functions – Young Children: Activities that are age and culturally appropriate (e.g., feeding self with culturally appropriate eating implement).

Usual Social & Functional Activities – Adult: Adaptive tasks and desirable activities, such as going to work, shopping, cooking, use of transportation, pursuing a hobby, etc.

Usual Social & Functional Activities – Young Children: Activities that are age and culturally appropriate (e.g., social interactions, play activities, learning tasks, etc.).

Approved v4.0 930056810 4.0

DIVISION OF AIDS TABLE FOR GRADING THE SEVERITY OF ADULT AND PEDIATRIC ADVERSE EVENTS
VERSION 1.0, DECEMBER, 2004; CLARIFICATION AUGUST 2009

PARAMETER	GRADE 1 MILD	GRADE 2 MODERATE	GRADE 3 SEVERE	GRADE 4 POTENTIALLY LIFE-THREATENING
Prolonged QTc				
**Adult > 16 years**	Asymptomatic, QTc interval 0.45 – 0.47 sec OR Increase interval < 0.03 sec above baseline	Asymptomatic, QTc interval 0.48 – 0.49 sec OR Increase in interval 0.03 – 0.05 sec above baseline	Asymptomatic, QTc interval ≥ 0.50 sec OR Increase in interval ≥ 0.06 sec above baseline	Life-threatening consequences, e.g. Torsade de pointes or other associated serious ventricular dysrhythmia
**Pediatric ≤ 16 years**	Asymptomatic, QTc interval 0.450 – 0.464 sec	Asymptomatic, QTc interval 0.465 – 0.479 sec	Asymptomatic, QTc interval ≥ 0.480 sec	Life-threatening consequences, e.g. Torsade de pointes or other associated serious ventricular dysrhythmia
Thrombosis/embolism	NA	Deep vein thrombosis AND No intervention indicated (e.g., anticoagulation, lysis filter, invasive procedure)	Deep vein thrombosis AND Intervention indicated (e.g., anticoagulation, lysis filter, invasive procedure)	Embolic event (e.g., pulmonary embolism, life-threatening thrombus)
Vasovagal episode (associated with a procedure of any kind)	Present without loss of consciousness	Present with transient loss of consciousness	NA	NA
Ventricular dysfunction (congestive heart failure)	NA	Asymptomatic diagnostic finding AND intervention indicated	New onset with symptoms OR Worsening symptomatic congestive heart failure	Life-threatening congestive heart failure
**GASTROINTESTINAL**				
Anorexia	Loss of appetite without decreased oral intake	Loss of appetite associated with decreased oral intake without significant weight loss	Loss of appetite associated with significant weight loss	Life-threatening consequences OR Aggressive intervention indicated [e.g., tube feeding or total parenteral nutrition (TPN)]
**Comment**: Please note that, while the grading scale provided for Unintentional Weight Loss may be used as a guideline when grading anorexia, this is not a requirement and should not be used as a substitute for clinical judgment.				
Ascites	Asymptomatic	Symptomatic AND Intervention indicated (e.g., diuretics or therapeutic paracentesis)	Symptomatic despite intervention	Life-threatening consequences

Basic Self-care Functions – Adult: Activities such as bathing, dressing, toileting, transfer/movement, continence, and feeding.

Basic Self-care Functions – Young Children: Activities that are age and culturally appropriate (e.g., feeding self with culturally appropriate eating implement).

Usual Social & Functional Activities – Adult: Adaptive tasks and desirable activities, such as going to work, shopping, cooking, use of transportation, pursuing a hobby, etc.

Usual Social & Functional Activities – Young Children: Activities that are age and culturally appropriate (e.g., social interactions, play activities, learning tasks, etc.).

Approved v4.0 930056810 4.0

DIVISION OF AIDS TABLE FOR GRADING THE SEVERITY OF ADULT AND PEDIATRIC ADVERSE EVENTS
VERSION 1.0, DECEMBER, 2004; CLARIFICATION AUGUST 2009

PARAMETER	GRADE 1 MILD	GRADE 2 MODERATE	GRADE 3 SEVERE	GRADE 4 POTENTIALLY LIFE-THREATENING
Cholecystitis	NA	Symptomatic AND Medical intervention indicated	Radiologic, endoscopic, or operative intervention indicated	Life-threatening consequences (e.g., sepsis or perforation)
Constipation	NA	Persistent constipation requiring regular use of dietary modifications, laxatives, or enemas	Obstipation with manual evacuation indicated	Life-threatening consequences (e.g., obstruction)
Diarrhea				
**Adult and Pediatric ≥ 1 year**	Transient or intermittent episodes of unformed stools OR Increase of ≤ 3 stools over baseline per 24-hour period	Persistent episodes of unformed to watery stools OR Increase of 4 – 6 stools over baseline per 24-hour period	Bloody diarrhea OR Increase of ≥ 7 stools per 24-hour period OR IV fluid replacement indicated	Life-threatening consequences (e.g., hypotensive shock)
**Pediatric < 1 year**	Liquid stools (more unformed than usual) but usual number of stools	Liquid stools with increased number of stools OR Mild dehydration	Liquid stools with moderate dehydration	Liquid stools resulting in severe dehydration with aggressive rehydration indicated OR Hypotensive shock
Dysphagia-Odynophagia	Symptomatic but able to eat usual diet	Symptoms causing altered dietary intake without medical intervention indicated	Symptoms causing severely altered dietary intake with medical intervention indicated	Life-threatening reduction in oral intake
Mucositis/stomatitis (clinical exam)<br>Indicate site (e.g., larynx, oral)<br>See Genitourinary for Vulvovaginitis<br>See also Dysphagia-Odynophagia and Proctitis	Erythema of the mucosa	Patchy pseudomembranes or ulcerations	Confluent pseudomembranes or ulcerations OR Mucosal bleeding with minor trauma	Tissue necrosis OR Diffuse spontaneous mucosal bleeding OR Life-threatening consequences (e.g., aspiration, choking)
Nausea	Transient (< 24 hours) or intermittent nausea with no or minimal interference with oral intake	Persistent nausea resulting in decreased oral intake for 24 – 48 hours	Persistent nausea resulting in minimal oral intake for > 48 hours OR Aggressive rehydration indicated (e.g., IV fluids)	Life-threatening consequences (e.g., hypotensive shock)

Basic Self-care Functions – Adult: Activities such as bathing, dressing, toileting, transfer/movement, continence, and feeding.

Basic Self-care Functions – Young Children: Activities that are age and culturally appropriate (e.g., feeding self with culturally appropriate eating implement).

Usual Social & Functional Activities – Adult: Adaptive tasks and desirable activities, such as going to work, shopping, cooking, use of transportation, pursuing a hobby, etc.

Usual Social & Functional Activities – Young Children: Activities that are age and culturally appropriate (e.g., social interactions, play activities, learning tasks, etc.).

28 Dec 04/Clarification Aug 09 Page 8 of 21 Version 1.0/Clarification 1

Approved v4.0 930056810 4.0

DIVISION OF AIDS TABLE FOR GRADING THE SEVERITY OF ADULT AND PEDIATRIC ADVERSE EVENTS
VERSION 1.0, DECEMBER, 2004; CLARIFICATION AUGUST 2009

PARAMETER	GRADE 1 MILD	GRADE 2 MODERATE	GRADE 3 SEVERE	GRADE 4 POTENTIALLY LIFE-THREATENING
Pancreatitis	NA	Symptomatic AND Hospitalization not indicated (other than emergency room visit)	Symptomatic AND Hospitalization indicated (other than emergency room visit)	Life-threatening consequences (e.g., circulatory failure, hemorrhage, sepsis)
Proctitis (functional-symptomatic) Also see Mucositis/stomatitis for clinical exam	Rectal discomfort AND No intervention indicated	Symptoms causing greater than minimal interference with usual social & functional activities OR Medical intervention indicated	Symptoms causing inability to perform usual social & functional activities OR Operative intervention indicated	Life-threatening consequences (e.g., perforation)
Vomiting	Transient or intermittent vomiting with no or minimal interference with oral intake	Frequent episodes of vomiting with no or mild dehydration	Persistent vomiting resulting in orthostatic hypotension OR Aggressive rehydration indicated (e.g., IV fluids)	Life-threatening consequences (e.g., hypotensive shock)
**NEUROLOGIC**				
Alteration in personality-behavior or in mood (e.g., agitation, anxiety, depression, mania, psychosis)	Alteration causing no or minimal interference with usual social & functional activities	Alteration causing greater than minimal interference with usual social & functional activities	Alteration causing inability to perform usual social & functional activities	Behavior potentially harmful to self or others (e.g., suicidal and homicidal ideation or attempt, acute psychosis) OR Causing inability to perform basic self-care functions
Altered Mental Status For Dementia, see Cognitive and behavioral/attentional disturbance (including dementia and attention deficit disorder)	Changes causing no or minimal interference with usual social & functional activities	Mild lethargy or somnolence causing greater than minimal interference with usual social & functional activities	Confusion, memory impairment, lethargy, or somnolence causing inability to perform usual social & functional activities	Delirium OR obtundation, OR coma
Ataxia	Asymptomatic ataxia detectable on exam OR Minimal ataxia causing no or minimal interference with usual social & functional activities	Symptomatic ataxia causing greater than minimal interference with usual social & functional activities	Symptomatic ataxia causing inability to perform usual social & functional activities	Disabling ataxia causing inability to perform basic self-care functions

Basic Self-care Functions – Adult: Activities such as bathing, dressing, toileting, transfer/movement, continence, and feeding.

Basic Self-care Functions – Young Children: Activities that are age and culturally appropriate (e.g., feeding self with culturally appropriate eating implement).

Usual Social & Functional Activities – Adult: Adaptive tasks and desirable activities, such as going to work, shopping, cooking, use of transportation, pursuing a hobby, etc.

Usual Social & Functional Activities – Young Children: Activities that are age and culturally appropriate (e.g., social interactions, play activities, learning tasks, etc.).

28 Dec 04/Clarification Aug 09 Page 9 of 21 Version 1.0/Clarification 1

Approved v4.0 930056810 4.0

DIVISION OF AIDS TABLE FOR GRADING THE SEVERITY OF ADULT AND PEDIATRIC ADVERSE EVENTS
VERSION 1.0, DECEMBER, 2004; CLARIFICATION AUGUST 2009

PARAMETER	GRADE 1 MILD	GRADE 2 MODERATE	GRADE 3 SEVERE	GRADE 4 POTENTIALLY LIFE-THREATENING
Cognitive and behavioral/attentional disturbance (including dementia and attention deficit disorder)	Disability causing no or minimal interference with usual social & functional activities OR Specialized resources not indicated	Disability causing greater than minimal interference with usual social & functional activities OR Specialized resources on part-time basis indicated	Disability causing inability to perform usual social & functional activities OR Specialized resources on a full-time basis indicated	Disability causing inability to perform basic self-care functions OR Institutionalization indicated
CNS ischemia (acute)	NA	NA	Transient ischemic attack	Cerebral vascular accident (CVA, stroke) with neurological deficit
Developmental delay – **Pediatric ≤ 16 years**	Mild developmental delay, either motor or cognitive, as determined by comparison with a developmental screening tool appropriate for the setting	Moderate developmental delay, either motor or cognitive, as determined by comparison with a developmental screening tool appropriate for the setting	Severe developmental delay, either motor or cognitive, as determined by comparison with a developmental screening tool appropriate for the setting	Developmental regression, either motor or cognitive, as determined by comparison with a developmental screening tool appropriate for the setting
Headache	Symptoms causing no or minimal interference with usual social & functional activities	Symptoms causing greater than minimal interference with usual social & functional activities	Symptoms causing inability to perform usual social & functional activities	Symptoms causing inability to perform basic self-care functions OR Hospitalization indicated (other than emergency room visit) OR Headache with significant impairment of alertness or other neurologic function
Insomnia	NA	Difficulty sleeping causing greater than minimal interference with usual social & functional activities	Difficulty sleeping causing inability to perform usual social & functional activities	Disabling insomnia causing inability to perform basic self-care functions
Neuromuscular weakness (including myopathy & neuropathy)	Asymptomatic with decreased strength on exam OR Minimal muscle weakness causing no or minimal interference with usual social & functional activities	Muscle weakness causing greater than minimal interference with usual social & functional activities	Muscle weakness causing inability to perform usual social & functional activities	Disabling muscle weakness causing inability to perform basic self-care functions OR Respiratory muscle weakness impairing ventilation

Basic Self-care Functions – Adult: Activities such as bathing, dressing, toileting, transfer/movement, continence, and feeding.

Basic Self-care Functions – Young Children: Activities that are age and culturally appropriate (e.g., feeding self with culturally appropriate eating implement).

Usual Social & Functional Activities – Adult: Adaptive tasks and desirable activities, such as going to work, shopping, cooking, use of transportation, pursuing a hobby, etc.

Usual Social & Functional Activities – Young Children: Activities that are age and culturally appropriate (e.g., social interactions, play activities, learning tasks, etc.).

Approved v4.0 930056810 4.0

DIVISION OF AIDS TABLE FOR GRADING THE SEVERITY OF ADULT AND PEDIATRIC ADVERSE EVENTS
VERSION 1.0, DECEMBER, 2004; CLARIFICATION AUGUST 2009

PARAMETER	GRADE 1 MILD	GRADE 2 MODERATE	GRADE 3 SEVERE	GRADE 4 POTENTIALLY LIFE-THREATENING
Neurosensory alteration (including paresthesia and painful neuropathy)	Asymptomatic with sensory alteration on exam or minimal paresthesia causing no or minimal interference with usual social & functional activities	Sensory alteration or paresthesia causing greater than minimal interference with usual social & functional activities	Sensory alteration or paresthesia causing inability to perform usual social & functional activities	Disabling sensory alteration or paresthesia causing inability to perform basic self-care functions
Seizure: (new onset) **– Adult ≥ 18 years** See also Seizure: (known pre-existing seizure disorder)	NA	1 seizure	2 – 4 seizures	Seizures of any kind which are prolonged, repetitive (e.g., status epilepticus), or difficult to control (e.g., refractory epilepsy)
Seizure: (known pre-existing seizure disorder) **– Adult ≥ 18 years** For worsening of existing epilepsy the grades should be based on an increase from previous level of control to any of these levels.	NA	Increased frequency of pre-existing seizures (non-repetitive) without change in seizure character OR Infrequent break-through seizures while on stable medication in a previously controlled seizure disorder	Change in seizure character from baseline either in duration or quality (e.g., severity or focality)	Seizures of any kind which are prolonged, repetitive (e.g., status epilepticus), or difficult to control (e.g., refractory epilepsy)
Seizure **– Pediatric < 18 years**	Seizure, generalized onset with or without secondary generalization, lasting < 5 minutes with < 24 hours post ictal state	Seizure, generalized onset with or without secondary generalization, lasting 5 – 20 minutes with < 24 hours post ictal state	Seizure, generalized onset with or without secondary generalization, lasting > 20 minutes	Seizure, generalized onset with or without secondary generalization, requiring intubation and sedation
Syncope (not associated with a procedure)	NA	Present	NA	NA
Vertigo	Vertigo causing no or minimal interference with usual social & functional activities	Vertigo causing greater than minimal interference with usual social & functional activities	Vertigo causing inability to perform usual social & functional activities	Disabling vertigo causing inability to perform basic self-care functions

Basic Self-care Functions – Adult: Activities such as bathing, dressing, toileting, transfer/movement, continence, and feeding.

Basic Self-care Functions – Young Children: Activities that are age and culturally appropriate (e.g., feeding self with culturally appropriate eating implement).

Usual Social & Functional Activities – Adult: Adaptive tasks and desirable activities, such as going to work, shopping, cooking, use of transportation, pursuing a hobby, etc.

Usual Social & Functional Activities – Young Children: Activities that are age and culturally appropriate (e.g., social interactions, play activities, learning tasks, etc.).

Approved v4.0 930056810 4.0

DIVISION OF AIDS TABLE FOR GRADING THE SEVERITY OF ADULT AND PEDIATRIC ADVERSE EVENTS
VERSION 1.0, DECEMBER, 2004; CLARIFICATION AUGUST 2009

PARAMETER	GRADE 1 MILD	GRADE 2 MODERATE	GRADE 3 SEVERE	GRADE 4 POTENTIALLY LIFE-THREATENING
**RESPIRATORY**				
Bronchospasm (acute)	FEV1 or peak flow reduced to 70 – 80%	FEV1 or peak flow 50 – 69%	FEV1 or peak flow 25 – 49%	Cyanosis OR FEV1 or peak flow < 25% OR Intubation
Dyspnea or respiratory distress				
**Adult ≥ 14 years**	Dyspnea on exertion with no or minimal interference with usual social & functional activities	Dyspnea on exertion causing greater than minimal interference with usual social & functional activities	Dyspnea at rest causing inability to perform usual social & functional activities	Respiratory failure with ventilatory support indicated
**Pediatric < 14 years**	Wheezing OR minimal increase in respiratory rate for age	Nasal flaring OR Intercostal retractions OR Pulse oximetry 90 – 95%	Dyspnea at rest causing inability to perform usual social & functional activities OR Pulse oximetry < 90%	Respiratory failure with ventilatory support indicated
**MUSCULOSKELETAL**				
Arthralgia<br>See also Arthritis	Joint pain causing no or minimal interference with usual social & functional activities	Joint pain causing greater than minimal interference with usual social & functional activities	Joint pain causing inability to perform usual social & functional activities	Disabling joint pain causing inability to perform basic self-care functions
Arthritis<br>See also Arthralgia	Stiffness or joint swelling causing no or minimal interference with usual social & functional activities	Stiffness or joint swelling causing greater than minimal interference with usual social & functional activities	Stiffness or joint swelling causing inability to perform usual social & functional activities	Disabling joint stiffness or swelling causing inability to perform basic self-care functions
Bone Mineral Loss				
**Adult ≥ 21 years**	BMD t-score -2.5 to -1.0	BMD t-score < -2.5	Pathological fracture (including loss of vertebral height)	Pathologic fracture causing life-threatening consequences
**Pediatric < 21 years**	BMD z-score -2.5 to -1.0	BMD z-score < -2.5	Pathological fracture (including loss of vertebral height)	Pathologic fracture causing life-threatening consequences
Myalgia<br>(non-injection site)	Muscle pain causing no or minimal interference with usual social & functional activities	Muscle pain causing greater than minimal interference with usual social & functional activities	Muscle pain causing inability to perform usual social & functional activities	Disabling muscle pain causing inability to perform basic self-care functions

Basic Self-care Functions – Adult: Activities such as bathing, dressing, toileting, transfer/movement, continence, and feeding.

Basic Self-care Functions – Young Children: Activities that are age and culturally appropriate (e.g., feeding self with culturally appropriate eating implement).

Usual Social & Functional Activities – Adult: Adaptive tasks and desirable activities, such as going to work, shopping, cooking, use of transportation, pursuing a hobby, etc.

Usual Social & Functional Activities – Young Children: Activities that are age and culturally appropriate (e.g., social interactions, play activities, learning tasks, etc.).

28 Dec 04/Clarification Aug 09 Page 12 of 21 Version 1.0/Clarification 1

Approved v4.0 930056810 4.0

DIVISION OF AIDS TABLE FOR GRADING THE SEVERITY OF ADULT AND PEDIATRIC ADVERSE EVENTS
VERSION 1.0, DECEMBER, 2004; CLARIFICATION AUGUST 2009

PARAMETER	GRADE 1 MILD	GRADE 2 MODERATE	GRADE 3 SEVERE	GRADE 4 POTENTIALLY LIFE-THREATENING
Osteonecrosis	NA	Asymptomatic with radiographic findings AND No operative intervention indicated	Symptomatic bone pain with radiographic findings OR Operative intervention indicated	Disabling bone pain with radiographic findings causing inability to perform basic self-care functions
**GENITOURINARY**				
Cervicitis (symptoms) (For use in studies evaluating topical study agents) For other cervicitis see Infection: Infection (any other than HIV infection)	Symptoms causing no or minimal interference with usual social & functional activities	Symptoms causing greater than minimal interference with usual social & functional activities	Symptoms causing inability to perform usual social & functional activities	Symptoms causing inability to perform basic self-care functions
Cervicitis (clinical exam) (For use in studies evaluating topical study agents) For other cervicitis see Infection: Infection (any other than HIV infection)	Minimal cervical abnormalities on examination (erythema, mucopurulent discharge, or friability) OR Epithelial disruption < 25% of total surface	Moderate cervical abnormalities on examination (erythema, mucopurulent discharge, or friability) OR Epithelial disruption of 25 – 49% total surface	Severe cervical abnormalities on examination (erythema, mucopurulent discharge, or friability) OR Epithelial disruption 50 – 75% total surface	Epithelial disruption > 75% total surface
Inter-menstrual bleeding (IMB)	Spotting observed by participant OR Minimal blood observed during clinical or colposcopic examination	Inter-menstrual bleeding not greater in duration or amount than usual menstrual cycle	Inter-menstrual bleeding greater in duration or amount than usual menstrual cycle	Hemorrhage with life-threatening hypotension OR Operative intervention indicated
Urinary tract obstruction (e.g., stone)	NA	Signs or symptoms of urinary tract obstruction without hydronephrosis or renal dysfunction	Signs or symptoms of urinary tract obstruction with hydronephrosis or renal dysfunction	Obstruction causing life-threatening consequences

Basic Self-care Functions – Adult: Activities such as bathing, dressing, toileting, transfer/movement, continence, and feeding.

Basic Self-care Functions – Young Children: Activities that are age and culturally appropriate (e.g., feeding self with culturally appropriate eating implement).

Usual Social & Functional Activities – Adult: Adaptive tasks and desirable activities, such as going to work, shopping, cooking, use of transportation, pursuing a hobby, etc.

Usual Social & Functional Activities – Young Children: Activities that are age and culturally appropriate (e.g., social interactions, play activities, learning tasks, etc.).

Approved v4.0 930056810 4.0

DIVISION OF AIDS TABLE FOR GRADING THE SEVERITY OF ADULT AND PEDIATRIC ADVERSE EVENTS
VERSION 1.0, DECEMBER, 2004; CLARIFICATION AUGUST 2009

PARAMETER	GRADE 1 MILD	GRADE 2 MODERATE	GRADE 3 SEVERE	GRADE 4 POTENTIALLY LIFE-THREATENING
Vulvovaginitis (symptoms) (Use in studies evaluating topical study agents) For other vulvovaginitis see Infection: Infection (any other than HIV infection)	Symptoms causing no or minimal interference with usual social & functional activities	Symptoms causing greater than minimal interference with usual social & functional activities	Symptoms causing inability to perform usual social & functional activities	Symptoms causing inability to perform basic self-care functions
Vulvovaginitis (clinical exam) (Use in studies evaluating topical study agents) For other vulvovaginitis see Infection: Infection (any other than HIV infection)	Minimal vaginal abnormalities on examination OR Epithelial disruption < 25% of total surface	Moderate vaginal abnormalities on examination OR Epithelial disruption of 25 - 49% total surface	Severe vaginal abnormalities on examination OR Epithelial disruption 50 - 75% total surface	Vaginal perforation OR Epithelial disruption > 75% total surface
**OCULAR/VISUAL**				
Uveitis	Asymptomatic but detectable on exam	Symptomatic anterior uveitis OR Medical intervention indicated	Posterior or pan-uveitis OR Operative intervention indicated	Disabling visual loss in affected eye(s)
Visual changes (from baseline)	Visual changes causing no or minimal interference with usual social & functional activities	Visual changes causing greater than minimal interference with usual social & functional activities	Visual changes causing inability to perform usual social & functional activities	Disabling visual loss in affected eye(s)
**ENDOCRINE/METABOLIC**				
Abnormal fat accumulation (e.g., back of neck, breasts, abdomen)	Detectable by study participant (or by caregiver for young children and disabled adults)	Detectable on physical exam by health care provider	Disfiguring OR Obvious changes on casual visual inspection	NA
Diabetes mellitus	NA	New onset without need to initiate medication OR Modification of current medications to regain glucose control	New onset with initiation of medication indicated OR Diabetes uncontrolled despite treatment modification	Life-threatening consequences (e.g., ketoacidosis, hyperosmolar non-ketotic coma)

Basic Self-care Functions – Adult: Activities such as bathing, dressing, toileting, transfer/movement, continence, and feeding.

Basic Self-care Functions – Young Children: Activities that are age and culturally appropriate (e.g., feeding self with culturally appropriate eating implement).

Usual Social & Functional Activities – Adult: Adaptive tasks and desirable activities, such as going to work, shopping, cooking, use of transportation, pursuing a hobby, etc.

Usual Social & Functional Activities – Young Children: Activities that are age and culturally appropriate (e.g., social interactions, play activities, learning tasks, etc.).

Approved v4.0 930056810 4.0

DIVISION OF AIDS TABLE FOR GRADING THE SEVERITY OF ADULT AND PEDIATRIC ADVERSE EVENTS
VERSION 1.0, DECEMBER, 2004; CLARIFICATION AUGUST 2009

PARAMETER	GRADE 1 MILD	GRADE 2 MODERATE	GRADE 3 SEVERE	GRADE 4 POTENTIALLY LIFE-THREATENING
Gynecomastia	Detectable by study participant or caregiver (for young children and disabled adults)	Detectable on physical exam by health care provider	Disfiguring OR Obvious on casual visual inspection	NA
Hyperthyroidism	Asymptomatic	Symptomatic causing greater than minimal interference with usual social & functional activities OR Thyroid suppression therapy indicated	Symptoms causing inability to perform usual social & functional activities OR Uncontrolled despite treatment modification	Life-threatening consequences (e.g., thyroid storm)
Hypothyroidism	Asymptomatic	Symptomatic causing greater than minimal interference with usual social & functional activities OR Thyroid replacement therapy indicated	Symptoms causing inability to perform usual social & functional activities OR Uncontrolled despite treatment modification	Life-threatening consequences (e.g., myxedema coma)
Lipoatrophy (e.g., fat loss from the face, extremities, buttocks)	Detectable by study participant (or by caregiver for young children and disabled adults)	Detectable on physical exam by health care provider	Disfiguring OR Obvious on casual visual inspection	NA

Basic Self-care Functions – Adult: Activities such as bathing, dressing, toileting, transfer/movement, continence, and feeding.

Basic Self-care Functions – Young Children: Activities that are age and culturally appropriate (e.g., feeding self with culturally appropriate eating implement).

Usual Social & Functional Activities – Adult: Adaptive tasks and desirable activities, such as going to work, shopping, cooking, use of transportation, pursuing a hobby, etc.

Usual Social & Functional Activities – Young Children: Activities that are age and culturally appropriate (e.g., social interactions, play activities, learning tasks, etc.).

Approved v4.0 930056810 4.0

DIVISION OF AIDS TABLE FOR GRADING THE SEVERITY OF ADULT AND PEDIATRIC ADVERSE EVENTS
VERSION 1.0, DECEMBER, 2004; CLARIFICATION AUGUST 2009

LABORATORY				
**PARAMETER**	**GRADE 1 MILD**	**GRADE 2 MODERATE**	**GRADE 3 SEVERE**	**GRADE 4 POTENTIALLY LIFE-THREATENING**
**HEMATOLOGY**	***Standard International Units are listed in italics***			
Absolute CD4+ count **– Adult and Pediatric > 13 years** (HIV NEGATIVE ONLY)	300 – 400/mm^3<br>*300 – 400/μL*	200 – 299/mm^3<br>*200 – 299/μL*	100 – 199/mm^3<br>*100 – 199/μL*	< 100/mm^3<br>*< 100/μL*
Absolute lymphocyte count **– Adult and Pediatric > 13 years** (HIV NEGATIVE ONLY)	600 – 650/mm^3<br>*0.600 x 10^9 – 0.650 x 10^9/L*	500 – 599/mm^3<br>*0.500 x 10^9 – 0.599 x 10^9/L*	350 – 499/mm^3<br>*0.350 x 10^9 – 0.499 x 10^9/L*	< 350/mm^3<br>*< 0.350 x 10^9/L*
**Comment:** Values in children ≤ 13 years are not given for the two parameters above because the absolute counts are variable.				
Absolute neutrophil count (ANC)				
**Adult and Pediatric, > 7 days**	1,000 – 1,300/mm^3<br>*1.000 x 10^9 – 1.300 x 10^9/L*	750 – 999/mm^3<br>*0.750 x 10^9 – 0.999 x 10^9/L*	500 – 749/mm^3<br>*0.500 x 10^9 – 0.749 x 10^9/L*	< 500/mm^3<br>*< 0.500 x 10^9/L*
**Infant*†, 2 – ≤ 7 days**	1,250 – 1,500/mm^3<br>*1.250 x 10^9 – 1.500 x 10^9/L*	1,000 – 1,249/mm^3<br>*1.000 x 10^9 – 1.249 x 10^9/L*	750 – 999/mm^3<br>*0.750 x 10^9 – 0.999 x 10^9/L*	< 750/mm^3<br>*< 0.750 x 10^9/L*
**Infant*†, ≤1 day**	4,000 – 5,000/mm^3<br>*4.000 x 10^9 – 5.000 x 10^9/L*	3,000 – 3,999/mm^3<br>*3.000 x 10^9 – 3.999 x10^9/L*	1,500 – 2,999/mm^3<br>*1.500 x 10^9 – 2.999 x 10^9/L*	< 1,500/mm^3<br>*< 1.500 x 10^9/L*
**Comment:** Parameter changed from "Infant, < 1 day" to "Infant, ≤1 day"				
Fibrinogen, decreased	100 – 200 mg/dL<br>*1.00 – 2.00 g/L*<br>OR<br>0.75 – 0.99 x LLN	75 – 99 mg/dL<br>*0.75 – 0.99 g/L*<br>OR<br>0.50 – 0.74 x LLN	50 – 74 mg/dL<br>*0.50 – 0.74 g/L*<br>OR<br>0.25 – 0.49 x LLN	< 50 mg/dL<br>*< 0.50 g/L*<br>OR<br>< 0.25 x LLN<br>OR<br>Associated with gross bleeding

* Values are for term infants. Preterm infants should be assessed using local normal ranges.

† Use age and sex appropriate values (e.g., bilirubin).

28 Dec-04/Clarification Aug 09 Page 16 of 21 Version 1.0/Clarification 1

Approved v 4.0 930056810 4.0

DIVISION OF AIDS TABLE FOR GRADING THE SEVERITY OF ADULT AND PEDIATRIC ADVERSE EVENTS
VERSION 1.0, DECEMBER, 2004; CLARIFICATION AUGUST 2009

LABORATORY				
**PARAMETER**	**GRADE 1** **MILD**	**GRADE 2** **MODERATE**	**GRADE 3** **SEVERE**	**GRADE 4** POTENTIALLY LIFE-THREATENING
Hemoglobin (Hgb)				
**Comment:** The Hgb values in mmol/L have changed because the conversion factor used to convert g/dL to mmol/L has been changed from 0.155 to 0.6206 (the most commonly used conversion factor). For grading Hgb results obtained by an analytic method with a conversion factor other than 0.6206, the result must be converted to g/dL using the appropriate conversion factor for that lab.				
**Adult and Pediatric ≥ 57 days** (HIV POSITIVE ONLY)	8.5 – 10.0 g/dL<br>*5.24 – 6.23 mmol/L*	7.5 – 8.4 g/dL<br>*4.62–5.23 mmol/L*	6.50 – 7.4 g/dL<br>*4.03–4.61 mmol/L*	< 6.5 g/dL<br>*< 4.03 mmol/L*
**Adult and Pediatric ≥ 57 days** (HIV NEGATIVE ONLY)	10.0 – 10.9 g/dL<br>*6.18 – 6.79 mmol/L*<br>OR<br>Any decrease 2.5 – 3.4 g/dL<br>*1.58 – 2.13 mmol/L*	9.0 – 9.9 g/dL<br>*5.55 - 6.17 mmol/L*<br>OR<br>Any decrease 3.5 – 4.4 g/dL<br>*2.14 – 2.78 mmol/L*	7.0 – 8.9 g/dL<br>*4.34 - 5.54 mmol/L*<br>OR<br>Any decrease ≥ 4.5 g/dL<br>*> 2.79 mmol/L*	< 7.0 g/dL<br>*< 4.34 mmol/L*
**Comment:** The decrease is a decrease from baseline				
**Infant*†, 36 – 56 days** (HIV POSITIVE OR NEGATIVE)	8.5 – 9.4 g/dL<br>*5.24 – 5.86 mmol/L*	7.0 – 8.4 g/dL<br>*4.31 – 5.23 mmol/L*	6.0 – 6.9 g/dL<br>*3.72 – 4.30 mmol/L*	< 6.00 g/dL<br>*< 3.72 mmol/L*
**Infant*†, 22 – 35 days** (HIV POSITIVE OR NEGATIVE)	9.5 – 10.5 g/dL<br>*5.87 - 6.54 mmol/L*	8.0 – 9.4 g/dL<br>*4.93 – 5.86 mmol/L*	7.0 – 7.9 g/dL<br>*4.34 – 4.92 mmol/L*	< 7.00 g/dL<br>*< 4.34 mmol/L*
**Infant*†, ≤ 21 days** (HIV POSITIVE OR NEGATIVE)	12.0 – 13.0 g/dL<br>*7.42 – 8.09 mmol/L*	10.0 – 11.9 g/dL<br>*6.18 – 7.41 mmol/L*	9.0 – 9.9 g/dL<br>*5.59- 6.17 mmol/L*	< 9.0 g/dL<br>*< 5.59 mmol/L*
**Correction:** Parameter changed from "Infant < 21 days" to "Infant ≤ 21 days"				
International Normalized Ratio of prothrombin time (INR)	1.1 – 1.5 x ULN	1.6 – 2.0 x ULN	2.1 – 3.0 x ULN	> 3.0 x ULN
Methemoglobin	5.0 – 10.0%	10.1 – 15.0%	15.1 – 20.0%	> 20.0%
Prothrombin Time (PT)	1.1 – 1.25 x ULN	1.26 – 1.50 x ULN	1.51 – 3.00 x ULN	> 3.00 x ULN
Partial Thromboplastin Time (PTT)	1.1 – 1.66 x ULN	1.67 – 2.33 x ULN	2.34 – 3.00 x ULN	> 3.00 x ULN
Platelets, decreased	100,000 – 124,999/mm^3<br>*100.000 x 10^9 – 124.999 x 10^9/L*	50,000 – 99,999/mm^3<br>*50.000 x 10^9 – 99.999 x 10^9/L*	25,000 – 49,999/mm^3<br>*25.000 x 10^9 – 49.999 x 10^9/L*	< 25,000/mm^3<br>*< 25.000 x 10^9/L*
WBC, decreased	2,000 – 2,500/mm^3<br>*2.000 x 10^9 – 2.500 x 10^9/L*	1,500 – 1,999/mm^3<br>*1.500 x 10^9 – 1.999 x 10^9/L*	1,000 – 1,499/mm^3<br>*1.000 x 10^9 – 1.499 x 10^9/L*	< 1,000/mm^3<br>*< 1.000 x 10^9/L*

* Values are for term infants. Preterm infants should be assessed using local normal ranges.

† Use age and sex appropriate values (e.g., bilirubin).

28 Dec-04/Clarification Aug 09 Page 17 of 21 Version 1.0/Clarification 1

Approved v4.0 930056810 4.0

DIVISION OF AIDS TABLE FOR GRADING THE SEVERITY OF ADULT AND PEDIATRIC ADVERSE EVENTS
VERSION 1.0, DECEMBER, 2004; CLARIFICATION AUGUST 2009

LABORATORY				
**PARAMETER**	**GRADE 1 MILD**	**GRADE 2 MODERATE**	**GRADE 3 SEVERE**	**GRADE 4 POTENTIALLY LIFE-THREATENING**
**CHEMISTRIES**	***Standard International Units are listed in italics***			
Acidosis	NA	pH < normal, but ≥ 7.3	pH < 7.3 without life-threatening consequences	pH < 7.3 with life-threatening consequences
Albumin, serum, low	3.0 g/dL – < LLN<br>*30 g/L – < LLN*	2.0 – 2.9 g/dL<br>*20 – 29 g/L*	< 2.0 g/dL<br>*< 20 g/L*	NA
Alkaline Phosphatase	1.25 – 2.5 x ULN[†]	2.6 – 5.0 x ULN[†]	5.1 – 10.0 x ULN[†]	> 10.0 x ULN[†]
Alkalosis	NA	pH > normal, but ≤ 7.5	pH > 7.5 without life-threatening consequences	pH > 7.5 with life-threatening consequences
ALT (SGPT)	1.25 – 2.5 x ULN	2.6 – 5.0 x ULN	5.1 – 10.0 x ULN	> 10.0 x ULN
AST (SGOT)	1.25 – 2.5 x ULN	2.6 – 5.0 x ULN	5.1 – 10.0 x ULN	> 10.0 x ULN
Bicarbonate, serum, low	16.0 mEq/L – < LLN<br>*16.0 mmol/L – < LLN*	11.0 – 15.9 mEq/L<br>*11.0 – 15.9 mmol/L*	8.0 – 10.9 mEq/L<br>*8.0 – 10.9 mmol/L*	< 8.0 mEq/L<br>*< 8.0 mmol/L*
**Comment:** Some laboratories will report this value as Bicarbonate (HCO_3) and others as Total Carbon Dioxide (CO_2). These are the same tests; values should be graded according to the ranges for Bicarbonate as listed above.				
Bilirubin (Total)				
**Adult and Pediatric > 14 days**	1.1 – 1.5 x ULN	1.6 – 2.5 x ULN	2.6 – 5.0 x ULN	> 5.0 x ULN
**Infant[*,†], ≤ 14 days** (non-hemolytic)	NA	20.0 – 25.0 mg/dL<br>*342 – 428 μmol/L*	25.1 – 30.0 mg/dL<br>*429 – 513 μmol/L*	> 30.0 mg/dL<br>*> 513.0 μmol/L*
**Infant[*,†], ≤ 14 days** (hemolytic)	NA	NA	20.0 – 25.0 mg/dL<br>*342 – 428 μmol/L*	> 25.0 mg/dL<br>*> 428 μmol/L*
Calcium, serum, high				
**Adult and Pediatric ≥ 7 days**	10.6 – 11.5 mg/dL<br>*2.65 – 2.88 mmol/L*	11.6 – 12.5 mg/dL<br>*2.89 – 3.13 mmol/L*	12.6 – 13.5 mg/dL<br>*3.14 – 3.38 mmol/L*	> 13.5 mg/dL<br>*> 3.38 mmol/L*
**Infant[*,†], < 7 days**	11.5 – 12.4 mg/dL<br>*2.88 – 3.10 mmol/L*	12.5 – 12.9 mg/dL<br>*3.11 – 3.23 mmol/L*	13.0 – 13.5 mg/dL<br>*3.245 – 3.38 mmol/L*	> 13.5 mg/dL<br>*> 3.38 mmol/L*
Calcium, serum, low				
**Adult and Pediatric ≥ 7 days**	7.8 – 8.4 mg/dL<br>*1.95 – 2.10 mmol/L*	7.0 – 7.7 mg/dL<br>*1.75 – 1.94 mmol/L*	6.1 – 6.9 mg/dL<br>*1.53 – 1.74 mmol/L*	< 6.1 mg/dL<br>*< 1.53 mmol/L*
**Infant[*,†], < 7 days**	6.5 – 7.5 mg/dL<br>*1.63 – 1.88 mmol/L*	6.0 – 6.4 mg/dL<br>*1.50 – 1.62 mmol/L*	5.50 – 5.90 mg/dL<br>*1.38 – 1.51 mmol/L*	< 5.50 mg/dL<br>*< 1.38 mmol/L*
**Comment:** Do not adjust Calcium, serum, low or Calcium, serum, high for albumin				

[*] Values are for term infants. Preterm infants should be assessed using local normal ranges.

[†] Use age and sex appropriate values (e.g., bilirubin).

Approved v4.0 930056810 4.0

DIVISION OF AIDS TABLE FOR GRADING THE SEVERITY OF ADULT AND PEDIATRIC ADVERSE EVENTS
VERSION 1.0, DECEMBER, 2004; CLARIFICATION AUGUST 2009

LABORATORY				
**PARAMETER**	**GRADE 1 MILD**	**GRADE 2 MODERATE**	**GRADE 3 SEVERE**	**GRADE 4 POTENTIALLY LIFE-THREATENING**
Cardiac troponin I (cTnI)	NA	NA	NA	Levels consistent with myocardial infarction or unstable angina as defined by the manufacturer
Cardiac troponin T (cTnT)	NA	NA	NA	≥ 0.20 ng/mL OR Levels consistent with myocardial infarction or unstable angina as defined by the manufacturer
Cholesterol (fasting)				
**Adult ≥ 18 years**	200 – 239 mg/dL *5.18 – 6.19 mmol/L*	240 – 300 mg/dL *6.20 – 7.77 mmol/L*	> 300 mg/dL *> 7.77 mmol/L*	NA
**Pediatric < 18 years**	170 – 199 mg/dL *4.40 – 5.15 mmol/L*	200 – 300 mg/dL *5.16 – 7.77 mmol/L*	> 300 mg/dL *> 7.77 mmol/L*	NA
Creatine Kinase	3.0 – 5.9 x ULN†	6.0 – 9.9 x ULN†	10.0 – 19.9 x ULN†	≥ 20.0 x ULN†
Creatinine	1.1 – 1.3 x ULN†	1.4 – 1.8 x ULN†	1.9 – 3.4 x ULN†	≥ 3.5 x ULN†

LABORATORY				
**PARAMETER**	**GRADE 1 MILD**	**GRADE 2 MODERATE**	**GRADE 3 SEVERE**	**GRADE 4 POTENTIALLY LIFE-THREATENING**
Glucose, serum, high				
Nonfasting	116 – 160 mg/dL *6.44 – 8.88 mmol/L*	161 – 250 mg/dL *8.89 – 13.88 mmol/L*	251 – 500 mg/dL *13.89 – 27.75 mmol/L*	> 500 mg/dL *> 27.75 mmol/L*
Fasting	110 – 125 mg/dL *6.11 – 6.94 mmol/L*	126 – 250 mg/dL *6.95 – 13.88 mmol/L*	251 – 500 mg/dL *13.89 – 27.75 mmol/L*	> 500 mg/dL *> 27.75 mmol/L*
Glucose, serum, low				
**Adult and Pediatric ≥ 1 month**	55 – 64 mg/dL *3.05 – 3.55 mmol/L*	40 – 54 mg/dL *2.22 – 3.06 mmol/L*	30 – 39 mg/dL *1.67 – 2.23 mmol/L*	< 30 mg/dL *< 1.67 mmol/L*
**Infant*†, < 1 month**	50 – 54 mg/dL *2.78 – 3.00 mmol/L*	40 – 49 mg/dL *2.22 – 2.77 mmol/L*	30 – 39 mg/dL *1.67 – 2.21 mmol/L*	< 30 mg/dL *< 1.67 mmol/L*
Lactate	ULN - < 2.0 x ULN without acidosis	≥ 2.0 x ULN without acidosis	Increased lactate with pH < 7.3 without life-threatening consequences	Increased lactate with pH < 7.3 with life-threatening consequences

* Values are for term infants. Preterm infants should be assessed using local normal ranges.

† Use age and sex appropriate values (e.g., bilirubin).

Approved v4.0 930056810 4.0

DIVISION OF AIDS TABLE FOR GRADING THE SEVERITY OF ADULT AND PEDIATRIC ADVERSE EVENTS
VERSION 1.0, DECEMBER, 2004; CLARIFICATION AUGUST 2009

**Comment:** Added ULN to Grade 1 parameter				
LDL cholesterol (fasting)				
**Adult ≥ 18 years**	130 – 159 mg/dL<br>*3.37 – 4.12 mmol/L*	160 – 190 mg/dL<br>*4.13 – 4.90 mmol/L*	≥ 190 mg/dL<br>*≥ 4.91 mmol/L*	NA
**Pediatric > 2 - < 18 years**	110 – 129 mg/dL<br>*2.85 – 3.34 mmol/L*	130 – 189 mg/dL<br>*3.35 – 4.90 mmol/L*	≥ 190 mg/dL<br>*≥ 4.91 mmol/L*	NA
Lipase	1.1 – 1.5 x ULN	1.6 – 3.0 x ULN	3.1 – 5.0 x ULN	> 5.0 x ULN
Magnesium, serum, low	1.2 – 1.4 mEq/L<br>*0.60 – 0.70 mmol/L*	0.9 – 1.1 mEq/L<br>*0.45 – 0.59 mmol/L*	0.6 – 0.8 mEq/L<br>*0.30 – 0.44 mmol/L*	< 0.60 mEq/L<br>*< 0.30 mmol/L*
Pancreatic amylase	1.1 – 1.5 x ULN	1.6 – 2.0 x ULN	2.1 – 5.0 x ULN	> 5.0 x ULN
Phosphate, serum, low				
**Adult and Pediatric > 14 years**	2.5 mg/dL – < LLN<br>*0.81 mmol/L – < LLN*	2.0 – 2.4 mg/dL<br>*0.65 – 0.80 mmol/L*	1.0 – 1.9 mg/dL<br>*0.32 – 0.64 mmol/L*	< 1.00 mg/dL<br>*< 0.32 mmol/L*
**Pediatric 1 year – 14 years**	3.0 – 3.5 mg/dL<br>*0.97 – 1.13 mmol/L*	2.5 – 2.9 mg/dL<br>*0.81 – 0.96 mmol/L*	1.5 – 2.4 mg/dL<br>*0.48 – 0.80 mmol/L*	< 1.50 mg/dL<br>*< 0.48 mmol/L*
**Pediatric < 1 year**	3.5 – 4.5 mg/dL<br>*1.13 – 1.45 mmol/L*	2.5 – 3.4 mg/dL<br>*0.81 – 1.12 mmol/L*	1.5 – 2.4 mg/dL<br>*0.48 – 0.80 mmol/L*	< 1.50 mg/dL<br>*< 0.48 mmol/L*
Potassium, serum, high	5.6 – 6.0 mEq/L<br>*5.6 – 6.0 mmol/L*	6.1 – 6.5 mEq/L<br>*6.1 – 6.5 mmol/L*	6.6 – 7.0 mEq/L<br>*6.6 – 7.0 mmol/L*	> 7.0 mEq/L<br>*> 7.0 mmol/L*
Potassium, serum, low	3.0 – 3.4 mEq/L<br>*3.0 – 3.4 mmol/L*	2.5 – 2.9 mEq/L<br>*2.5 – 2.9 mmol/L*	2.0 – 2.4 mEq/L<br>*2.0 – 2.4 mmol/L*	< 2.0 mEq/L<br>*< 2.0 mmol/L*
Sodium, serum, high	146 – 150 mEq/L<br>*146 – 150 mmol/L*	151 – 154 mEq/L<br>*151 – 154 mmol/L*	155 – 159 mEq/L<br>*155 – 159 mmol/L*	≥ 160 mEq/L<br>*≥ 160 mmol/L*
Sodium, serum, low	130 – 135 mEq/L<br>*130 – 135 mmol/L*	125 – 129 mEq/L<br>*125 – 129 mmol/L*	121 – 124 mEq/L<br>*121 – 124 mmol/L*	≤ 120 mEq/L<br>*≤ 120 mmol/L*
Triglycerides (fasting)	NA	500 – 750 mg/dL<br>*5.65 – 8.48 mmol/L*	751 – 1,200 mg/dL<br>*8.49 – 13.56 mmol/L*	> 1,200 mg/dL<br>*> 13.56 mmol/L*

* Values are for term infants. Preterm infants should be assessed using local normal ranges.

† Use age and sex appropriate values (e.g., bilirubin).

Approved v4.0 930056810 4.0

DIVISION OF AIDS TABLE FOR GRADING THE SEVERITY OF ADULT AND PEDIATRIC ADVERSE EVENTS
VERSION 1.0, DECEMBER, 2004; CLARIFICATION AUGUST 2009

LABORATORY				
**PARAMETER**	**GRADE 1 MILD**	**GRADE 2 MODERATE**	**GRADE 3 SEVERE**	**GRADE 4 POTENTIALLY LIFE-THREATENING**
Uric acid	7.5 – 10.0 mg/dL<br>*0.45 – 0.59 mmol/L*	10.1 – 12.0 mg/dL<br>*0.60 – 0.71 mmol/L*	12.1 – 15.0 mg/dL<br>*0.72 – 0.89 mmol/L*	> 15.0 mg/dL<br>*> 0.89 mmol/L*
**URINALYSIS**	***Standard International Units are listed in italics***			
Hematuria (microscopic)	6 – 10 RBC/HPF	> 10 RBC/HPF	Gross, with or without clots OR with RBC casts	Transfusion indicated
Proteinuria, random collection	1 +	2 – 3 +	4 +	NA
Proteinuria, 24 hour collection				
**Adult and Pediatric ≥ 10 years**	200 – 999 mg/24 h<br>*0.200 – 0.999 g/d*	1,000 – 1,999 mg/24 h<br>*1.000 – 1.999 g/d*	2,000 – 3,500 mg/24 h<br>*2.000 – 3.500 g/d*	> 3,500 mg/24 h<br>*> 3.500 g/d*
**Pediatric > 3 mo - < 10 years**	201 – 499 mg/m^2/24 h<br>*0.201 – 0.499 g/d*	500 – 799 mg/m^2/24 h<br>*0.500 – 0.799 g/d*	800 – 1,000 mg/m^2/24 h<br>*0.800 – 1.000 g/d*	> 1,000 mg/ m^2/24 h<br>*> 1.000 g/d*

* Values are for term infants. Preterm infants should be assessed using local normal ranges.

† Use age and sex appropriate values (e.g., bilirubin).

Approved v4.0 930056810 4.0
