# Supplementary material for: A Randomized Study of Peginterferon Lambda-1a Compared to Peginterferon Alfa-2a in Combination with Ribavirin and Telaprevir in Patients with Genotype-1 Chronic Hepatitis C
Source: PLoS One. 2016 Oct 17;11(10):e0164563. doi: 10.1371/journal.pone.0164563 (PMC5066958; doi:10.1371/journal.pone.0164563)
Supplement: S1 IRBs — (DOC) [file pone.0164563.s003.doc]

**List of Independent Ethics Committees and/or Institutional Review Boards for Study AI452-020**

|  | **Site #** | **Principal Investigator** | **Name and Address of IRB/IEC and IRB/IEC Chairpersons** |
| --- | --- | --- | --- |
|  | AI452-020-0001 | **Younes, Ziad H, MD** | Schulman Associates Irb  4445 Lake Forest Drive  Cincinnati, OH 45242  USA  Chairpersons:  Nelson, Sharon Lynn, MSN, RN, CNS |
|  | AI452-020-0002 | **Tatum, Harvey A, MD** | Schulman Associates Irb  4445 Lake Forest Drive  Cincinnati, OH 45242  USA  Chairpersons:  Nelson, Sharon Lynn, MSN, RN, CNS |
|  | AI452-020-0003 | **Sheikh, Aasim M, MD** | Schulman Associates Irb  4445 Lake Forest Drive  Cincinnati, OH 45242  USA  Chairpersons:  Nelson, Sharon Lynn, MSN, RN, CNS |
|  | AI452-020-0004 | **Morris, David W, MD**  **Morris, David W, DO** | Schulman Associates Irb  4445 Lake Forest Drive  Cincinnati, OH 45242  USA  Chairpersons:  Nelson, Sharon Lynn, MSN, RN, CNS |
|  | AI452-020-0005 | **Marbury, Thomas C, MD** | Schulman Associates Irb  4445 Lake Forest Dr  Ste 300  Cincinnati, OH 45242  USA  Chairpersons:  Nelson, Sharon Lynn, MSN, RN, CNS |
|  | AI452-020-0006 | **Demicco, Michael P, MD, FACG, ABAM** | Schulman Associates Irb  4445 Lake Forest Drive  Cincinnati, OH 45242  USA  Chairpersons:  Nelson, Sharon Lynn, MSN, RN, CNS |
|  | AI452-020-0008 | **Rustgi, Vinod K, MD** | Schulman Associates Irb  4445 Lake Forest Drive  Cincinnati, OH 45242  USA  Chairpersons:  Nelson, Sharon Lynn, MSN, RN, CNS |
|  | AI452-020-0012* | **Delwaide, Jean, MBBS** | Chu Du Sart Tilman  Comite D'ethique Hospitalo-facultaire De Liege  Centre Hospitalier Universitaire Du Sart Tilman,b35  Domaine Universitaire Du Sart Tilman  Liege, 4000  Belgium  Commissie Medische Ethiek- Uz Gasthuisberg  Toetsingscommissie E330  Herestraat 49  Leuven, 3000  Belgium  Chairpersons:  Van Den Bogaert, Walter, MD  Seutin, Vincent, MD |
|  | AI452-020-0013* | **Nevens, Frederik, PhD** | Commissie Medische Ethiek- Uz Gasthuisberg  Toetsingscommissie E330  Herestraat 49  Leuven, 3000  Belgium  Chairpersons:  Van Den Bogaert, Walter, MD |
|  | AI452-020-0014* | **Grinsztejn, Beatriz, MD** | Comite De Etica Em Pesquisa Do Ipec  Av. Brasil, 4365  Manguinhos  Rio De Janeiro, Rio De Janeiro 21040-000  Brazil  Comissao Nacional De Etica Em Pesquisa - Conep  Sepn 510 Norte, Bl. A 1º Subsolo  Ed Ex-inan-unid. Ii-min. Saude  Brasilia, Distrito Federal 70750-521  Brazil  Comite De Etica Em Pesquisa Do Ipec  Av. Brasil, 4365  Manguinhos  Rio De Janeiro, Rio De Janeiro 21040-900  Brazil (Previous)  Chairpersons:  de Almeida Venancio, Jorge Alves, MD  Camillo Coura, Lea, MD |
|  | AI452-020-0015* | **Cheinquer, Hugo, MD** | Comissao Nacional De Etica Em Pesquisa - Conep  Sepn 510 Norte, Bl. A 1º Subsolo  Ed Ex-inan-unid. Ii-min. Saude  Brasilia, Distrito Federal 70750-521  Brazil  Cep Do Hospital Das Clinicas De Porto Alegre  Rua Ramiro Barcelos, 2350 - 2o Andar Sala 2227  Porto Alegre, Rio Grande Do Sul 90035-903  Brazil  Chairpersons:  Goldim, José Roberto, MD  Lopes, Anibal Gil  de Almeida Venancio, Jorge Alves, MD |
|  | AI452-020-0016* | **Parise, Edison Roberto, MD** | Comissao Nacional De Etica Em Pesquisa - Conep  Sepn 510 Norte, Bl. A 1º Subsolo  Ed Ex-inan-unid. Ii-min. Saude  Brasilia, Distrito Federal 70750-521  Brazil  Unifesp-Epm  Rua Botucatu, 572 - 1o. Andar  Vila Clementino  Sao Paulo, Sao Paulo 04023-062  Brazil  Chairpersons:  Medina Pestana, Jose Osmar, MD  de Almeida Venancio, Jorge Alves, MD |
|  | AI452-020-0017* | **De Andrade Neto, Jose Luiz, MD** | Comissao Nacional De Etica Em Pesquisa - Conep  Sepn 510 Norte, Bl. A 1º Subsolo  Ed Ex-inan-unid. Ii-min. Saude  Brasilia, Distrito Federal 70750-521  Brazil  Chairpersons:  Tambara Filho, Renato, MD  de Almeida Venancio, Jorge Alves, MD |
|  | AI452-020-0018* | **Ferreira Filho, Raymundo Parana, MD** | Comissao Nacional De Etica Em Pesquisa - Conep  Sepn 510 Norte, Bl. A 1º Subsolo  Ed Ex-inan-unid. Ii-min. Saude  Brasilia, Distrito Federal 70750-521  Brazil  Cep Com Hos Prof Edgard Santos  Rua Augusto Vianna S/n  1 Andar  Salvador, Bahia 40110-060  Brazil  Chairpersons:  de Almeida Venancio, Jorge Alves, MD |
|  | AI452-020-0020* | **Silva, Giovanni Faria, MD, PhD**  **Silva, Giovanni Faria, MD** | Comissao Nacional De Etica Em Pesquisa - Conep  Sepn 510 Norte, Bl. A 1º Subsolo  Ed Ex-inan-unid. Ii-min. Saude  Brasilia, Distrito Federal 70750-521  Brazil  Cep Da Faculdade De Medicina De Botucatu  Rua Rubiao Junior, S/n  Botucatu, Sao Paulo 18618-000  Brazil  Cep Da Faculdade De Medicina De Botucatu  Rua Rubiao Junior, S/n  Botucatu, Sao Paulo 18618-970  Brazil(Previous)  Chairpersons:  Sardenberg, Trajano, MD  de Almeida Venancio, Jorge Alves, MD |
|  | AI452-020-0021* | **Horsmans, Yves, MD, PhD** | Commissie Medische Ethiek  Commission D'ethique Biomedicale Hospitalo-facultaire  Avenue Hippocrate 55.14  Tour Harvey, Niveau 0  Bruxelles, 1200  Belgium  Commissie Medische Ethiek- Uz Gasthuisberg  Toetsingscommissie E330  Herestraat 49  Leuven, 3000  Belgium  Chairpersons:  Maloteaux, Jean-Marie, MD  Van Den Bogaert, Walter, MD |
|  | AI452-020-0022* | **Agarwal, Kosh, MBBS, MD** | South West Rec Centre  South Central - Oxford A  Level 3, Block B  Whitefriars  Lewins Mead  Bristol, Avon BS1 2NT  United Kingdom  Chairpersons:  Sheridan, John |
|  | AI452-020-0023* | **Brown, Ashley, MBChB** | Berkshire Research Ethics Committee  South West Rec Centre  Level 3, Block B  Whitefriars  Lewins Mead  Bristol, Avon BS1 2NT  United Kingdom  Chairpersons:  Sheridan, John |
|  | AI452-020-0024* | **Mutimer, David, MBBS, FRACP** | Berkshire Research Ethics Committee  South West Rec Centre  Level 3, Block B  Whitefriars  Lewins Mead  Bristol, Avon BS1 2NT  United Kingdom  Chairpersons:  Sheridan, John |
|  | AI452-020-0026* | **Francque, Sven, MD, PhD**  **Francque, Sven, MD (Previous)** | Universitair Ziekenhuis Antwerpen  Comite Voor Medische Ethiek  Wilrijkstraat 10  Edegem, 2650  Belgium  Commissie Medische Ethiek- Uz Gasthuisberg  Toetsingscommissie E330  Herestraat 49  Leuven, 3000  Belgium  Chairpersons:  Van Den Bogaert, Walter, MD  Cras, Patrick, MD |
|  | AI452-020-0027* | **Abouda, George, MB, BCh, MD** | Berkshire Research Ethics Committee  South West Rec Centre  Level 3, Block B  Whitefriars  Lewins Mead  Bristol, Avon BS1 2NT  United Kingdom  Chairpersons:  Sheridan, John |
|  | AI452-020-0028* | **Sperl, Jan, MD, PhD**  **Sperl, Jan, MD (Previous)** | Thomayerova nemocnice  Videnska 800  Eticka komise IKEM a TN  Praha 4, 140 59  Czech Republic  Chairpersons:  Stanek, Vladimir, MD, PhD |
|  | AI452-020-0029* | **Urbanek, Petr, MD** | Thomayerova nemocnice  Videnska 800  Eticka komise IKEM a TN  Praha 4, 140 59  Czech Republic  Chairpersons:  Stanek, Vladimir, MD, PhD |
|  | AI452-020-0030* | **Plisek, Stanislav, MD** | Thomayerova nemocnice  Videnska 800  Eticka komise IKEM a TN  Praha 4, 140 59  Czech Republic  Eticka komise Fakultni nemocnice Hradec Kralove  Sokolska 581  Hradec Kralove, 500 05  Czech Republic  Ethics Committee Of Ikem And Ftnsp  Thomayer University Hospital  Videnska 800  Prague 4, 140 59  Czech Republic (Previous)  Chairpersons:  Vortel, Jiri, MD  Stanek, Vladimir, MD, PhD |
|  | AI452-020-0031* | **Ogurtsov, Pavel, MD, PhD** | Center for Liver Research of Russian University of Peoples Friendship  Mikluho-Maklaya str., 6  Moscow, 117198  Russian Federation  Ethic Council Under Moh Of Russian Federation  3, Rahmanovskiy Per.  Gsp-4  Moscow, 127994  Russian Federation  Russian People'S Friendship University  6, Mikloukho-maklaja Str.,  Department Faculty Therapy  Moscow, 117198  Russian Federation  Chairpersons:  Torshin, Vladimir Ivanovich  Chuchalin, Alexander G |
|  | AI452-020-0032* | **Kizhlo, Svetlana, DR**  **Kizhlo, Svetlana, MD (Previous)** | Ethic Council Under Moh Of Russian Federation  3, Rahmanovskiy Per.  Gsp-4  Moscow, 127994  Russian Federation  Center Of Profilactic Aids And Infectious Diseasas  12a, Bumazhnaya Street  St.petersburg, 190020  Russian Federation  St Petersburg AIDS Center  12a, Bumazhnaya Street  St.petersburg, 190020  Russian Federation (Previous)  Chairpersons:  Kuptsov, Dmitry, MD, PhD  Chuchalin, Alexander G |
|  | AI452-020-0033* | **Nikitin, Igor, MD** | Central Clinical Hospital Russian Academy Of Science  Ethic Committe Of Central Clinical Hospital Of Russian Academy Of Science  10, Bld.1 Fotievoi St.  Moscow, 117333  Russian Federation  Ethic Council Under Moh Of Russian Federation  3, Rahmanovskiy Per.  Gsp-4  Moscow, 127994  Russian Federation  Central Clinical Hospital Of Russian Science Academy  Ethic Committe Of Central Clinical Hospital Of Russian Academy Of Science  10, Bld.1 Fotievoi St.  Moscow, 117333  Russian Federation (Previous)  Chairpersons:  Chuchalin, Alexander G  Alekhin, Alexander, MD, PhD |
|  | AI452-020-0034* | **Isakov, Vasily A, MD, PhD** | Institution Of Nutrition Of Russian Academy Of Med Science  21 Kashirskoe Shosse  Moscow, 115446  Russian Federation  Ethic Council Under Moh Of Russian Federation  3, Rahmanovskiy Per.  Gsp-4  Moscow, 127994  Russian Federation  Chairpersons:  Chuchalin, Alexander G  Gapparova, Kamilat, MD, PhD |
|  | AI452-020-0035* | **Pokrovsky, VV** | Central Research Institute Of Epidemiology, Moh  3a, Novogireevskaya Str  Moscow, 111123  Russian Federation  Ethic Council Under Moh Of Russian Federation  3, Rahmanovskiy Per.  Gsp-4  Moscow, 127994  Russian Federation  Central Research Institute Of Epidemiology, Moh  3a, Novogireevskaya Str  Moscow, 111123  Russian Federation(Previous)  Chairpersons:  Shabalina, Svetlana, MD, PhD  Chuchalin, Alexander G |
|  | AI452-020-0036* | **Chulanov, Vladimir, MD, PhD** | Central Research Institute Of Epidemiology  Local Irb/ec  3a, Novogireevskaya Str.  Moscow, 111123  Russian Federation  Ethic Council Under Moh Of Russian Federation  3, Rahmanovskiy Per.  Gsp-4  Moscow, 127994  Russian Federation  Chairpersons:  Chuchalin, Alexander G.  Shabalina, Svetlana, MD, PhD |
|  | AI452-020-0037* | **Burnevich, Eduard Z, MD, PhD** | City Clinical Hospital #24  Pistsovaya str,.build10  Moscow, 127015  Russian Federation  Ethic Council Under Moh Of Russian Federation  3, Rahmanovskiy Per.  Gsp-4  Moscow, 127994  Russian Federation  Chairpersons:  Naumov, OL  Chuchalin, Alexander G |
|  | AI452-020-0039* | **Bogomolov, Pavel, MD** | Ethic Council Under Moh Of Russian Federation  3, Rahmanovskiy Per.  Gsp-4  Moscow, 127994  Russian Federation  Local Ethic Committee of LLC Clinical Hospital of Tsentrosoyuz  57, Gilyarovskogo str  Moscow, 107996  Russian Federation  Chairpersons:  Gukasyan, Samvel, MD  Chuchalin, Alexander G |
|  | AI452-020-0040* | **Geyvandova, Natalia, MD, PhD** | Ethic Council Under Moh Of Russian Federation  3, Rahmanovskiy Per.  Gsp-4  Moscow, 127994  Russian Federation  Stavropol Medical Academy  310, Mira St.  Stavropol, 355017  Russian Federation  Stavropol State Medical University  208, Lermontova Street  Stavropol, 355002  Russian Federation  Chairpersons:  Arushanyan, Eduard, MD, PhD  Chuchalin, Alexander G |
|  | AI452-020-0041* | **Flisiak, Robert, MD, PhD** | Komisja Bioetyczna Akademii Medycznej W Bialymstoku  Ul. Kilinskiego 1  Bialystok, 15-089  Poland |
|  | AI452-020-0042* | **Janczewska, Ewa, MD, PhD** | Komisja Bioetyczna Akademii Medycznej W Bialymstoku  Ul. Kilinskiego 1  Bialystok, 15-089  Poland |
|  | AI452-020-0043* | **Kryczka, Wieslaw, MD** | Komisja Bioetyczna Akademii Medycznej W Bialymstoku  Ul. Kilinskiego 1  Bialystok, 15-089  Poland |
|  | AI452-020-0044* | **Olszok, Iwona, MD** | Komisja Bioetyczna Akademii Medycznej W Bialymstoku  Ul. Kilinskiego 1  Bialystok, 15-089  Poland |
|  | AI452-020-0045* | **Inglot, Malgorzata**  **Inglot, Malgorzata, MD, PhD**  **Knysz, Brygida, MD** | Komisja Bioetyczna Akademii Medycznej W Bialymstoku  Ul. Kilinskiego 1  Bialystok, 15-089  Poland |
|  | AI452-020-0047* | **Bellot, Pablo, MD**  **Such, Jose, MD (Previous)** | Hospital General Universitario De Alicante  Avda. Pintor Baeza, 12  Ed. Gris Planta 3  Alicante, 03010  Spain  Chairpersons:  Horga De La Parte, Jose Francisco |
|  | AI452-020-0048* | **Romero, Manuel, MD** | Hospital General Universitario De Alicante  Avda. Pintor Baeza, 12  Ed. Gris Planta 3  Alicante, 03010  Spain  Hospital Universitario De Valme  Secretaria Del Ceic (reg.gral)  Ctra. Cadiz, S/n  Sevilla, 41014  Spain (Previous)  Chairpersons:  Horga De La Parte, Jose Francisco |
|  | AI452-020-0049* | **Molina, Esther, MD** | Hospital General Universitario De Alicante  Avda. Pintor Baeza, 12  Ed. Gris Planta 3  Alicante, 03010  Spain  Chairpersons:  Horga De La Parte, Jose Francisco |
|  | AI452-020-0050* | **Lopez Calvo, Soledad** | Hospital General Universitario De Alicante  Avda. Pintor Baeza, 12  Ed. Gris Planta 3  Alicante, 03010  Spain  Comite Etico De Investigacion Clinica De Galicia (Sergas)  Edificio Admin. San Lazaro,s/n  Santiago De Compostela  A Coruna, 15703  Spain(Previous)  Chairpersons:  Horga De La Parte, Jose Francisco |
|  | AI452-020-0051* | **Pascasio, Juan Manuel** | Hospital General Universitario De Alicante  Avda. Pintor Baeza, 12  Ed. Gris Planta 3  Alicante, 03010  Spain  Chairpersons:  Horga De La Parte, Jose Francisco |
|  | AI452-020-0052* | **Khaertynova, Ilsiyar** | Ethic Council Under Moh Of Russian Federation  3, Rahmanovskiy Per.  Gsp-4  Moscow, 127994  Russian Federation  Republic Clinical Infectious Hospital  Pobedy prospect, 83  Kazan, Republic of Tatarstan 420140  Russian Federation  Chairpersons:  Sharifullina, GS  Chuchalin, Alexander G |
|  | AI452-020-0053* | **Esaulenko, Elena, MD, PhD** | Ethic Council Under Moh Of Russian Federation  3, Rahmanovskiy Per.  Gsp-4  Moscow, 127994  Russian Federation  Saint-Petersburg State Pediatric Medical University  2, Litovskaya Ul  Saint Petersburg, 194100  Russian Federation  Chairpersons:  Mikirtichan, Galina  Chuchalin, Alexander G |
|  | AI452-020-0054* | **Rizzetto, Mario, MD** | CE AO Citta' Salute e Scienza di Torino  C.so Bramante 88/90  Torino, 10125  Italy  A.O.U. S. GIOVANNI BATTISTA  Comitato Etico  Az. Osped. S.giovanni Battista  Corso Bramante 88  Torino, 10126  Italy (Previous)  Chairpersons:  Pileri, Alessandro, MD |
|  | AI452-020-0055* | **Bruno, Savino, MD** | Comitato Etico Milano Area B  Fondazione IRCCS Ca Granda  Ospedale Maggiore Policlinico  Via F. Sforza 28  Milano, 20122  Italy  Comitato Etico Ind. A.O. Fatebenefratelli E Oftalmico  C.so Di Porta Nuova 23  Milano, 20121  Italy (Previous)  Chairpersons:  Muserra, Gaetana, MD |
|  | AI452-020-0057* | **D'Offizi, Gianpiero, MD** | Comitato Etico Dell`Irccs Istituto Nazionale Per Le Malattie  Via Portuense 292  Roma, 00149  Italy  Chairpersons:  Moroni, Mauro, PhD |
|  | AI452-020-0058* | **Fagiuoli, Stefano, MD** | Comitato Etico Della Provincia Di Bergamo  Piazza OMS 1C  Bergamo, 24127  Italy  Azienda Ospedaliera Papa  Giovanni XXIII-Comitato di Bioetica  Piazza OMS, 1  Bergamo, 24127  Italy (Previous)  Chairpersons:  Spagnolo, Antonio |
|  | AI452-020-0059* | **Plesniak, Robert** | Komisja Bioetyczna Akademii Medycznej W Bialymstoku  Ul. Kilinskiego 1  Bialystok, 15-089  Poland |
|  | AI452-020-0062* | **Ghesquiere, Wayne, MD** | IRB Services (Institutional Review Board Services)  372 Hollandview Trail  Suite #300  Aurora, On L4G 0A5  Canada  Chairpersons:  Knight, Allan, MD (Previous)  Blajchman, Morris, MD |
|  | AI452-020-0063* | **Ramji, Alnoor, MD** | IRB Services (Institutional Review Board Services)  372 Hollandview Trail  Aurora, On L4G 0A5  Canada  Chairpersons:  Knight, Allan, MD (Previous)  Blajchman, Morris, MD |
|  | AI452-020-0064* | **Sherman, Morris, MD** | University Health Network Research Ethics Board  700 University Avenue  Hydro Place Building  10th Floor South Room 10-56  Toronto, M5G 1Z5  Canada  Chairpersons:  Mcrae, Karen, MD |
|  | AI452-020-0065* | **Tam, Edward** | Irb Services Inc.  372 Hollandview Trail  Suite 300  Aurora, On L4G 0A5  Canada  Chairpersons:  Blajchman, Morris, MD |
|  | AI452-020-0066* | **Weiss, Karl** | Hopital Maisonneuve-Rosemont  5415 Blvd De L'assomption  Montreal, Qc H1T 2M4  Canada  Chairpersons:  Senecal, Lynne, MD |
|  | AI452-020-0067 | **Box, Terry D, MD** | Schulman Associates Institutional Review Board  4445 Lake Forest Drive  Cincinnati, OH 45242  USA  Chairpersons:  Nelson, Sharon Lynn, MSN, RN, CNS |
|  | AI452-020-0068 | **Thuluvath, Paul, MD** | Mercy Medical Center Irb  345 St. Paul Place  Bunting 7th Floor  Baltimore, MD 21202  USA |
|  | AI452-020-0070 | **Cochran, Joseph Lynn, MD** | Schulman Associates Irb  4445 Lake Forest Drive  Cincinnati, OH 45242  USA  Chairpersons:  Nelson, Sharon Lynn, MSN, RN, CNS |
|  | AI452-020-0071 | **Di Bisceglie, Adrian M, MD** | Saint Louis University Institutional Review Board  3556 Caroline Street  St. Louis, MO 63104  USA |
|  | AI452-020-0072 | **Zamor, Philippe J, MD** | Copernicus Group Irb  One Triangle Dr Ste 100  PO Box 110605  Research Triangle Park, NC 27709  USA |
|  | AI452-020-0073 | **Morgan, Timothy Ross, MD** | Long Beach Vamc  (151) Research Healthcare Group  Institutional Review Board  Subcommittee On Human Subjects  5901 E Seventh St  Long Beach, CA 90822  USA |
|  | AI452-020-0074 | **Overton, Edgar T, MD** | Western Institutional Review Board  3535 Seventh Ave SW  Olympia, WA 98502  USA |
|  | AI452-020-0076 | **Tran, Tram, MD** | Cedars-Sinai Medical Center Irb  8383 Wilshire Blvd.  Suite 742  Beverly Hills, CA 90211  USA |
|  | AI452-020-0077 | **Clincea, Radu, MD** | Orlando Va Medical Center  Institutional Review Board  5201 Raymond Street  Orlando, FL 32803  USA |
|  | AI452-020-0078 | **Shiffman, Mitchell L, MD** | Schulman Associates Irb  4445 Lake Forest Drive  Cincinnati, OH 45242  USA  Schulman Associates Institutional Review Board  4445 Lake Forest Dr Ste 300  Cincinnati, OH 45242  USA (Previous)  Chairpersons:  Nelson, Sharon Lynn, MSN, RN, CNS |
|  | AI452-020-0079 | **Yangco, Bienvenido G, MD, MPH** | Schulman Associates Irb  4445 Lake Forest Drive  Cincinnati, OH 45242  USA  Chairpersons:  Nelson, Sharon Lynn, MSN, RN, CNS |
|  | AI452-020-0081 | **Ryan, Michael J, MD, FACP** | Schulman Associates Institutional Review Board  4445 Lake Forest Drive  Cincinnati, OH 45242  USA  Chairpersons:  Nelson, Sharon Lynn, MSN, RN, CNS |
|  | AI452-020-0082 | **Harrison, Stephen A, MD** | Brooke Army Medical Center  3698 Chambers Pass  IRB, Dept. of Clinical Investigation  Fort Sam Houston, TX 78234  USA |
|  | AI452-020-0083 | **Lawitz, Eric J, MD** | Schulman Associates Irb  4445 Lake Forest Drive  Cincinnati, OH 45242  USA  Chairpersons:  Nelson, Sharon Lynn, MSN, RN, CNS |
|  | AI452-020-0084 | **Ghalib, Reem H, MD** | Schulman Associates Irb  4445 Lake Forest Drive  Cincinnati, OH 45242  USA  Chairpersons:  Nelson, Sharon Lynn, MSN, RN, CNS |
|  | AI452-020-0085 | **Han, Steven-Huy, MD** | Greater Los Angeles Healthcare System  Dept Of Veterans Affairs  Investigational Review Board  11301 Wilshire Blvd Bldg 114  Los Angeles, CA 90073  USA |
|  | AI452-020-0086* | **Brunetto, Mauriziarossana, MD** | Comitato Etico Area Vasta Nord Ovest  Via Roma 67  Pisa, 56126  Italy  Comitato Etico A.O. Pisana  Via Roma 67  Pisa, 56126  Italy (Previous)  Chairpersons:  Danesi, Romano, MD |
|  | AI452-020-0087* | **Samuel, Didier, MD** | Cpp Ile De France 8 Ambroise Pare  Hopital Ambroise Pare  9 avenue Charles de Gaulle  Laboratoire d'Anatomopathologie  Boulogne Billancourt, 92100  France  Chairpersons:  Barthod, Frederique, MD (Previous)  Mussetta, Bertrand, PharmD |
|  | AI452-020-0088* | **Alric, Laurent, MD** | Cpp Ile De France 8 Ambroise Pare  Hopital Ambroise Pare  9 avenue Charles de Gaulle  Laboratoire d'Anatomopathologie  Boulogne Billancourt, 92100  France  Chairpersons:  Mussetta, Bertrand, PharmD  Barthod, Frederique, MD (Previous) |
|  | AI452-020-0090* | **Causse, Xavier, MD** | Cpp Ile De France 8 Ambroise Pare  Hopital Ambroise Pare  9 avenue Charles de Gaulle  Laboratoire d'Anatomopathologie  Boulogne Billancourt, 92100  France  Chairpersons:  Barthod, Frederique, MD (Previous)  Mussetta, Bertrand, PharmD |
|  | AI452-020-0091* | **Guyader, Dominique, MD, PhD** | Cpp Ile De France 8 Ambroise Pare  Hopital Ambroise Pare  9 avenue Charles de Gaulle  Laboratoire d'Anatomopathologie  Boulogne Billancourt, 92100  France  Chairpersons:  Mussetta, Bertrand, PharmD  Barthod, Frederique, MD (Previous) |
|  | AI452-020-0092 | **Khalili, Mandana, MD** | Committee On Human Research  3333 California Street  Office of Research, Suite 315  San Francisco, CA 94118  USA |
|  | AI452-020-0093* | **RIACHI, Ghassan, MD** | Cpp Ile De France 8 Ambroise Pare  Hopital Ambroise Pare  9 avenue Charles de Gaulle  Laboratoire d'Anatomopathologie  Boulogne Billancourt, 92100  France  Chairpersons:  Mussetta, Bertrand, PharmD  Barthod, Frederique, MD (Previous) |
|  | AI452-020-0094* | **Lurie, Yoav, MD** | Shaare Zedek Medical Center  Helsinki Committee  12 Shmuel Beit St; Pob 3235  Jerusalem, 91031  Israel |
|  | AI452-020-0095* | **Ben-Ari, Ziv** | Tel-Hashomer Hospital   Sheba Medical Center  Tel-Hashomer  Ramat Gan, 52621  Israel |
|  | AI452-020-0096* | **Zuckerman, Eli** | Lady Davis Carmel Medical Center  Helsinki Committee  7 Michal Street  Haifa, 34362  Israel |
|  | AI452-020-0097* | **Baruch, Yaacov, MD** | Rambam Medical Center  Helsinki Committee  8 Ha'alia St.bat-galim  Pob 9602  Haifa, 31096  Israel |
|  | AI452-020-0098* | **Safadi, Rifaat, MD** | Holy Family Hospital  Holy Family Hospital  P.o.box 8  Nazareth, 16100  Israel |
|  | AI452-020-0099* | **Manns, Michael, MD** | Ethik-Kommission Der Medizinischen Hochschule Hannover  Carl-neuberg-str. 1  Hannover, 30625  Germany  Chairpersons:  Troeger, HD |
|  | AI452-020-0100* | **Beckebaum, Susanne, MD** | Ethik-Kommission Der Medizinischen Hochschule Hannover  Carl-neuberg-str. 1  Hannover, 30625  Germany  Chairpersons:  Troeger, HD |
|  | AI452-020-0101* | **Berg, Thomas, MD**  **Biermer, Michael, MD (Previous)** | Ethik-Kommission Der Medizinischen Hochschule Hannover  Carl-neuberg-str. 1  Hannover, 30625  Germany  Chairpersons:  Troeger, HD |
|  | AI452-020-0102* | **Gerken, Guido, MD** | Ethik-Kommission Der Medizinischen Hochschule Hannover  Carl-neuberg-str. 1  Hannover, 30625  Germany  Chairpersons:  Troeger, HD |
|  | AI452-020-0103* | **Klass, Dietmar, MD** | Ethik-Kommission Der Medizinischen Hochschule Hannover  Carl-neuberg-str. 1  Hannover, 30625  Germany  Chairpersons:  Troeger, HD |
|  | AI452-020-0104* | **Lueth, Stefan, MD** | Ethik-Kommission Der Medizinischen Hochschule Hannover  Carl-neuberg-str. 1  Hannover, 30625  Germany  Chairpersons:  Troeger, HD |
|  | AI452-020-0105* | **Maieron, Andreas, MD** | Ethikkommission Des Landes Oberoesterreich  Landesnervenklinik Wagner-jauregg  Wagner-jauregg Weg 15  Linz, 4020  Austria  Chairpersons:  Fischer, Johannes, MD |
|  | AI452-020-0106* | **Muellhaupt, Beat, MD** | Kantonale Ethikkommission Zurich  Sonneggstrasse 12  Zurich, 8091  Switzerland  Chairpersons:  Russi, Erich |
|  | AI452-020-0107* | **Stauber, Rudolf, MD** | Ethikkommission Des Landes Oberoesterreich  Landesnervenklinik Wagner-jauregg  Wagner-jauregg Weg 15  Linz, 4020  Austria  Chairpersons:  Fischer, Johannes, MD |
|  | AI452-020-0108* | **Heim, Markus, MD**  **Heim, Markus (Previous)** | Ethikkommission Beider Basel (Ekbb)  Hebelstrasse 53  Basel, 4056  Switzerland  Chairpersons:  Perruchoud, Andre, MD |
|  | AI452-020-0109* | **Coffin, Carla S, MD** | Conjoint Health Research Ethics Board  3rd floor Mackimmie Library Tower (MLT 300)  2500 University Drive NW  Calgary, AB T2N 1N4  Canada  Chairpersons:  Page, Stacey, PhD |
|  | AI452-020-0110* | **Arenas, Juan, MD, PhD**  **Arenas, Juan, MD, PhD (Previous)** | Hospital General Universitario De Alicante  Avda. Pintor Baeza, 12  Ed. Gris Planta 3  Alicante, 03010  Spain  Hospital Donostia  Secretaria Del Ceic  Paseo Doctor Beguiristain, S/n  San Sebastian, Guipuzcoa 20014  Spain (Previous)  Chairpersons:  Horga De La Parte, Jose Francisco |
|  | AI452-020-0111* | **Lee, Tonny Mengche, MD** | Schulman Associates Irb  4445 Lake Forest Drive  Cincinnati, OH 45242  USA  Chairpersons:  Nelson, Sharon Lynn, MSN, RN, CNS |
|  | AI452-020-0112 | **Alba, Laura M, MD**  **Regenstein, Fredric Gary, MD (Previous)** | St. Luke's Hospital  4401 Wornall Road  Institutional Review Board  Kansas City, MO 64111  USA |
|  | AI452-020-0113* | **Zeuzem, Stefan, MD** | Ethikkommission Der Medizinische Hochschule Hannover  Carl-neuberg-strasse 1  Hannover, 30625  Germany  Chairpersons:  Troeger, HD |
|  | AI452-020-0114 | **Dao, Michael, MD** | Schulman Associates Irb  4445 Lake Forest Dr  Ste 300  Cincinnati, OH 45242  USA  Schulman Associates Irb  4445 Lake Forest Drive  Cincinnati, OH 45242  USA (Previous) |
|  | AI452-020-0115* | **Silvain, Christine, MD, PhD** | Cpp Ile De France 8 Ambroise Pare  Hopital Ambroise Pare  9 avenue Charles de Gaulle  Boulogne Billancourt, 92100  France  Chairpersons:  Mussetta, Bertrand, PharmD |
